# Supplementary material for: MET-PREVENT: metformin to improve physical performance in older people with sarcopenia and physical prefrailty/frailty – protocol for a double-blind, randomised controlled proof-of-concept trial
Source: BMJ Open. 2022 Jul 18;12(7):e061823. doi: 10.1136/bmjopen-2022-061823 (PMC9297211; doi:10.1136/bmjopen-2022-061823)
Supplement: Supplementary data [file bmjopen-2022-061823supp001.pdf]

Newcastle University

<local NHS Trust logo>

MET-PREVENT

Metformin to prevent progression to frailty for older people

# – a randomised controlled proof of concept trial

Introduction

Thank you for considering taking part in the MET-PREVENT study. Before you decide whether to take part, please take time to read this information about the study carefully. Feel free to discuss it with your family, friends, carers or your GP if you wish to do so. If anything is unclear, or you need more information, please ask. You will find our contact details at the back of the information. This leaflet is yours to keep.

SUMMARY of the MET-PREVENT STUDY

**Purpose of the study**	• We would like to find out whether a medicine called metformin (used to treat type 2 diabetes) can help people over the age of 65 with muscle weakness but without diabetes to become stronger and improve their daily activities.
**Will I definitely get metformin?**	• No. To ensure that the study is a fair test, a computer will randomly allocate you to either metformin or the placebo (dummy) tablets. There is an equal chance that you will receive metformin or the dummy tablets. Both types of tablet are identical. Neither you nor the doctor, nurse or study team will know which treatment you are receiving.
**What are the side effects of the medicine?**	• The medicine may cause nausea (feeling sick) or loose bowels in some people. Most people do not get either

Rennie KJ, *et al. BMJ Open* 2022; 12:e061823. doi: 10.1136/bmjopen-2022-061823

Newcastle University

<local NHS Trust logo>

of these effects. These side effects usually disappear after a few days or a couple of weeks.

If I take part, what will I have to do?

- All of the study visits may take place in your own home, at a research centre, or at a hospital clinic.
- You will be asked to attend a screening visit. The doctor will discuss the study with you and you can ask questions. We will ask to take a small blood sample to see if it is safe for you to take part.
- If you are suitable, we will ask you to take part in another 5 visits (3 of these are short safety visits).
- You will be asked to take a tablet 3 times a day for 4 months.
- We will ask you to give small blood samples at every visit (6 teaspoons), and a small stool (poo) sample at 2 visits (you can collect this in advance of the visit).
- At 2 visits we will ask you to do some simple tests that involve balance, walking a short distance, getting up from a chair and gripping a measuring device with your hand. You will also be asked to complete 3 questionnaires that ask about your daily activities.

Do I have to visit the hospital to take part?

- Not if you don't want to. We can visit you in your own home if you wish. A member of the local clinical team will bring all the equipment.

Do I have to take part?

- No – it is entirely your choice whether you wish to take part or not. Your care will not be affected in any way.

If you are still interested in taking part in the MET-PREVENT study, please read on for more information.

Rennie KJ, *et al. BMJ Open* 2022; 12:e061823. doi: 10.1136/bmjopen-2022-061823

<local NHS Trust logo>

Figure 1. Summary of the MET-PREVENT study visits

SCREENING VISIT – 1 to 2 HOURS

- Written informed consent with doctor
- Medical history, other medicines
- Physical tests (short walk, sit to stand and handgrip strength)
- Blood tests (2 teaspoons)
- After visit - eligibility for the study confirmed

BASELINE VISIT – 1 to 2 HOURS

- Have stool sample ready
- Height and weight
- Physical tests (walking, balance, sit to stand and handgrip strength)
- Light beam skin test and muscle measurement
- 3 questionnaires
- Blood samples (6 teaspoons)

1, 2 and 3 MONTH FOLLOW UPS – about 30 minutes

- Medicines review
- Review of any episodes of ill health
- Safety blood sample (2 teaspoons)

4 MONTH FOLLOW UP (FINAL) VISIT – 1 to 2 HOURS

- Have stool sample ready
- Height and weight
- Physical tests (walking, balance, sit to stand and handgrip strength)
- Light beam skin test and muscle measurement
- 3 questionnaires
- Blood samples (6 teaspoons)
- Review of other medicines
- Return unused study medicine

Rennie KJ, *et al. BMJ Open* 2022; 12:e061823. doi: 10.1136/bmjopen-2022-061823

Newcastle University

<local NHS Trust logo>

What is the purpose of the MET-PREVENT study?

Many of us lose muscle size and strength as we get older – this is called sarcopenia. People with weaker muscles are more likely to fall over and may start to have problems carrying out normal daily activities. They may also take longer to recover from other illnesses. Falling, struggling with daily living and taking a long time to get better from being unwell is a condition called frailty.

The best way to keep up your muscle strength is to do strengthening exercises, but not everyone wants to, or is able to do these.

Metformin is a medicine that is already safely used in older people to treat type 2 diabetes. Recent research suggests that metformin might help to improve muscle strength, even in people who do not have diabetes. To know if this is correct, we need to test metformin in a clinical trial. The MET-PREVENT study will test if metformin can improve muscle strength in older people who have signs of muscle weakness.

What are the possible side effects of the study medicine?

As with any medicine, the medicine used in this study (**metformin**) may cause side effects in some people. Doctors in England write millions of prescriptions for metformin every year, and the side effects are well understood.

In some people, metformin can cause nausea (occasionally vomiting), loss of appetite, tummy ache or loose bowel motions. However, we are using a low dose of metformin to reduce the chance of any of these side effects. These effects usually occur during the first few weeks of treatment and then ease in most people. To help prevent them, we will ask you to take your study medicine in 3 daily doses during or just after meals.

If you become unwell for some other reason, metformin can cause a build-up of acid in the blood. This is very rare. Occasionally, metformin may also cause a skin rash or irritate the liver. We will do regular blood tests to watch out for these problems. If you do become ill for some other reason, your doctors may stop the study medicine for a few days until you get better. This helps to prevent acid build-up in the blood. **More detail on these side effects is provided in the Supporting Information on page 10.**

Rennie KJ, *et al. BMJ Open* 2022; 12:e061823. doi: 10.1136/bmjopen-2022-061823

<local NHS Trust logo>

We will issue you with a safety card that will have your medicine dose printed on it. The safety card will also have the contact details of the study team at your local hospital. If you see other healthcare professionals, you should show them the safety card to let them know that you are taking part in a clinical study.

The 4 month follow-up visit is the last study visit. For the 4 weeks (28 days) following on from this final visit, we would like to know if you have been unwell. We will ask you to contact your study doctor or a member of their team to tell them about this. You can contact your study doctor and team on the contact details given on the last page of this information sheet.

If you are unsure whether the study doctor needs to know about an illness or not, please get in touch with your study doctor or a member of their team to check.

What would I have to do if I took part?

We will ask you to attend 6 visits, either at hospital or in your own home:

- Screening visit
- Baseline visit
- 1, 2 and 3 month safety follow-up visits
- 4 month follow-up visit

These visits are extra, and not part of your usual NHS care. There is a diagram (Figure 1) on page <insert page number>that shows the details of the trial visits.

Screening Visit

At the screening visit the doctor will discuss the study with you, and you will be able to ask questions. If this visit takes place in your home, a member of the research clinical team (usually a research nurse) will use a phone to let you talk to the doctor. This may be over a videolink. We will then ask if you are happy to give written consent to take part in the study. We will ask you some questions about yourself and your health. We will also ask to take a small blood sample (2 teaspoons) to make sure that it is safe for you to take part in

<local NHS Trust logo>

this study. We will ask you to walk a short distance (4 metres), stand up and sit down from a chair five times, and test your handgrip strength.

We will ask you if you are able to open child-resistant caps on medicine bottles

When we have the results of the blood test, we will call you and arrange the next visit.

Baseline Visit

We will ask you to take part in a number of assessments before you start the study medicine. We will ask that you have a small stool (poo) sample ready in advance. We will ask you to do the following:

- We will measure your weight and height.
- Walk a short distance (4 metres), with your usual walking aids.
- Walk for 6 minutes, with your usual walking aids.
- To test your balance, we will ask you to stand beside the researcher for a short time (no more than 30 seconds).
- Stand up from a chair.
- Use a handgrip on both hands.
- Put sticky pads on your hand and foot connected to a device that measures the amount of muscle in your body.
- Measure the response of your skin to shining a light beam on it. This device tells us about different substances inside your skin without needing to take a skin sample).
- Complete 3 questionnaires that ask how you feel about your health and your activities of daily living (such as washing and cooking).
- Have a small amount of blood taken (6 teaspoons).
- Ask about other medicine that you may be taking.

The study medicine will then be issued to you. If you are attending hospital, there will be a short wait for this. If the baseline visit takes place in your own home, or if the wait at hospital may be too long, the study medicine will be posted to you via Royal Mail or delivered by local courier. A member of the study team will phone you to check that you have received the medicine. They will also check that you can open the bottle, and have taken the first dose.

Rennie KJ, *et al. BMJ Open* 2022; 12:e061823. doi: 10.1136/bmjopen-2022-061823

<local NHS Trust logo>

1, 2 and 3 month follow up visits

These visits are to check that it is safe for you to continue in the study. To do this, we will ask you for a small blood sample (2 teaspoons) at each visit. We will ask you for a list of all of your other medicines that you take. We will also collect information about all episodes of ill-health that you have experienced in the past month.

4 month follow up visit

This the last study visit. We will ask you to have a stool (poo) sample ready. We will ask you to repeat all of the tests that you did at the baseline visit, including the blood samples. We will also collect any unused trial medicine.

What are the benefits and disadvantages of taking part in this study?

We cannot promise the study will help you directly. However, the information we collect from this study may help to improve the treatment for people with muscle weakness. If you want to find out more about taking part in research studies, you can visit the NHS Choices website www.nhs.uk.

As with any medicine, metformin can cause side effects in some people. We are using a low dose to reduce the chance of this happening. If you do get a side effect from the study medicine, your doctor can stop your medicine. The side effects should disappear rapidly. In case of an emergency, the doctors can find out which treatment you are taking (metformin or placebo) if they need to.

Is it safe for me to take part in this study during COVID-19?

The study will only take place if your hospital says that it is safe to do so. You can take part in the study in your own home if you wish to avoid visiting the hospital. The study team will follow all of the hospital COVID-19 policies and procedures when they visit you, including wearing masks, gloves and visors. These will be explained to you when your appointments are arranged.

Rennie KJ, *et al. BMJ Open* 2022; 12:e061823. doi: 10.1136/bmjopen-2022-061823

Newcastle University

<local NHS Trust logo>

What happens to my blood and stool samples?

At the baseline and 4 month visits, giving the stool (poo) sample and the large blood sample (4 teaspoons) is optional. We still need a small blood sample for safety tests (2 teaspoons). The samples will be stored in a licenced Newcastle University Biobank at the end of the study. **Your consent to store these additional samples in the biobank is optional.** Your biobank samples may be used in further research linked to this study. They may also be used by other researchers in different research studies. Your samples can only be identified by using your unique study identity number. Researchers who use your samples will not know who you are.
Other researchers may want to use parts of your biobank sample in animal or commercial (paid) research. You can give samples to the biobank, but opt out of them being used for animal or paid research.

Pregnancy

We know that female participants over the age of 65 years cannot become pregnant.

However, if you are a male participant, we will ask you to inform us if your female partner becomes pregnant, or is breast-feeding an infant. We will ask for your consent to do this. We will also ask your partner to sign a consent form. This will allow the study team to collect safety information about their pregnancy and their baby.

What happens at the end of the study?

At the end of the study (4 months) you will stop taking the study medicine. You will continue to receive standard care like any other patient with your condition under the care of your GP and/or hospital doctor.

When everyone has completed the study, we will analyse the results and we will tell you what the results are. We can either invite you to a study event to or send you a written newsletter – whichever you prefer.

Rennie KJ, *et al. BMJ Open* 2022; 12:e061823. doi: 10.1136/bmjopen-2022-061823

<local NHS Trust logo>

We would like to follow your progress with your health over the next 5 years. We will do this by looking at your medical records, and we will ask your permission to do this. You do not have to do anything else.

Who do I contact for further information?

We will be happy to answer any questions you, your family or your carers may have about any aspect of this clinical study. Please call the number at the end of this booklet to speak to the Research Nurse at your local hospital.

Newcastle University

<local NHS Trust logo>

SUPPORTING INFORMATION

Metformin side effects

Very common – nausea, vomiting, diarrhoea, abdominal pain and loss of appetite (this usually stops in a few weeks in most people).

Common – taste disturbance.

Very rare – lactic acid build up in the blood

Very rare - abnormalities in liver function tests or hepatitis (goes away after metformin is stopped).

Very rare – skin redness, itching, hives.

Very rare – decreased vitamin B12 absorption (only seen with long term use of metformin or in patients who already have a vitamin B12 absorption problem called megaloblastic anaemia)

Why was I contacted?

We have contacted you because you are aged 65 or over and our measurements or questionnaire results suggest that your muscles are not as strong as they used to be. This trial is testing a medicine (metformin) to see if we can prevent further weakening of muscles in people like you.

Do I have to take part?

No - it is up to you to decide whether or not to take part in this study. You do not have to take part. If you choose not to, you will continue to get the standard care arranged by your doctor.

If you agree to take part, we will ask you to sign a consent form. We will give you a copy of your signed consent form which is yours to keep. You can still change your mind and withdraw at any time without having to give a reason. If you decide not to take part, or withdraw from the study later on, your current or future medical care will not be affected in any way.

What does giving consent mean for me?

By signing a consent form, this means that you fully understand what taking part in the study means for you. That's why it is really important that you take as much time as you want to read this information sheet. Feel free to discuss

Rennie KJ, *et al. BMJ Open* 2022; 12:e061823. doi: 10.1136/bmjopen-2022-061823

Newcastle University

<local NHS Trust logo>

the study with your family, friends or any healthcare professional. At the screening visit, you will discuss the study with a study doctor, who will answer all of your questions.

Will I know what treatment I am on?

No. A computer will decide whether you will be given either the active medicine (metformin) or the placebo (dummy) tablets for 4 months. There is an equal chance that you will be given metformin or placebo tablets. The metformin and placebo tablets look identical. This means that neither you, the researchers, nor your medical teams will know what tablets you are taking. If no-one knows what tablets you are taking, no-one can influence the results of the study.

At the end of the study, when everyone has completed their visits, you may find out which tablets you were taking. We will also write to your GP to tell them what tablets you were taking. We will ask your permission to do this.

Who has checked the scientific quality of the MET-PREVENT study?

Independent experts at Newcastle University have checked the quality of the science used to plan this study. The study has also been checked and approved by a research ethics committee (North West - Liverpool Central Research Ethics Committee). The ethics committee ensures that when you take part in the study, your rights and wellbeing will be protected. The study has also been checked by the government Medicines and Healthcare products Regulatory Agency (MHRA). The MHRA are responsible for approving all studies involving medicines. The Health Research Authority gives final overall approval for the study. The Newcastle upon Tyne Hospitals NHS Foundation Trust is the study Sponsor, which means that they have overall responsibility for the study. The Sponsor has carefully checked all of the study documentation. The Sponsor has also assessed the risks of this study. This is to ensure that we are not doing anything harmful to you during the study and that your information is collected safely and stored securely.

What happens if relevant new information becomes available?

Rennie KJ, *et al. BMJ Open* 2022; 12:e061823. doi: 10.1136/bmjopen-2022-061823

<local NHS Trust logo>

During the course of the study, if new information on the risks or benefits of metformin becomes available, we will let you know at your next study visit. If this new information requires urgent action, we will contact you before your next visit. If necessary, we will then discuss whether you should or would like to withdraw from the study.

Will you tell my GP that I'm taking part in a clinical study?

With your permission, we will write to your GP to tell them that you are taking part in this study. Your hospital medical record will also show that you are taking part in a clinical study. It is important for your safety that that your GP practice and hospital medical records show that you took part in a clinical study. If we discover a new health problem during the study, we will tell you. With your permission, we will also tell your GP. Any blood test results from taking part in this study will also be added to your medical records. Your GP will be asked to let the study team know of any side effects from taking the medicine or if you have had any emergency hospital admissions.

Your GP will not know if you have received active treatment (metformin) or placebo until after the end of the study.

Who has overall responsibility for the study?

The study Sponsor is the Newcastle upon Tyne Hospitals NHS Foundation Trust, who have overall responsibility for the study.

The doctor in charge of the study (the Chief Investigator) is Professor Miles Witham, a Consultant Geriatrician based in Newcastle upon Tyne.

The Newcastle University Clinical Trials Unit manages the study on behalf of the Sponsor.

Who is providing the study drug?

We have paid a company called ModePharma to make the metformin and placebo (dummy) tablets for this study. ModePharma specialise in making medicines for studies but they do not own the rights to make Metformin. ModePharma do not make profits by selling metformin. Your hospital pharmacy will give you the study medication (metformin or placebo) at the

Rennie KJ, *et al. BMJ Open* 2022; 12:e061823. doi: 10.1136/bmjopen-2022-061823

Newcastle University

<local NHS Trust logo>

end of the baseline visit. If your visit takes place at home, the tablets will be posted to you by Royal Mail Special Delivery or delivered by local courier. We will ask for your permission to send your name and address to the Royal Mail or the local courier.

What will happen to the results of the MET-PREVENT study?

- The results will be published in medical journals and presented at meetings to other doctors, nurses, researchers and patients.
- A report will be written for the study funder.
- A report must be written for the European Union Drug Regulating Authorities Clinical Trials (EudraCT) database.
- All study results that are published will be anonymous. This means that no-one will be able to find out who you are. Your identity will always be protected.
- The results will be available at the end of the study through publications, in the wider press and directly to patient groups.
- Fully anonymised data may be made available to other researchers to help inform other research studies.

What if I have a complaint or a problem occurs?

a) Complaints

If you have any concern or complaint about any aspect of this clinical study, please contact your local study team by phone, letter or email. Their contact details are listed at the end of this information sheet. If you are still unhappy and wish to raise your concerns with someone who is not directly involved in your care, you can contact <site to localise with local details such as local PALS phone number and email address>

You may also contact Patient Advice and Liaison Service (PALS) for confidential advice on any aspect of care on 0800 032 0202.

b) Harm

Newcastle University

<local NHS Trust logo>

In the unlikely event that you are harmed during the study and this is due to NHS staff neglect, you may have grounds for legal action and compensation. This is organised through the NHS Indemnity (insurance) scheme. You may need to pay for your own legal costs. NHS Indemnity does not offer no-fault compensation (for harm that is not anyone's fault).

The Newcastle Clinical Trials Unit, part of Newcastle University, are managing the study on behalf of the study NHS Sponsor. Newcastle University also have indemnity arrangements. This covers Newcastle University staff involved in designing and managing the MET-PREVENT study.

Will my taking part in the study be kept confidential?

Yes. All the information that you provide during the course of this study will be securely stored. Paper copies of your study information will be stored in locked files or rooms at your local hospital. Electronic copies of your study information will be stored on a secure, password-protected computer database provided by Sealed Envelope™. Only authorised members of the study team will be granted access to the database.

- At study visits, your name will not be written on completed test forms or questionnaires. Instead, we will use a study code number (called a Participant Unique Study Identifier). This number is unique for you. No-one else taking part in the study will have this number. This number will also be used in the study database. Only the study team at your hospital will be able to link this number back to you using your date of birth, name and NHS number
- The study team at your hospital will have access to your information during the study. They will use this information to contact you to organise study visits as well as for ongoing safety.
- If you opt for study visits to take place in your own home, your hospital pharmacy will have access to your contact details to post the study medicine
- Your contact details will never be shared with anyone outside of the study. The exception is Royal Mail or a local courier. You will be asked to consent to the postal service or local courier your local hospital uses having access to your contact details. This is so that they can deliver the

Rennie KJ, *et al. BMJ Open* 2022; 12:e061823. doi: 10.1136/bmjopen-2022-061823

Newcastle University

<local NHS Trust logo>

study medicine to your home address. The postal or courier services will not know you are in the study, just that they need to deliver a package to you
- You will not be named in any results, reports or on websites
- Very occasionally, information might be given during the study that, by law, we must pass on to others. For instance, information which suggested you or others were at risk of harm. In this case, confidentiality would be broken so that we could pass this information to the relevant people. You would be informed of this.
- At the end of the study, all study information will be kept in a secure storage area for at least 15 years. This is called archiving. Archiving means that any queries about the running of the study can still be answered after the study has ended. All information will be held securely to make sure we protect your confidentiality. After the archiving period has ended, your information will be safely destroyed.
- If there are any unexpected serious side effects to the medicine, we would send details of this to the government medicines agency (MHRA). There is a specific form to do this, and only your study number will be sent to them.

Will you look at information from my existing medical records?

Yes. The study team at your hospital will be able to look at your GP and hospital medical records. They need to do this to collect information that is needed for you to take part in the study. For example, they will collect results of your blood tests, and prescriptions and health history.

Authorised people from your local NHS Trust, the MHRA, Sponsor (Newcastle upon Tyne Hospitals NHS Foundation Trust) and/or the Newcastle Clinical Trials Unit will also need to look at your medical records. This is to check that the study is being carried out to the correct standards. Everyone who looks at your medical records will have a duty of confidentiality to you as a research participant.

What will happen if I don't want to carry on with the study?

Rennie KJ, *et al. BMJ Open* 2022; 12:e061823. doi: 10.1136/bmjopen-2022-061823

Newcastle University <local NHS Trust logo>

You can withdraw from the study completely at any time, for any reason. You do not have to tell the study team why you want to withdraw. You will always be fully cared for and supported in line with your GP and hospital's standard practice.
If you do give a reason for withdrawing from the study, we will ask if you are happy for us to record why you decided to withdraw.

If you withdraw from the study, we will keep the information about you that we have already collected. You are free to request that the study team destroys all information donated by you. By destroying your information, this means that it cannot be used at all for the remainder of the study. However, if some of your information has already been used in calculations and reports, it would not be possible to remove that information from these.

If I stop taking the medicine, do I have to leave the trial?

If you become unwell, your doctor may ask you to stop taking the study medicine. This may be just for a short while until it is safe for you to start taking it again.

You can stop taking the study medicine altogether, but stay in the study and continue with the study visits and assessments. We will always ask you what you would prefer to do.

What happens if I lose the capacity to consent during the study?

During the study, if you lose the capacity to make your own decisions, we will stop your medicine. The doctor may decide that you need to be withdrawn from the study. We will keep your information that has been collected up to this point.

If your doctor thinks you have recovered and can make your own decisions again, we will ask you if you want to continue to take part in the study.

Who is funding the MET-PREVENT study?

<local NHS Trust logo>

The National Institute for Health Research (NIHR) Newcastle Biomedical Research Centre funds the study. The UK government funds the NIHR to carry out research for the benefit of the NHS and its patients.

How have patients and the public been involved in the design of the study?

Volunteers from the local VOICE organisation (https://www.voice-global.org/) helped to design this study. They have also looked at the patient information sheet. We will ask a member of the public to join our study management group meetings.

Will my expenses be reimbursed?

Yes – we will pay for your travel expenses including providing a taxi if you need this to attend study visits at the hospital. Alternatively, transport may be arranged for you if your local hospital is able to offer this. Your local study team will manage any payments to reimburse costs to you and you may be asked to provide receipts for your travel.

Will I be paid for taking part?

We will not give you a payment for taking part, but we will pay for your transport and make sure that you have some food and drink at each trial visit.

HOW WILL WE USE INFORMATION ABOUT YOU?

Where 'we' is stated below, this means the study Sponsor – the Newcastle upon Tyne Hospitals NHS Foundation Trust.

We will need to use information from you, your medical records and your GP for this clinical research study.

This information will include your initials, date of birth, NHS number, name and contact details. People will use this information to do the research or to check your records to make sure that the research is being done properly.

Newcastle University

<local NHS Trust logo>

People who do not need to know who you are will not be able to see your name or contact details. Your data will have a code number, called your unique study identifier, instead.

We will keep all information about you safe and secure.

Once we have finished the study, we will keep some of the data so we can check the results. We will write our reports in a way that no-one can work out that you took part in the study.

The Sponsor will provide Royal Mail or a local courier with your name and the address to allow Royal Mail or the courier to deliver your study medicine to you. Royal Mail and the local courier have their own policies about keeping personal information that comply with UK law. This also covers how they destroy your information. The policy for the Royal Mail Group and any local courier is to only keep information for as long as it is required for the purpose for which they use it.

What are your choices about how your information is used?

- You can stop being part of the study at any time, without giving a reason, but we will keep information about you that we already have.
- We need to manage your records in specific ways for the research to be reliable. This means that we won't be able to let you see or change the data we hold about you.
- If your medical condition does not affect your existing insurance policies, then taking part in a clinical trial should not affect your insurance. For existing insurance, or if you make a new application for any kind of insurance, then you must answer the insurer's questions honestly and accurately.

Where can you find out more about how your information is used?

You can find out more about how we use your information:

- www.hra.nhs.uk/information-about-patients/

Rennie KJ, *et al. BMJ Open* 2022; 12:e061823. doi: 10.1136/bmjopen-2022-061823

Newcastle University <local NHS Trust logo>

- our leaflet available from https://www.newcastle-hospitals.nhs.uk/help/privacy/privacy-notice-for-patients/ and https://www.qegateshead.nhs.uk/research
- by asking one of the study team
- by sending an email to the trial Sponsor Data Protection Officer at nuth.dpo@nhs.net

Further Information and contact details

If you have any further questions or would like further information about the study or rights of participants, please feel free to contact the people below.

They are also who you or a doctor should contact in the event of an emergency.

<insert local Research Nurse name and contact details>

<insert local site PI name and contact details>

Thank you for reading this information sheet

FUNDED BY NIHR | National Institute for Health Research

This research is funded by the National Institute for Health (NIHR) Newcastle Biomedical Research Centre (BRC). The views expressed are those of the author(s) and not necessarily those of the NIHR or the Department of Health and Social Care.
