# Supplementary material for: MET-PREVENT: metformin to improve physical performance in older people with sarcopenia and physical prefrailty/frailty – protocol for a double-blind, randomised controlled proof-of-concept trial
Source: BMJ Open. 2022 Jul 18;12(7):e061823. doi: 10.1136/bmjopen-2022-061823 (PMC9297211; doi:10.1136/bmjopen-2022-061823)
Supplement: Author's manuscript [file bmjopen-2022-061823.draft_revisions.pdf]

BMJ Open is committed to open peer review. As part of this commitment we make the peer review history of every article we publish publicly available.

When an article is published we post the peer reviewers' comments and the authors' responses online. We also post the versions of the paper that were used during peer review. These are the versions that the peer review comments apply to.

The versions of the paper that follow are the versions that were submitted during the peer review process. They are not the versions of record or the final published versions. They should not be cited or distributed as the published version of this manuscript.

BMJ Open is an open access journal and the full, final, typeset and author-corrected version of record of the manuscript is available on our site with no access controls, subscription charges or pay-per-view fees (http://bmjopen.bmj.com).

If you have any questions on BMJ Open's open peer review process please email info.bmjopen@bmj.com

BMJ Open

MET-PREVENT: Metformin to improve physical performance in older people with sarcopenia and physical prefrailty/frailty – protocol for a double blind, randomised controlled proof of concept trial.

Journal:	*BMJ Open*
Manuscript ID	bmjopen-2022-061823
Article Type:	Protocol
Date Submitted by the Author:	07-Feb-2022
Complete List of Authors:	Rennie, Katherine ; Newcastle Clinical Trials Unit<br>Witham, Miles; Newcastle University, NIHR Newcastle Biomedical Research Centre<br>Bradley, Penny; Newcastle Upon Tyne Hospitals NHS Trust, Pharmacy directorate<br>Clegg, Andrew; University of Leeds, Academic Unit of Elderly Care & Rehabilitation<br>Connolly, Stephen; Patient and Public Involvement Representative<br>Hancock, Helen; Newcastle University, Newcastle Clinical Trials Unit<br>Hiu, Shaun; Newcastle University Institute for Health and Society, Newcastle University<br>Marsay, Leanne; Newcastle University, Newcastle Clinical Trials Unit<br>McDonald, Claire; Gateshead Health NHS Foundation Trust<br>Robertson, Laura; Newcastle University, Newcastle Clinical Trials Unit<br>Simms, Laura; Newcastle University, Newcastle Clinical Trials Unit<br>Steel, Alison; Newcastle University, Newcastle Clinical Trials Unit<br>Steves, Claire J.; King's College London<br>Storey, Bryony; Gateshead Health NHS Foundation Trust<br>Wason, James; Newcastle University, Population Health Sciences Institute<br>Wilson, Nina; Newcastle University Faculty of Medical Sciences, Population Health Sciences Institute<br>von Zglinicki, Thomas ; Newcastle University, Campus for Ageing and Health<br>Sayer, Avan; Newcastle Upon Tyne Hospitals NHS Foundation Trust, NIHR Biomedical Research Centre
Keywords:	GERIATRIC MEDICINE, CLINICAL PHARMACOLOGY, REHABILITATION MEDICINE

SCHOLARONE™
Manuscripts

MET-PREVENT: Metformin to improve physical performance in older people with sarcopenia and physical prefrailty/frailty – protocol for a double blind, randomised controlled proof of concept trial.

Katherine J Rennie*[1]

Miles D Witham*[2]

Penny Bradley[3]

Andrew P Clegg[4]

Stephen Connolly[5]

Helen Hancock[1]

Shaun Hiu[6]

Leanne Marsay[1]

Claire McDonald[7]

Laura Robertson[1]

Laura Simms[1]

Alison J Steel[1]

Claire J Steves[8]

Bryony Storey[7]

James Wason[6]

Nina Wilson[6]

Thomas von Zglinicki[9]

Avan A Sayer[2]

*joint first author.

1. Newcastle Clinical Trials Unit, Newcastle University, Newcastle upon Tyne, NE2 4AE, UK.

2. AGE Research Group, NIHR Newcastle Biomedical Research Centre, Newcastle University and Newcastle upon Tyne Hospitals NHS Foundation Trust, Newcastle, UK

For peer review only - http://bmjopen.bmj.com/site/about/guidelines.xhtml

3. Pharmacy Directorate, Newcastle upon Tyne Hospitals NHS Foundation Trust, Newcastle Upon Tyne NE7 7DN

4. Academic Unit for Ageing and Stroke Research, University of Leeds, Bradford Royal Infirmary, Bradford, BD9 6RJ, UK.

5. Patient and Public Involvement Representative

6. Population Health Sciences Institute, Newcastle University, Newcastle upon Tyne, NE2 4AX, UK.

7. Queen Elizabeth Hospital, Gateshead Health NHS Trust, Gateshead, NE9 6SX, UK.

8. Kings College London and St. Thomas' Hospital, London, SE1 7EH, UK.

9. Ageing Research Laboratories, Newcastle University, Newcastle upon Tyne, NE4 5PL, UK.

Correspondence to:

Professor Miles D Witham, NIHR Newcastle Biomedical Research Centre, Newcastle University Campus for Ageing and Vitality, Newcastle upon Tyne, NE4 5PL, UK. Tel: +44 191 208 1317. Email: Miles.Witham@newcastle.ac.uk

Word count (abstract): 300

Word count (main text): 4892

Tables: 2

Figures: 2

For peer review only - http://bmjopen.bmj.com/site/about/guidelines.xhtml

Abstract

Introduction

Skeletal muscle dysfunction is central to both sarcopenia and physical frailty, which are associated with a wide range of adverse outcomes including falls and fractures, longer hospital stays, dependency, and the need for care. Resistance training may prevent and treat sarcopenia and physical frailty, but not everyone can or wants to exercise. Finding alternatives is critical to alleviate the burden of adverse outcomes associated with sarcopenia and physical frailty. This trial will provide proof of concept evidence as to whether metformin can improve physical performance in older people with sarcopenia and physical prefrailty or frailty.

Methods and analysis

MET-PREVENT is a parallel group, double-blind, placebo-controlled proof of concept trial. Trial participants can participate from their own homes, including completing informed consent and screening assessments. Eligible participants with low grip strength or prolonged sit-to-stand time together with slow walk speed will be randomised to either oral metformin hydrochloride 500mg tablets or matched placebo, taken three times a day for four months. The recruitment target is 80 participants from two secondary care hospitals in Newcastle and Gateshead, UK. Local primary care practices will act as Participant Identification Centres (PICs). Randomisation will be performed using a web-based minimisation system with a random element, balancing on sex and baseline walk speed. Participants will be followed up for four months post-randomisation, with outcomes collected at baseline and four months. The primary outcome measure is the four metre walk speed at the four month follow-up visit.

Ethics and dissemination

The trial has been approved by the Liverpool NHS Research Ethics Committee (20/NW/0470), the Medicines and Healthcare Regulatory Authority (EudraCT 2020-004023-16), and the UK Health

For peer review only

For peer review only - http://bmjopen.bmj.com/site/about/guidelines.xhtml

Research Authority (IRAS 275219). Results will be made available to participants, their families, patients with sarcopenia, the public, regional and national clinical teams, and the international scientific community.

Trial Registration

ISRCTN29932357

Keywords

Frailty, sarcopenia, older people, metformin, clinical trial.

Strengths and limitations of the study

- Seeks to enrol older people with sarcopenia and physical prefrailty or frailty – an under-served group
- Wide-ranging mechanistic studies embedded in the trial
- Flexible recruitment methods and flexible study visits including option for participants to be seen at home
- Limitations of short-term follow up and small sample size
- Will exclude some people with multimorbidity due to safety considerations

For peer review only

Background

Skeletal muscle dysfunction is central to both sarcopenia and physical frailty, which are common syndromes in older people. Sarcopenia, the loss of muscle strength and mass that commonly accompanies ageing [1] is a major health problem for many older people. Sarcopenia is a major risk factor for falls, hospitalisation, increased length of stay, care home admission and earlier death [2-4]. In addition, sarcopenia is an important component of the physical frailty syndrome, which is associated with a similar range of adverse outcomes [5]. Physical frailty is typically defined as the presence of three or more of five physical characteristics (weight loss, low energy expenditure, exhaustion, slow gait speed and low grip strength) [6]. The presence of one or two of these characteristics indicates the presence of physical prefrailty as a precursor to physical frailty. Impaired physical performance is therefore a key characteristic of both sarcopenia and physical frailty and is a target for intervention that is prioritised by patients [7].

At present, resistance exercise training is the only intervention proven to improve outcomes for people with sarcopenia or physical frailty, with limited evidence on its effects in physical prefrailty [8-10]. Not all older people with sarcopenia or physical frailty are able or willing to undertake resistance training. As a result, alternative therapeutic options are needed to both prevent and improve sarcopenia and physical frailty, including through targeting physical prefrailty as a precursor state to more advanced physical frailty. Although the aetiology of sarcopenia and physical frailty are incompletely understood, it is becoming clear that multiple fundamental biological pathways related to ageing are important in driving these syndromes. Key pathways include inflammation, mitochondrial dysfunction, neuromuscular junction dysfunction, cellular senescence, and dysregulation of nutrient sensing and intracellular metabolism [1,11].

Metformin, a biguanide molecule, has been used as a treatment for type 2 diabetes mellitus for decades; it is a generally safe and well tolerated therapy even in older people with physical frailty or

For peer review only - http://bmjopen.bmj.com/site/about/guidelines.xhtml

multiple health conditions. Importantly, the mode of action of metformin in lowering glucose in patients with type 2 diabetes does not rely on stimulating insulin release, and thus users are not subjected to an increased risk of hypoglycaemia. Metformin has pleiotropic effects on glucose metabolism and energy utilisation as well as on a range of other age-related pathophysiological pathways; several of these actions may be beneficial in treating the muscle dysfunction seen in sarcopenia and physical frailty as summarised in Figure 1 [12-23].

Observational studies show lower rates of cardiovascular events and cancer mortality in metformin users [24,25], consistent with a wide range of physiological effects as outlined above. Few studies have examined the relationship between metformin use, sarcopenia and physical frailty. Such observational studies are challenging because metformin is currently indicated for use in diabetes mellitus – itself implicated in accelerated ageing, skeletal muscle dysfunction and earlier onset of physical frailty. However, incident and prevalent frailty-related diseases (including falls, weight loss, gait disorders, and frailty diagnosed using a cumulative deficits frailty index) have been found to be less common in patients with type 2 diabetes treated with metformin compared to those treated with other glucose-lowering agents in observational studies [25,26].

One previous trial conducted in Indonesia studied people aged 60 and over with prefrailty (defined by either the Fried criteria or by a cumulative deficits index) but without diabetes [27]. Participants were randomised to receive 16 weeks of 500mg metformin thrice daily or matched placebo. Walk speed increased significantly in the treatment group compared to placebo (by 0.13m/s; which exceeds the minimum clinically important difference of 0.10m/s [28]). The recent MASTERS randomised trial did not show evidence that metformin enhanced the effect of resistance training, but the focus of this trial (augmentation of resistance training) was different and did not target people with sarcopenia or physical frailty [29].

For peer review only - http://bmjopen.bmj.com/site/about/guidelines.xhtml

Trial objectives

MET-PREVENT is a parallel group, randomised, double-blind, placebo-controlled trial. The primary objective of MET-PREVENT is to provide proof of concept evidence as to whether metformin is superior to placebo in improving physical performance in older people with sarcopenia and physical prefrailty or frailty. The secondary objectives are to elucidate potential mechanisms of action of metformin on sarcopenia and physical frailty by correlating changes in biomarkers with changes in physical performance measures between baseline and four months. A placebo-controlled design has been selected to minimise bias and is appropriate given the absence of other pharmacological interventions for sarcopenia or physical frailty.

Methods: Participants, interventions and outcomes

Trial Setting

Participants will be recruited from the catchment areas of two secondary care hospitals in the North-East of England. Participants will be approached through Older Peoples Medicine Clinics, Day Units, assessment units and rehabilitation facilities at the hospitals. In addition, local general practices (GPs) will act as Participant Identification Centres (PICs), a registry of patients with sarcopenia [30] and the National Institute for Health Research BioResource will be used to identify additional potential participants. A flexible approach to the setting for participation will be offered, with the option of either research centre, clinic or the participant's own home as the setting for trial visits.

Eligibility criteria

The target population for MET-PREVENT is older adults (≥65 years) with sarcopenia and either physical prefrailty or frailty. Sarcopenia is defined using the handgrip strength and sit to stand thresholds from the 2019 European Working Group on Sarcopenia in Older People (EWGSOP) guidance [31]. Low muscle mass is not used as an eligibility criterion in this trial; the 2019 EWGSOP guidance allows a diagnosis of probable sarcopenia without muscle mass measurement. Muscle mass is not routinely

For peer review only - http://bmjopen.bmj.com/site/about/guidelines.xhtml

measured in clinical practice, and the omission of this criterion will improve the relevance of the trial results to clinical practice. Physical prefrailty and frailty are defined following the method of Fried et al [6]; participants will have a minimum of two of the five Fried criteria (and thus fulfil the definition for prefrailty) but may have three or more criteria (fulfilling the definition of frailty). The MET-PREVENT eligibility criteria are listed in Table 1. Exclusion criteria are primarily designed to exclude participants at higher risk of adverse events from taking metformin, those where metformin therapy is already indicated (e.g. diabetes mellitus), and other types of skeletal myopathy (e.g. steroid myopathy, heart failure myopathy) where confusion with sarcopenia might arise.

Table 1. MET-PREVENT inclusion and exclusion criteria

**Inclusion Criteria**
• Adults aged ≥ 65 years
• Low maximum handgrip strength (<16kg for women, <27kg for men) OR 5x sit to stand time >15 seconds
• Slow walk speed (<0.8 m/s on 4 metre walk test).

**Exclusion Criteria**
• Diabetes mellitus (type 1 or type 2)
• Previous intolerance of metformin or taking metformin for another condition
• Any contraindication to metformin, as listed in the current Summary of medicinal Product Characteristics for metformin
• Any medication which significantly interacts with metformin, as listed in the current Summary of medicinal Product Characteristics for metformin
• eGFR <45ml/min/1.73m^2 by MDRD4 or CKD-EPI equation
• History of diarrhoeal illness within the last three months (>48 hours of Bristol stool chart grade 6 or 7)
• Alcohol intake >21 units/week (women) or >35 units/week (men)

For peer review only - http://bmjopen.bmj.com/site/about/guidelines.xhtml

- Symptomatic chronic heart failure, diagnosed according to European Society of Cardiology guidelines (asymptomatic left ventricular systolic dysfunction will not be an exclusion criterion)
- Liver function tests (bilirubin, alanine aminotransferase or alkaline phosphatase) >3x upper limit of normal
- Oral steroid dose >7.5mg prednisolone equivalent per day
- Unable to mobilise without human assistance
- Unable to give written informed consent
- Life expectancy of <3 months as adjudicated by the local Investigator
- Participation in other interventional studies within 30 days prior to trial entry. Co-enrolment with other interventional studies is not allowed (observational studies and registries are permitted

Interventions

All randomised participants will take either metformin hydrochloride (500mg film-coated) tablets or matched placebo tablets, orally, three times a day with food or just after a meal. No dose adjustments are planned. Study medication is dispensed in a single bottle which is identical for metformin and placebo.

Investigators may discontinue the trial treatment in the event of side effects occurring, that are possibly, probably or definitely related to trial medication and which are not tolerable to the participant, or which constitute a serious adverse reaction (SAR) or suspected unexpected serious adverse reaction (SUSAR). Treatment will also be discontinued if the participant requests the medication to be withdrawn; in the event of a new diagnosis of diabetes mellitus (type 1 or type 2) or symptomatic chronic heart failure; if the estimated glomerular filtration rate (GFR) falls below 30ml/min/1.73m^2 or plasma lactate concentration is >4mmol/L in the safety follow up visit blood tests, or if lactic acidosis is diagnosed as part of clinical workup for an intercurrent illness. The trial medication may be temporarily discontinued at the instigation of the treating clinical team or the study team if an episode of acute kidney injury or an intercurrent illness with a risk of dehydration or hypovolemia occurs. Once this has resolved, trial medication may be restarted if the estimated GFR is

For peer review only - http://bmjopen.bmj.com/site/about/guidelines.xhtml

>30ml/min/1.73m^2. Participants who discontinue allocated trial medication and wish to remain in the trial will be followed up as per their allocated treatment intervention arm. If a participant does not want to attend any further trial visits but is willing to complete questionnaires by phone/post then they may continue in the trial.

Outcomes

Primary Outcome

The primary outcome is the between-group difference in the four metre walk speed at four months. The four metre walk speed is performed from a standing start, with the participant asked to walk at their usual pace. Short-course walking speed is a powerful predictor of a range of adverse outcomes in older people, including death, dependency and cognitive impairment [32-34]. The four metre walk speed has been used as an outcome measure in previous trials, including those of metformin [29] and the minimum clinically important difference for this measure has been estimated to be 0.1m/s [27].

Secondary Outcome Measures

Secondary outcome measures in the MET-PREVENT trial include other measures of physical performance, health-related quality of life, mechanistic outcomes and outcomes related to trial performance. Maximum handgrip strength will be measured using a Jamar dynamometer [35]; three readings will be taken for each hand and the highest reading used. Six-minute walk distance will be recorded over a 10m course with standardised encouragement [36]. The Short Physical Performance Battery (SPPB) [37] will be measured as a composite test of lower limb function with the five-times sit to stand time performed as part of the SPPB analysed as an additional, separate outcome. Lean body mass will be estimated using the Akern 101 bioimpedance system (Akern, Pontassieve, Italy), with the Sergi equation used to derive measures of total lean body mass [38]. The number of physical frailty characteristics (0-5) and the components of the score, and transitions between robust (0

For peer review only - http://bmjopen.bmj.com/site/about/guidelines.xhtml

For peer review only

characteristics), physical prefrailty (1-2 characteristics) and physical frailty (3+ characteristics) will be recorded.

Generic health-related quality of life will be recorded using the EQ5D-5L and SF-36 (physical and mental component summary scores) questionnaires [39,40]. Instrumental activities of daily living (ADLs) will be recorded using the Nottingham extended ADL (NEADL) score [41]. Glycosylated haemoglobin will be measured along with the homeostatic measure of insulin resistance (HOMA-IR) derived from peripheral glucose and insulin measures [42]. Advanced glycosylation end-products (AGE) presence in the skin will be measured by auto fluorescence using the AGE Reader (Diagnoptics, Gronigen, Netherlands) [43]. Blood samples will be processed and stored for later mechanistic studies, including measurement of a panel of pro-inflammatory cytokines and markers of cellular senescence and oxidative stress. Stool samples will be collected and stored for later microbiome studies. Metrics describing the conversion rate from screening to randomisation, and the retention rate of recruited participants over their four month study participation will also be collected.

Participant timeline

Participants who express interest in the trial will enter a brief pre-screening process by telephone to check provisional eligibility. Verbal consent will be sought for the pre-screen process, including access to medical records for further review of suitability for the trial. At pre-screening, participants are asked if they have a diagnosis of diabetes mellitus, are taking metformin, or have previously been intolerant of metformin. The SARC-F questionnaire will be administered [44]; this comprises five questions on physical function with a score between zero and ten. Based on previous data [45], a score of one or more will be sufficient to identify those more likely to have sarcopenia and will enable progression to the screening visit. Participants who pass the pre-screen will be given or posted the full participant information sheet (PIS), and all participants will be given at least 48 hours to consider their participation. The PIS can be found in Appendix 1.

For peer review only - http://bmjopen.bmj.com/site/about/guidelines.xhtml

Written informed consent will be obtained at the screening visit, which may take place at home, at clinic, or in a research facility. After informed consent is given, information on demographics, medical and medication history and alcohol use will be collected. Maximum handgrip and 4 metre walk speed will be measured. Blood will be taken for urea, creatinine and electrolytes (U&Es) and liver function tests (LFTs) unless results are already available from within three months prior to the screening visit. Participant eligibility to proceed to the baseline visit is confirmed after the screening visit, once screening assessment results are available.

The schedule of events is given in Table 2. Main study outcomes are measured at baseline and the four month final visit; safety visits to check renal function, lactate concentrations and adverse events take place at one, two and three months, and a telephone call takes place one week after the baseline visit to ensure that the participant has received and started the study medication.

For peer review only

Table 2. MET-PREVENT trial schedule of events

Timepoint	Pre-Screening	Screen	Baseline	1 week (+ 3 days) after randomisation	1 month (+ 1 week) after randomisation	2 months (+ 1 week) after randomisation	3 months (+ 1 week) after randomisation	4 months +/-2 weeks
**SCREENING, CONSENT and PRE-ALLOCATION ASSESSMENTS**								
Brief Study Information Sheet and Invitation Letter posted/given to patients identified via SarcNet Registry, older peoples medicine clinics, day units and rehabilitation facilities and GP practices	X							
Telephone pre-screening (verbal consent, demographics, SARC-F tool) for participants who send a positive reply slip	X							
Patient Information Sheet posted to participants who pass pre-screen	X							
Informed written consent		X						
Eligibility assessments (U&Es, LFTs, demographics, medical history, concomitant medication, adverse events, four metre walk speed, 5x sit to stand time, grip strength)		X						
Eligibility confirmation		X						
Baseline assessments pre-allocation (blood glucose and HbA1c, height and weight, bioimpedance, AGE skin fluorescence, physical performance tests (SPPB, grip strength, six min walk), quality of life questionnaires (EQ5D5L, SF-36, Nottingham EADL), frailty screening questions (activity, exhaustion).			X					
Allocation (randomisation) - following completion of assessments			X					
Dispensing/posting trial medication			X					
**BIOLOGICAL SAMPLES**								

For peer review only - http://bmjopen.bmj.com/site/about/guidelines.xhtml

Blood and stool			X					X
**INTERVENTION**								
4 month supply of Metformin hydrochloride 500mg tablets or matching placebo tablets			←					→
**FOLLOW UP**								
Confirm medication receipt, ability to open medication bottle, date of first dose				X				
Safety blood tests (U&Es, LFTs, glucose, lactate)					X	X	X	X
Adverse event assessments		X	X	X	X	X	X	X
Concomitant Medications		X	X	X	X	X	X	X
Follow up assessments (blood glucose and HbA1c, weight, bioimpedance, AGE skin fluorescence, physical performance tests (SPPB, grip strength, six min walk), quality of life questionnaires (EQ5D5L, SF-36, Nottingham EADL), frailty screening questions (activity, exhaustion).								X
Return of unused trial medication								X
Medication compliance, accountability								X

For peer review only

Sample size

Based on previous work, we assumed a minimum clinically important difference for the four metre walk speed of 0.1m/s [27]. Assuming a SD of 0.24 as seen in the English Longitudinal Study of Ageing [46], and a correlation between baseline and six month measures of 0.8, as seen in a recent trial of older people at risk of falls [47] (a similar population to those who will be enrolled in this trial), a total of 66 participants would be needed (33 per group) to give 80% power to detect this difference at a two-sided alpha=0.05 in an analysis that is adjusted for baseline values. Recruiting 80 participants will allow for 17.5% dropout (higher than observed in previous trials of spironolactone and of vitamin D in similar populations [48,49]) to give 66 participants at follow up.

Recruitment

Recruitment will take place over ten months and participants will be followed up for four months; recruitment started in September 2021. Potential participants for MET-PREVENT will be identified by four routes. The SarcNet Registry [30] contains details of participants who have had grip strength and walk speed measured previously and have consented to be recontacted with offers to participate in other studies. The NIHR BioResource Centre Newcastle registry contains details of participants who have previously given consent for re-contact with information about potential trial opportunities suitable for them (REC REF: 18/NE/0138). Participants will also be sought via Older Peoples Medicine Clinics, Day Units, assessment units and rehabilitation facilities in participating centres. Where available, existing information on handgrip strength and walk speed held in the clinical record will be used to identify potential participants. Finally, potential participants will be identified via screening of general practice (GP) lists, in collaboration with the NIHR Primary Care Research Network. Where interrogation of the electronic Frailty Index (eFI) is possible [50], invitations will be targeted to those with at least mild frailty denoted by an eFI of 0.12 or greater.

For peer review only - http://bmjopen.bmj.com/site/about/guidelines.xhtml

For peer review only

Potentially eligible participants will be sent or given a brief study information sheet, an invitation letter with a reply slip and a prepaid envelope to express interest. The reply slip will be returned to the central study team, who will pass on details to the site recruiting teams. Those who are interested will be contacted for pre-screening as described below.

Assignment of intervention

Participants will be randomised on a 1:1 basis using an interactive web-based randomisation system (Sealed Envelope Ltd). Minimisation (with a 30% random element) will ensure balance across the two arms based on the following stratification variables: sex and baseline walk speed (<=0.6 or >0.6 m/s). The allocation sequence is prepared by Sealed Envelope and is concealed from the study team. Participants are allocated a pack number at randomisation; this pack number is used by the trial pharmacy to dispense pre-labelled medication packs. Participants, the clinical team and the study team (including investigators, research nurses collecting outcomes data, senior statistician and trial manager) are blind to treatment allocation during the trial. For the IDMC, the statisticians preparing the report will be partially blind. This means that analysis will be conducted by arm, but the statisticians will not know the treatment allocation for each arm. They will become unblinded if the IDMC requests fully unblinded data. The randomisation system has functionality for emergency unblinding. This will only occur for valid medical or safety reasons where it is necessary for the treating clinician to know which treatment the participant has been receiving; the randomisation system is able to inform the treating clinician by email without unblinding other members of the study team or the participant.

Data collection and management

The trial schedule of events is presented as a flow diagram (Figure 2) and a table of trial processes (Table 2). Recruited participants will be followed up for four months from the point of randomisation. Data including the number of participants identified, approached, pre-screened and screened will be

For peer review only

collected and documented on a site screening log. Data will be handled, computerised, stored and archived in accordance with the General Data Protection Regulation (2018), and the latest Directive on GCP (2005/28/EC). Patient identifiable data will remain at each site and will not be collected as part of the trial dataset. Patient identification on data collection tools used will be through a unique sequential screening number allocated by site staff. Data will be transcribed by site staff from data collection worksheets to the trial's secure, password-limited, validated database (Sealed Envelope Ltd, London, UK). The participant trial record, including completed paper data collection tools, will be archived at site for 15 years following the end of the trial. Newcastle Clinical Trials Unit (NCTU) staff monitor trial conduct and data integrity; this is detailed in a Data Management Plan and a Monitoring Plan approved by the trial Sponsor.

Statistical analysis

A Statistical Analysis Plan will document the planned analyses and will be finalised prior to database lock at the end of the trial. For the primary outcome (four metre walk speed at four months post-randomisation), the difference between arms will be tested through fitting a linear regression model that is adjusted for baseline four metre walk speed and sex. A suitable transform of the data (for example the best fitting Box-Cox transform) will be applied to ensure the data are approximately normally distributed. The model will allow estimation of the difference between arms together with the 95% confidence interval and p-value for testing the null hypothesis of no difference. Continuous secondary endpoints will be analysed in a similar manner to the primary outcome. Binary secondary endpoints will be analysed with a logistic regression model. All models will be adjusted for the outcome under test at baseline, baseline 4 metre walk speed and sex.

The following pre-planned subgroup analyses will be performed for the primary outcome: age >75 years vs <=75 years, men vs women, baseline walk speed >0.6m/s vs <=0.6m/s. In addition, a per-protocol analysis will be performed in participants with adherence to study medication >=80%.

For peer review only - http://bmjopen.bmj.com/site/about/guidelines.xhtml

The primary analysis will be a complete case analysis. In the event the mortality rate is >5%, we will perform a sensitivity analysis by imputing the worst score possible for the primary outcome (i.e. 0 m/s) in participants who died before the four month visit. A further sensitivity analysis will be performed by imputing the primary outcome to the worst score possible in the event both mortality and withdrawal due to illness is >5%. If more than 10% of primary outcome data is missing after imputation for missing-ness due to death and withdrawal due to illness, a sensitivity analysis using multiple imputation via chained equations using the baseline covariates in the imputation model will be performed. Twenty imputed datasets will be used.

Trial oversight

A Trial Management Group (TMG), facilitated by NCTU, will convene approximately monthly throughout the duration of the trial. Members will consist of key NCTU staff, the Chief Investigator, co-applicants, trial statisticians, local site research staff and a Sponsor representative. A patient and public involvement (PPI) representative will attend TMG meetings at a frequency determined on an ongoing basis. The TMG undertakes, with the additional of external membership, the role of a Trial Steering Committee for this small, relatively low risk CTIMP study. An Independent Data Monitoring Committee (IDMC) has been appointed to provide an independent review of participant safety. The independent members comprise two clinicians and a statistician. The IDMC will meet at least annually, and report directly to the TMG.

Harms

Data from all adverse events (AEs) will be recorded on the trial database at every trial visit. Serious adverse events (SAEs) will be assessed for any relationship to the treatment intervention (causality), by a delegated, medically qualified site doctor. The following SAEs will be recorded on the database, but do not require reporting to Sponsor: any death or hospitalisation due to a new cardiovascular

For peer review only - http://bmjopen.bmj.com/site/about/guidelines.xhtml

event, new diagnosis or treatment of cancer, fall or fracture, infection, delirium, reduced mobility, exacerbation of an existing medical condition, admission for elective or planned investigation or treatment, or hospitalisation due to nausea, vomiting, constipation or diarrhoea. The above exceptions to immediate SAE reporting refer only to SAEs where the trial medication is not deemed to be causally related to the event by the local site Principal Investigator. The Chief Investigator will assess, on behalf of Sponsor, expectedness (by reference to the approved Reference Safety Information) of any serious adverse reactions (SARs).

Safety assessments will be conducted at screening and at the one, two, three and four month follow up visits. Key safety data will include serum creatinine concentrations – eGFR will be calculated to ensure adequate renal function (>30ml/min/1.73m^2), liver function tests (LFTs), blood glucose and blood lactate concentrations. Lactate levels of >4mmol/L will lead to discontinuation of trial medication.

Ethics approval

A favourable ethical opinion has been granted from the UK Health Research Authority Research Ethics Committee (North-West - Liverpool Central Research Ethics Committee; trial reference approval number 20/NW/0470). The trial has also received approval from the UK Medicines and Healthcare products Regulatory Agency (MHRA; trial reference number 2020-004023-16). The trial has been included in the National Institute for Health Research Clinical Research Network (NIHR CRN) portfolio (NIHR CLRN study ID: 47772). The trial Sponsor is the Newcastle Upon Tyne Hospitals NHS Foundation Trust, Freeman Hospital, Freeman Road, High Heaton, Newcastle upon Tyne, NE7 7DN. The trial Sponsor has delegated responsibility for trial management to NCTU, including trial design; review and approval of all localised patient-facing documentation prior to implementation at each site; collection, analysis and interpretation of data; writing of the protocol publication and final clinical report

For peer review only - http://bmjopen.bmj.com/site/about/guidelines.xhtml

manuscripts. This protocol manuscript is based on version 7.0 of the trial protocol (dated 13th December 2021)

Consent

Informed, written consent will be sought at the screening visit, prior to conducting any trial procedures, including screening assessments. Where the screening visit takes place in the participant's home, the informed consent discussion with a delegated local investigator may take place in person or remotely by telephone, teleconference or videoconference. A delegated research nurse will be present in the participant's home to facilitate this, and to countersign the consent form. The investigator will complete, date and sign a consent interview proforma to provide a comprehensive account of the remote consent interview.

Access to data

Access to the full blinded dataset will be limited to the TMG and to authors of the trial publication. At the end of the trial, a de-identified dataset will be prepared and stored by Newcastle University. Requests for data sharing with study teams outside Newcastle University or the study Sponsor, including international requests, will be considered by a Data Access Committee with representation from the Funder, Sponsor and the trial Chief Investigator.

Ancillary and post-trial care

No provision for continuation of trial medication will be made by the trial team or Sponsor. Metformin is not licensed for the indication under study in this trial; any off-licence use of metformin after the end of the trial would be the responsibility of the participant's usual primary or secondary care clinical team. Participants and their GP will be informed by letter of which treatment they took after all participants have completed their final visit and the database is locked.

For peer review only - http://bmjopen.bmj.com/site/about/guidelines.xhtml

Dissemination policy

A final report of the trial will be provided to the Sponsor, Research Ethics Committee and the trial Funder within one year of the end of the study. The trial results will be uploaded to the European Union Drug Regulating Authorities Clinical Trials (EudraCT) database, as per the European Commission's guidelines on posting and publication of result-related information within 12 months. The trial is registered on the ISRCTN trial database and trial results will be made publicly available on the ISRCTN trial registry within 12 months of the end of the trial, defined as Last Patient Last Visit date. The final clinical trial report will be used for publication and presentation at scientific meetings. Summaries of results will also be made available to investigators for dissemination within their clinical areas and to the wider public, and a summary of results will be sent to all participants along with their treatment allocation. If feasible within pandemic meeting restrictions, we will hold a dissemination event for participants and their families to present and discuss the study results.

Patient and Public Involvement

A patient panel (convened by VOICE Global) consisting of older people with lived experience of muscle weakness was involved in the design of the trial protocol, selection of outcome measures and reviewed the participant information sheets. A lay member (SC) sits on the Trial Management Group and is involved in the overall management of the trial and in dissemination plans for the results.

Discussion

Randomised controlled trials of treatments for sarcopenia and physical frailty are challenging to perform; previous trials have struggled to recruit their anticipated target numbers within the allocated time [51]. This is in part because sarcopenia (and to a lesser extent physical frailty) are not diagnoses commonly made or recorded in clinical practice and thus identifying people with these conditions is not straightforward. In addition, people with sarcopenia usually have multimorbidity [52] and by

For peer review only - http://bmjopen.bmj.com/site/about/guidelines.xhtml

definition have components of the physical frailty syndrome. As a result, they may find it difficult to take part in clinical trials unless these trials are designed to facilitate their inclusion and retention.

We have sought to broaden the range of recruitment strategies employed to identify patients with sarcopenia and physical frailty. MET-PREVENT is one of the first sarcopenia trials to use a sarcopenia registry [30] to facilitate recruitment, and we also build on recent work deploying measures of muscle strength and walk speed into routine clinical practice to facilitate recruitment. Although recruitment through primary care lacks specificity for identifying those with sarcopenia or physical frailty, the use of the electronic frailty index can improve the specificity of searches, and large numbers of potentially eligible participants can be reached via this route. We have aimed to minimise the burden of study visits in MET-PREVENT, both in terms of visit duration and tasks to complete during each visit, but also by providing a flexible approach to the venue in which study visits are undertaken. Many patients with sarcopenia or physical frailty find it difficult to travel to study centres and some are unable to leave their home at all. Our flexible approach to study visits with many of them taking place in the homes of participants enables this group of patients who would normally be excluded from participation to take part in research.

Although the trial is powered to detect the minimum clinically important difference in four metre walk speed, it is not powered to detect clinically important differences for some of the secondary outcomes. However, the trial seeks to provide proof-of-concept for a larger, multicentre trial that would be powered to detect differences in these outcomes, including transitions from physical prefrailty to frailty. The trial population excludes some groups for safety reasons (for instance those with chronic kidney disease) which limits the generalisability of the results. Muscle biopsies are not being performed, thus direct information on changes in skeletal muscle structure and function will not be obtainable from this trial.

For peer review only - http://bmjopen.bmj.com/site/about/guidelines.xhtml

The collection of blood and stool samples for future mechanistic analyses will maximise the scientific value of the trial but also look to identify those groups that may be more likely to benefit from metformin as an intervention in a future large trial. In addition, the mechanistic analyses (which will be performed after the main trial is complete) will identify which mechanistic pathways are most important in mediating any benefit of metformin and in doing so will help us identify pathways and featured targets for other interventions to prevent or treat sarcopenia and physical frailty. We believe that this study design provides a template for other phase II intervention trials for sarcopenia and physical frailty; this template should both accelerate progress in translational research and also enable easier comparison of results across different trials [53].

Author contributions

MDW is the trial Chief Investigator and senior author. MDW, AAS, AC, HH, CMcD, CS, and TvZ led the funding application and protocol development. JW was the trial Senior Statistical Advisor during the funding bid and advised on trial design. SH is the Trial Statistician. NW is the Senior Statistician and leads on the statistical analysis plan. PB is the Sponsor Pharmacy representative, and SC provided patient and public input. AJS, KJR, LM, LS and LR provided trial management and trial monitoring. KJR and MDW drafted the manuscript for this publication. All authors contributed to protocol development and critical revision of the manuscript.

Funding

This work was supported by the NIHR Newcastle Biomedical Research Centre, reference NU001533

Competing Interests

The authors declare that they have no competing interests.

For peer review only - http://bmjopen.bmj.com/site/about/guidelines.xhtml

Acknowledgements

The authors would like to thank the members of the MET-PREVENT Independent Data Monitoring Committee (DMC) for their continuing valuable expertise:

- DMC Chair - Dr Terry Quinn (Senior Lecturer and Honorary Consultant, University of Glasgow)
- DMC Statistician – Dr Lorna Aucott (Senior Statistician, Health Services Research Unit, University of Aberdeen)
- DMC Clinician - Dr Victoria Haunton (Honorary Senior Lecturer / Consultant Geriatrician, University of Plymouth/University Hospitals Plymouth NHS Trust).

References

[1] Cruz-Jentoft AJ, Sayer AA. Sarcopenia. *Lancet* 2019;393:2636-46.

[2] Lunt E, Ong T, Gordon AL, et al. The clinical usefulness of muscle mass and strength measures in older people: a systematic review. *Age Ageing* 2021;50:88-95.

[3] Liu P, Hao Q, Hai S, et al. Sarcopenia as a predictor of all-cause mortality among community-dwelling older people: A systematic review and meta-analysis. *Maturitas* 2017;103:16-22.

[4] Yeung SSY, Reijnierse EM, Pham VK, et al. Sarcopenia and its association with falls and fractures in older adults: A systematic review and meta-analysis. *J Cachexia Sarcopenia Muscle* 2019;10:485-500.

[5] Clegg A, Young J, Iliffe S, et al. Frailty in elderly people. *Lancet* 2013;381:752–62.

[6] Fried LP, Tangen CM, Walston J, et al. Frailty in older adults: evidence for a phenotype. *J Gerontol A Biol Sci Med Sci* 2001;56:M146-56.

[7] Roberts H, Khee TS, Philp I. Setting priorities for measures of performance for geriatric medical services. *Age Ageing* 1994;23:154-7.

For peer review only - http://bmjopen.bmj.com/site/about/guidelines.xhtml

[8] Cruz-Jentoft AJ, Landi F, Schneider SM, et al. Prevalence of and interventions for sarcopenia in ageing adults: a systematic review. Report of the International Sarcopenia Initiative (EWGSOP and IWGS). *Age Ageing* 2014;43:748-59.

[9] Moore SA, Hrisos N, Errington L, et al. Exercise as a treatment for sarcopenia: An umbrella review of systematic review evidence. *Physiotherapy* 2019;107:189-201.

[10] Apóstolo J, Cooke R, Bobrowicz-Campos E, et al. Effectiveness of interventions to prevent pre-frailty and frailty progression in older adults:a systematic review. *JBI Database System Rev Implement Rep* 2018;16:140–232.

[11] Morley JE. Pharmacologic Options for the Treatment of Sarcopenia. *Calcif Tissue Int* 2016;98:319-33.

[12] Murphy MP. How mitochondria produce reactive oxygen species. *Biochem J* 2009;417:1-13.

[13] Kane DA, Anderson EJ, Price JW, et al. Metformin selectively attenuates mitochondrial H2O2 emission without affecting respiratory capacity in skeletal muscle of obese rats. *Free Radic Biol Med* 2010;49:1082–7.

[14] Hardie DG. AMPK: positive and negative regulation, and its role in whole-body energy homeostasis. *Curr Opin Cell Biol* 2015;33:1–7.

[15] Saxton RA, Sabatini DM. mTOR Signaling in Growth, Metabolism, and Disease. *Cell* 2017;168:960-76.

[16] Castets P, Lin S, Rion N, et al. Sustained activation of mTORC1 in skeletal muscle inhibits constitutive and starvation-induced autophagy and causes a severe, late-onset myopathy. *Cell Metabol* 2013; 17:731–44.

[17] Kim SA, Choi HC. Metformin inhibits inflammatory response via AMPK-PTEN pathway in vascular smooth muscle cells. *Biochem Biophys Res Comm* 2012;425:866-72.

[18] Gu J, Ye S, Wang S, et al. Metformin inhibits nuclear factor-kappaB activation and inflammatory cytokines expression induced by high glucose via adenosine monophosphate-activated protein kinase activation in rat glomerular mesangial cells in vitro. *Chin Med J* 2014;127:1755-60.

For peer review only - http://bmjopen.bmj.com/site/about/guidelines.xhtml

[19] Andrews M, Soto N, Arredondo M. Effect of metformin on the expression of tumor necrosis factor-α, Toll like receptors 2/4 and C reactive protein in obese type-2 diabetic patients. *Revista Medica De Chile* 2012;140:1377-82.

[20] Prattichizzo F, Giuliani A, Mensà E, et al. Pleiotropic effects of metformin: Shaping the microbiome to manage type 2 diabetes and postpone ageing. *Ageing Res Rev* 2018;48:87-98.

[21] Heintz C, Mair W. You are what you host: microbiome modulation of the aging process. *Cell* 2014;156:408-11.

[22] Jackson MA, Verdi S, Maxan ME, et al. Gut microbiota associations with common diseases and prescription medications in a population-based cohort. *Nat Commun* 2018;9:2655.

[23] Short S, Fielder E, Miwa S, et al. Senolytics and senostatics as adjuvant tumour therapy. *EBioMedicine* 2019;41:683-92.

[24] Xu H, Aldrich MC, Chen Q, et al. Validating drug repurposing signals using electronic health records: a case study of metformin associated with reduced cancer mortality. *Am Med Inform Assoc* 2015;22:179–91

[25] Wang CP, Lorenzo C, Habib SL, et al. Differential effects of metformin on age related comorbidities in older men with type 2 diabetes. *J Diabet Complic* 2017;31:679-86.

[26] Sumantri S, Setiati S, Purnamasari D, et al. Relationship between metformin and frailty syndrome in elderly people with type 2 diabetes. *Acta Med Indones* 2014;46:183–8.

[27] Laksmi PW, Setiati S, Tamin TZ, et al. Effect of Metformin on Handgrip Strength, Gait Speed, Myostatin Serum Level, and Health-related Quality of Life: A Double Blind Randomized Controlled Trial among Non-diabetic Pre-frail Elderly Patients. *Acta Med Indones* 2017;49:118-27.

[28] Perera S, Mody SH, Woodman RC, et al. Meaningful change and responsiveness in common physical performance measures in older adults. *J Am Geriatr Soc* 2006;54:743-9.

[29] Walton RG, Dungan CM, Long DE, et al. Metformin blunts muscle hypertrophy in response to progressive resistance exercise training in older adults: A randomized, double-blind, placebo-controlled, multicenter trial: The MASTERS trial. *Aging Cell* 2019;18:e13039.

For peer review only

[30] Witham MD, Heslop P, Dodds RM, et al. Developing a UK sarcopenia registry: recruitment and baseline characteristics of the SarcNet pilot. *Age Ageing* 2021;50:1762-9.

[31] Cruz-Jentoft AJ, Bahat G, Bauer J, et al. Sarcopenia: revised European consensus on definition and diagnosis. *Age Ageing* 2019;48:16-31.

[32] Studenski S, Perera S, Patel K, et al. Gait speed and survival in older adults. *JAMA* 2011;305:50-8.

[33] Hoogendijk EO, Rijnhart JJM, Skoog J, et al. Gait speed as predictor of transition into cognitive impairment: Findings from three longitudinal studies on aging. *Exp Gerontol* 2020;129:110783.

[34] Abellan van Kan G, Rolland Y, Andrieu S, et al. Gait speed at usual pace as a predictor of adverse outcomes in community-dwelling older people an International Academy on Nutrition and Aging (IANA) Task Force. *J Nutr Health Aging* 2009;13:881-9.

[35] Roberts HC, Denison HJ, Martin HJ, et al. A review of the measurement of grip strength in clinical and epidemiological studies: towards a standardised approach. *Age Ageing* 2011;40:423-9.

[36] Guyatt GH, Sullivan MJ, Thompson PJ, et al. The 6-minute walk: a new measure of exercise capacity in patients with chronic heart failure. *Can Med Assoc J* 1985;132:919-23.

[37] Guralnik JM, Simonsick EM, Ferrucci L, et al. A short physical performance battery assessing lower extremity function: association with self-reported disability and prediction of mortality and nursing home admission. *J Gerontol* 1994;49:M85-94.

[38] Sergi G, De Rui M, Veronese N, et al. Assessing appendicular skeletal muscle mass with bioelectrical impedance analysis in free-living Caucasian older adults. *Clin Nutr* 2015;34:667-73.

[39] Herdman M, Gudex C, Lloyd A, et al. Development and preliminary testing of the new five-level version of EQ-5D (EQ-5D-5L). *Qual Life Res* 2011;20:1727–36.

[40] Ware JE, Kosinski M, Bjorner JB, et al. User's Manual for the SF-36v2TM Health Survey. 2nd edition. Lincoln (RI): QualityMetric; 2007.

[41] Nouri FM, Lincoln NB. An extended activities of daily living scale for stroke patients. *Clin Rehabilitation* 1987;1:301-5.

For peer review only - http://bmjopen.bmj.com/site/about/guidelines.xhtml

[42] Matthews DR, Hosker JP, Rudenski AS, et al. Homeostasis model assessment: insulin resistance and beta-cell function from fasting plasma glucose and insulin concentrations in man. *Diabetologia* 1985;28:412-9.

[43] Drenth H, Zuidema SU, Krijnen WP, et al. Advanced Glycation End Products Are Associated With Physical Activity and Physical Functioning in the Older Population. *J Gerontol A Biol Sci Med Sci* 2018;73:1545-51.

[44] Malmstrom TK, Morley JE. SARC-F: a simple questionnaire to rapidly diagnose sarcopenia. *J Am Med Dir Assoc* 2013;14:531-2.

[45] Dodds RM, Murray JC, Robinson SM, et al. The identification of probable sarcopenia in early old age based on the SARC-F tool and clinical suspicion: findings from the 1946 British birth cohort. *Eur Geriatr Med* 2020;11:433-441.

[46] Witham MD, Wason J, Dodds R, et al. Developing a composite outcome measure for frailty prevention trials - rationale, derivation and sample size comparison with other candidate measures. *BMC Geriatr* 2020;20:113.

[47] Witham MD, Price RJG, Band MM, et al. Effect of Vitamin K2 on Postural Sway in Older People Who Fall: A Randomized Controlled Trial. *J Am Geriatr Soc* 2019;67:2102-7.

[48] Burton LA, Sumukadas D, Witham MD, et al. Effect of spironolactone on physical performance in older people with self-reported physical disability. *Am J Med* 2013;126:590-7.

[49] Witham MD, Price RJ, Struthers AD, et al. Cholecalciferol treatment to reduce blood pressure in older patients with isolated systolic hypertension: the VitDISH randomized controlled trial. *JAMA Intern Med* 2013;173:1672-9.

[50] Clegg A, Bates C, Young J, et al. Development and validation of an electronic frailty index using routine primary care electronic health record data. *Age Ageing* 2016;45:353-60.

[51] Witham MD, Achison M, Aspray TJ, et al. Recruitment strategies for sarcopenia trials: lessons from the LACE randomized controlled trial. *JCSM Rapid Comms* 2021;4:93-102.

For peer review only - http://bmjopen.bmj.com/site/about/guidelines.xhtml

[52] Dodds RM, Granic A, Robinson SM, et al. Sarcopenia, long-term conditions, and multimorbidity: findings from UK Biobank participants. *J Cachexia Sarcopenia Muscle* 2020;11:62-8.

[53] Witham MD. Bridging the gap between the laboratory and the clinic for patients with sarcopenia. *Biogerontology* 2019;20:241-8.

For peer review only

For peer review only - http://bmjopen.bmj.com/site/about/guidelines.xhtml

Figure 1. Potential mechanisms of action through which metformin could improve skeletal muscle function

AMP Kinase: Adenosine MonoPhosphate activated protein Kinase

mTOR: mammalian Target Of Rapamycin

Figure 2. MET-PREVENT trial flow diagram

For peer review only

For peer review only - http://bmjopen.bmj.com/site/about/guidelines.xhtml

For peer review only

For peer review only - http://bmjopen.bmj.com/site/about/guidelines.xhtml

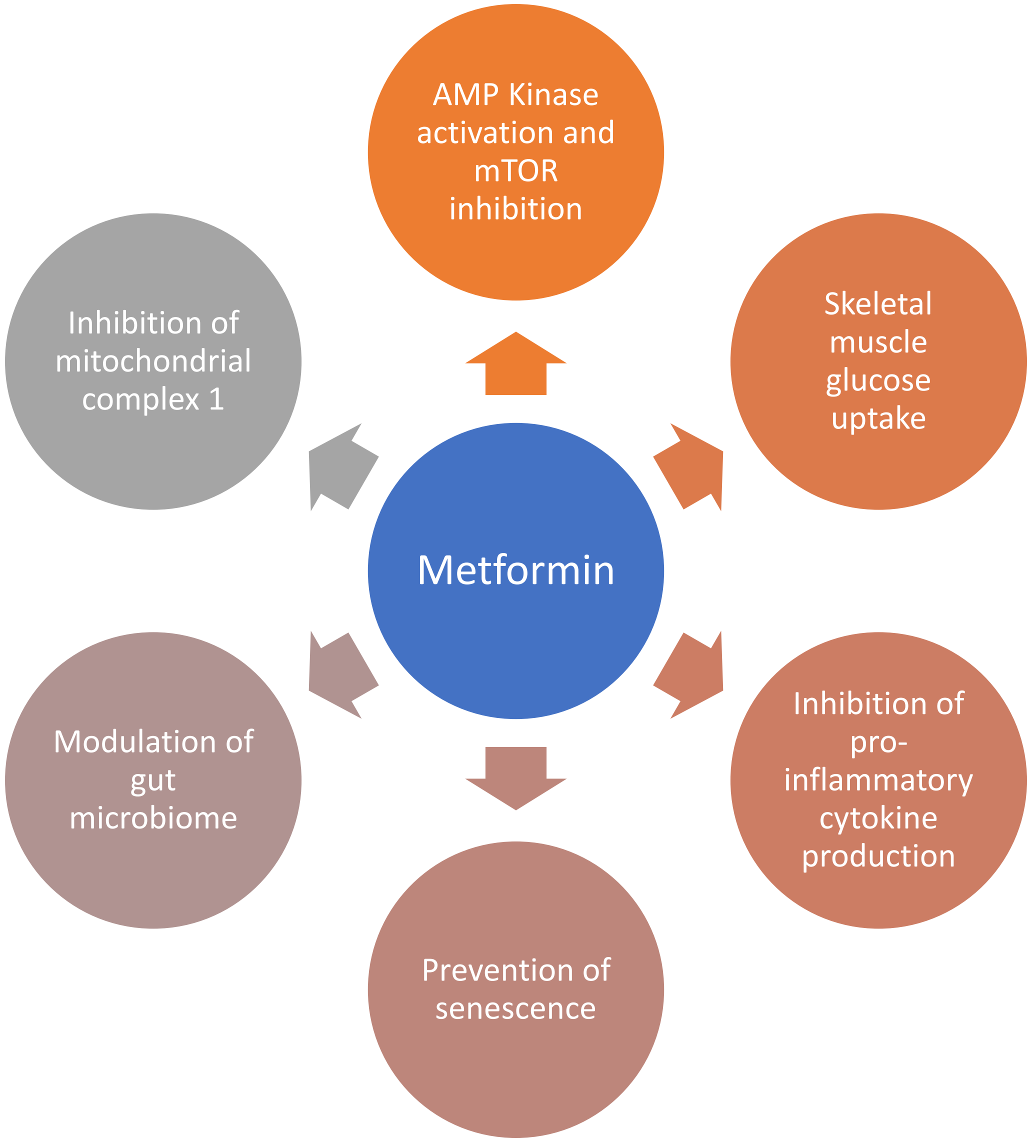
AMP Kinase activation and mTOR inhibition
Skeletal muscle glucose uptake
Inhibition of pro-inflammatory cytokine production
Prevention of senescence
Modulation of gut microbiome
Inhibition of mitochondrial complex 1
Metformin

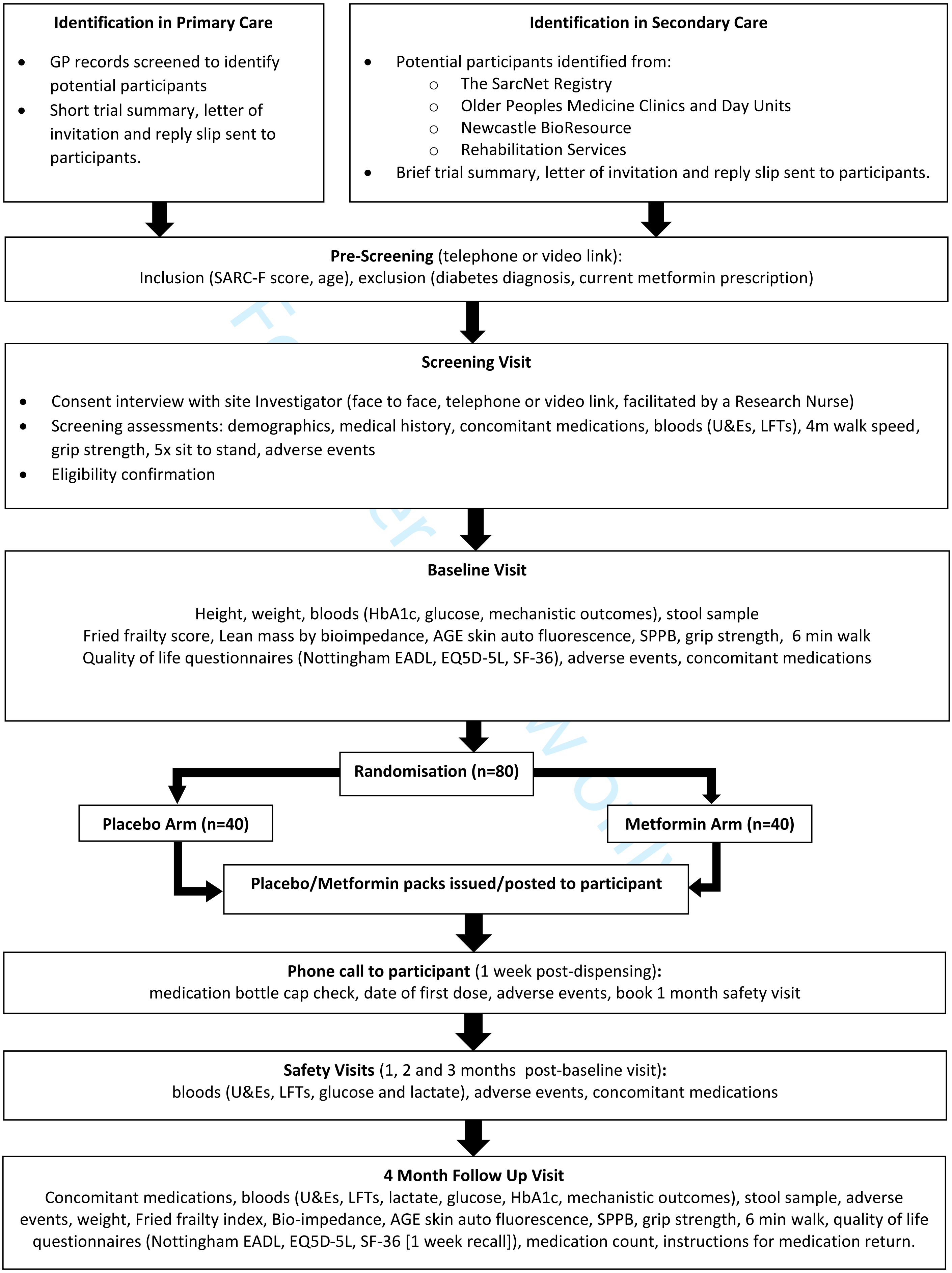
Identification in Primary Care
GP records screened to identify potential participants
Short trial summary, letter of invitation and reply slip sent to participants.
Identification in Secondary Care
Potential participants identified from:
The SarcNet Registry
Older Peoples Medicine Clinics and Day Units
Newcastle BioResource
Rehabilitation Services
Brief trial summary, letter of invitation and reply slip sent to participants.
Pre-Screening (telephone or video link):
Inclusion (SARC-F score, age), exclusion (diabetes diagnosis, current metformin prescription)
Screening Visit
Consent interview with site Investigator (face to face, telephone or video link, facilitated by a Research Nurse)
Screening assessments: demographics, medical history, concomitant medications, bloods (U&Es, LFTs), 4m walk speed, grip strength, 5x sit to stand, adverse events
Eligibility confirmation
Baseline Visit
Height, weight, bloods (HbA1c, glucose, mechanistic outcomes), stool sample
Fried frailty score, Lean mass by bioimpedance, AGE skin auto fluorescence, SPPB, grip strength, 6 min walk
Quality of life questionnaires (Nottingham EADL, EQ5D-5L, SF-36), adverse events, concomitant medications
Randomisation (n=80)
Placebo Arm (n=40)
Metformin Arm (n=40)
Placebo/Metformin packs issued/posted to participant
Phone call to participant (1 week post-dispensing):
medication bottle cap check, date of first dose, adverse events, book 1 month safety visit
Safety Visits (1, 2 and 3 months post-baseline visit):
bloods (U&Es, LFTs, glucose and lactate), adverse events, concomitant medications
4 Month Follow Up Visit
Concomitant medications, bloods (U&Es, LFTs, lactate, glucose, HbA1c, mechanistic outcomes), stool sample, adverse events, weight, Fried frailty index, Bio-impedance, AGE skin auto fluorescence, SPPB, grip strength, 6 min walk, quality of life questionnaires (Nottingham EADL, EQ5D-5L, SF-36 [1 week recall]), medication count, instructions for medication return.

Newcastle University

<local NHS Trust logo>

MET-PREVENT

Metformin to prevent progression to frailty for older people – a randomised controlled proof of concept trial

Introduction

Thank you for considering taking part in the MET-PREVENT study. Before you decide whether to take part, please take time to read this information about the study carefully. Feel free to discuss it with your family, friends, carers or your GP if you wish to do so. If anything is unclear, or you need more information, please ask. You will find our contact details at the back of the information. This leaflet is yours to keep.

SUMMARY of the MET-PREVENT STUDY

Purpose of the study

- We would like to find out whether a medicine called metformin (used to treat type 2 diabetes) can help people over the age of 65 with muscle weakness but without diabetes to become stronger and improve their daily activities.

Will I definitely get metformin?

- No. To ensure that the study is a fair test, a computer will randomly allocate you to either metformin or the placebo (dummy) tablets. There is an equal chance that you will receive metformin or the dummy tablets. Both types of tablet are identical. Neither you nor the doctor, nurse or study team will know which treatment you are receiving.

What are the side effects of the medicine?

- The medicine may cause nausea (feeling sick) or loose bowels in some people. Most people do not get either

For peer review only - http://bmjopen.bmj.com/site/about/guidelines.xhtml

<local NHS Trust logo>

of these effects. These side effects usually disappear after a few days or a couple of weeks.

If I take part, what will I have to do?

- All of the study visits may take place in your own home, at a research centre, or at a hospital clinic.
- You will be asked to attend a screening visit. The doctor will discuss the study with you and you can ask questions. We will ask to take a small blood sample to see if it is safe for you to take part.
- If you are suitable, we will ask you to take part in another 5 visits (3 of these are short safety visits).
- You will be asked to take a tablet 3 times a day for 4 months.
- We will ask you to give small blood samples at every visit (6 teaspoons), and a small stool (poo) sample at 2 visits (you can collect this in advance of the visit).
- At 2 visits we will ask you to do some simple tests that involve balance, walking a short distance, getting up from a chair and gripping a measuring device with your hand. You will also be asked to complete 3 questionnaires that ask about your daily activities.

Do I have to visit the hospital to take part?

- Not if you don't want to. We can visit you in your own home if you wish. A member of the local clinical team will bring all the equipment.

Do I have to take part?

- No – it is entirely your choice whether you wish to take part or not. Your care will not be affected in any way.

If you are still interested in taking part in the MET-PREVENT study, please read on for more information.

For peer review only - http://bmjopen.bmj.com/site/about/guidelines.xhtml

<local NHS Trust logo>

Figure 1. Summary of the MET-PREVENT study visits

SCREENING VISIT – 1 to 2 HOURS

- Written informed consent with doctor
- Medical history, other medicines
- Physical tests (short walk, sit to stand and handgrip strength)
- Blood tests (2 teaspoons)
- After visit - eligibility for the study confirmed

BASELINE VISIT – 1 to 2 HOURS

- Have stool sample ready
- Height and weight
- Physical tests (walking, balance, sit to stand and handgrip strength)
- Light beam skin test and muscle measurement
- 3 questionnaires
- Blood samples (6 teaspoons)

1, 2 and 3 MONTH FOLLOW UPS – about 30 minutes

- Medicines review
- Review of any episodes of ill health
- Safety blood sample (2 teaspoons)

4 MONTH FOLLOW UP (FINAL) VISIT – 1 to 2 HOURS

- Have stool sample ready
- Height and weight
- Physical tests (walking, balance, sit to stand and handgrip strength)
- Light beam skin test and muscle measurement
- 3 questionnaires
- Blood samples (6 teaspoons)
- Review of other medicines
- Return unused study medicine

For peer review only - http://bmjopen.bmj.com/site/about/guidelines.xhtml

<local NHS Trust logo>

What is the purpose of the MET-PREVENT study?

Many of us lose muscle size and strength as we get older – this is called sarcopenia. People with weaker muscles are more likely to fall over and may start to have problems carrying out normal daily activities. They may also take longer to recover from other illnesses. Falling, struggling with daily living and taking a long time to get better from being unwell is a condition called frailty.

The best way to keep up your muscle strength is to do strengthening exercises, but not everyone wants to, or is able to do these.

Metformin is a medicine that is already safely used in older people to treat type 2 diabetes. Recent research suggests that metformin might help to improve muscle strength, even in people who do not have diabetes. To know if this is correct, we need to test metformin in a clinical trial. The MET-PREVENT study will test if metformin can improve muscle strength in older people who have signs of muscle weakness.

What are the possible side effects of the study medicine?

As with any medicine, the medicine used in this study (**metformin**) may cause side effects in some people. Doctors in England write millions of prescriptions for metformin every year, and the side effects are well understood.

In some people, metformin can cause nausea (occasionally vomiting), loss of appetite, tummy ache or loose bowel motions. However, we are using a low dose of metformin to reduce the chance of any of these side effects. These effects usually occur during the first few weeks of treatment and then ease in most people. To help prevent them, we will ask you to take your study medicine in 3 daily doses during or just after meals.

If you become unwell for some other reason, metformin can cause a build-up of acid in the blood. This is very rare. Occasionally, metformin may also cause a skin rash or irritate the liver. We will do regular blood tests to watch out for these problems. If you do become ill for some other reason, your doctors may stop the study medicine for a few days until you get better. This helps to prevent acid build-up in the blood. **More detail on these side effects is provided in the Supporting Information on page 10.**

For peer review only - http://bmjopen.bmj.com/site/about/guidelines.xhtml

Newcastle University

<local NHS Trust logo>

We will issue you with a safety card that will have your medicine dose printed on it. The safety card will also have the contact details of the study team at your local hospital. If you see other healthcare professionals, you should show them the safety card to let them know that you are taking part in a clinical study.

The 4 month follow-up visit is the last study visit. For the 4 weeks (28 days) following on from this final visit, we would like to know if you have been unwell. We will ask you to contact your study doctor or a member of their team to tell them about this. You can contact your study doctor and team on the contact details given on the last page of this information sheet.

If you are unsure whether the study doctor needs to know about an illness or not, please get in touch with your study doctor or a member of their team to check.

What would I have to do if I took part?

We will ask you to attend 6 visits, either at hospital or in your own home:

- Screening visit
- Baseline visit
- 1, 2 and 3 month safety follow-up visits
- 4 month follow-up visit

These visits are extra, and not part of your usual NHS care. There is a diagram (Figure 1) on page <insert page number>that shows the details of the trial visits.

Screening Visit

At the screening visit the doctor will discuss the study with you, and you will be able to ask questions. If this visit takes place in your home, a member of the research clinical team (usually a research nurse) will use a phone to let you talk to the doctor. This may be over a videolink. We will then ask if you are happy to give written consent to take part in the study. We will ask you some questions about yourself and your health. We will also ask to take a small

For peer review only - http://bmjopen.bmj.com/site/about/guidelines.xhtml

<local NHS Trust logo>

blood sample (2 teaspoons) to make sure that it is safe for you to take part in this study. We will ask you to walk a short distance (4 metres), stand up and sit down from a chair five times, and test your handgrip strength.

We will ask you if you are able to open child-resistant caps on medicine bottles

When we have the results of the blood test, we will call you and arrange the next visit.

Baseline Visit

We will ask you to take part in a number of assessments before you start the study medicine. We will ask that you have a small stool (poo) sample ready in advance. We will ask you to do the following:

- We will measure your weight and height.
- Walk a short distance (4 metres), with your usual walking aids.
- Walk for 6 minutes, with your usual walking aids.
- To test your balance, we will ask you to stand beside the researcher for a short time (no more than 30 seconds).
- Stand up from a chair.
- Use a handgrip on both hands.
- Put sticky pads on your hand and foot connected to a device that measures the amount of muscle in your body.
- Measure the response of your skin to shining a light beam on it. This device tells us about different substances inside your skin without needing to take a skin sample).
- Complete 3 questionnaires that ask how you feel about your health and your activities of daily living (such as washing and cooking).
- Have a small amount of blood taken (6 teaspoons).
- Ask about other medicine that you may be taking.

The study medicine will then be issued to you. If you are attending hospital, there will be a short wait for this. If the baseline visit takes place in your own home, or if the wait at hospital may be too long, the study medicine will be posted to you via Royal Mail or delivered by local courier. A member of the study team will phone you to check that you have received the medicine. They will also check that you can open the bottle, and have taken the first dose.

For peer review only - http://bmjopen.bmj.com/site/about/guidelines.xhtml

<local NHS Trust logo>

1, 2 and 3 month follow up visits

These visits are to check that it is safe for you to continue in the study. To do this, we will ask you for a small blood sample (2 teaspoons) at each visit. We will ask you for a list of all of your other medicines that you take. We will also collect information about all episodes of ill-health that you have experienced in the past month.

4 month follow up visit

This the last study visit. We will ask you to have a stool (poo) sample ready. We will ask you to repeat all of the tests that you did at the baseline visit, including the blood samples. We will also collect any unused trial medicine.

What are the benefits and disadvantages of taking part in this study?

We cannot promise the study will help you directly. However, the information we collect from this study may help to improve the treatment for people with muscle weakness. If you want to find out more about taking part in research studies, you can visit the NHS Choices website www.nhs.uk.

As with any medicine, metformin can cause side effects in some people. We are using a low dose to reduce the chance of this happening. If you do get a side effect from the study medicine, your doctor can stop your medicine. The side effects should disappear rapidly. In case of an emergency, the doctors can find out which treatment you are taking (metformin or placebo) if they need to.

Is it safe for me to take part in this study during COVID-19?

The study will only take place if your hospital says that it is safe to do so. You can take part in the study in your own home if you wish to avoid visiting the hospital. The study team will follow all of the hospital COVID-19 policies and procedures when they visit you, including wearing masks, gloves and visors. These will be explained to you when your appointments are arranged.

For peer review only - http://bmjopen.bmj.com/site/about/guidelines.xhtml

<local NHS Trust logo>

What happens to my blood and stool samples?

At the baseline and 4 month visits, giving the stool (poo) sample and the large blood sample (4 teaspoons) is optional. We still need a small blood sample for safety tests (2 teaspoons). The samples will be stored in a licenced Newcastle University Biobank at the end of the study. **Your consent to store these additional samples in the biobank is optional.** Your biobank samples may be used in further research linked to this study. They may also be used by other researchers in different research studies. Your samples can only be identified by using your unique study identity number. Researchers who use your samples will not know who you are.

Other researchers may want to use parts of your biobank sample in animal or commercial (paid) research. You can give samples to the biobank, but opt out of them being used for animal or paid research.

Pregnancy

We know that female participants over the age of 65 years cannot become pregnant.

However, if you are a male participant, we will ask you to inform us if your female partner becomes pregnant, or is breast-feeding an infant. We will ask for your consent to do this. We will also ask your partner to sign a consent form. This will allow the study team to collect safety information about their pregnancy and their baby.

What happens at the end of the study?

At the end of the study (4 months) you will stop taking the study medicine. You will continue to receive standard care like any other patient with your condition under the care of your GP and/or hospital doctor.

For peer review only - http://bmjopen.bmj.com/site/about/guidelines.xhtml

Newcastle University

<local NHS Trust logo>

When everyone has completed the study, we will analyse the results and we will tell you what the results are. We can either invite you to a study event to or send you a written newsletter – whichever you prefer.

We would like to follow your progress with your health over the next 5 years. We will do this by looking at your medical records, and we will ask your permission to do this. You do not have to do anything else.

Who do I contact for further information?

We will be happy to answer any questions you, your family or your carers may have about any aspect of this clinical study. Please call the number at the end of this booklet to speak to the Research Nurse at your local hospital.

For peer review only

For peer review only - http://bmjopen.bmj.com/site/about/guidelines.xhtml

<local NHS Trust logo>

SUPPORTING INFORMATION

Metformin side effects

Very common – nausea, vomiting, diarrhoea, abdominal pain and loss of appetite (this usually stops in a few weeks in most people).

Common – taste disturbance.

Very rare – lactic acid build up in the blood

Very rare - abnormalities in liver function tests or hepatitis (goes away after metformin is stopped).

Very rare – skin redness, itching, hives.

Very rare – decreased vitamin B12 absorption (only seen with long term use of metformin or in patients who already have a vitamin B12 absorption problem called megaloblastic anaemia)

Why was I contacted?

We have contacted you because you are aged 65 or over and our measurements or questionnaire results suggest that your muscles are not as strong as they used to be. This trial is testing a medicine (metformin) to see if we can prevent further weakening of muscles in people like you.

Do I have to take part?

No - it is up to you to decide whether or not to take part in this study. You do not have to take part. If you choose not to, you will continue to get the standard care arranged by your doctor.

If you agree to take part, we will ask you to sign a consent form. We will give you a copy of your signed consent form which is yours to keep. You can still change your mind and withdraw at any time without having to give a reason. If you decide not to take part, or withdraw from the study later on, your current or future medical care will not be affected in any way.

What does giving consent mean for me?

By signing a consent form, this means that you fully understand what taking part in the study means for you. That's why it is really important that you take as much time as you want to read this information sheet. Feel free to discuss

For peer review only - http://bmjopen.bmj.com/site/about/guidelines.xhtml

<local NHS Trust logo>

the study with your family, friends or any healthcare professional. At the screening visit, you will discuss the study with a study doctor, who will answer all of your questions.

Will I know what treatment I am on?

No. A computer will decide whether you will be given either the active medicine (metformin) or the placebo (dummy) tablets for 4 months. There is an equal chance that you will be given metformin or placebo tablets. The metformin and placebo tablets look identical. This means that neither you, the researchers, nor your medical teams will know what tablets you are taking. If no-one knows what tablets you are taking, no-one can influence the results of the study.

At the end of the study, when everyone has completed their visits, you may find out which tablets you were taking. We will also write to your GP to tell them what tablets you were taking. We will ask your permission to do this.

Who has checked the scientific quality of the MET-PREVENT study?

Independent experts at Newcastle University have checked the quality of the science used to plan this study. The study has also been checked and approved by a research ethics committee (North West - Liverpool Central Research Ethics Committee). The ethics committee ensures that when you take part in the study, your rights and wellbeing will be protected. The study has also been checked by the government Medicines and Healthcare products Regulatory Agency (MHRA). The MHRA are responsible for approving all studies involving medicines. The Health Research Authority gives final overall approval for the study. The Newcastle upon Tyne Hospitals NHS Foundation Trust is the study Sponsor, which means that they have overall responsibility for the study. The Sponsor has carefully checked all of the study documentation. The Sponsor has also assessed the risks of this study. This is to ensure that we are not doing anything harmful to you during the study and that your information is collected safely and stored securely.

What happens if relevant new information becomes available?

For peer review only - http://bmjopen.bmj.com/site/about/guidelines.xhtml

<local NHS Trust logo>

During the course of the study, if new information on the risks or benefits of metformin becomes available, we will let you know at your next study visit. If this new information requires urgent action, we will contact you before your next visit. If necessary, we will then discuss whether you should or would like to withdraw from the study.

Will you tell my GP that I'm taking part in a clinical study?

With your permission, we will write to your GP to tell them that you are taking part in this study. Your hospital medical record will also show that you are taking part in a clinical study. It is important for your safety that that your GP practice and hospital medical records show that you took part in a clinical study. If we discover a new health problem during the study, we will tell you. With your permission, we will also tell your GP. Any blood test results from taking part in this study will also be added to your medical records. Your GP will be asked to let the study team know of any side effects from taking the medicine or if you have had any emergency hospital admissions.

Your GP will not know if you have received active treatment (metformin) or placebo until after the end of the study.

Who has overall responsibility for the study?

The study Sponsor is the Newcastle upon Tyne Hospitals NHS Foundation Trust, who have overall responsibility for the study.

The doctor in charge of the study (the Chief Investigator) is Professor Miles Witham, a Consultant Geriatrician based in Newcastle upon Tyne.

The Newcastle University Clinical Trials Unit manages the study on behalf of the Sponsor.

Who is providing the study drug?

We have paid a company called ModePharma to make the metformin and placebo (dummy) tablets for this study. ModePharma specialise in making medicines for studies but they do not own the rights to make Metformin. ModePharma do not make profits by selling metformin. Your hospital pharmacy will give you the study medication (metformin or placebo) at the

For peer review only - http://bmjopen.bmj.com/site/about/guidelines.xhtml

end of the baseline visit. If your visit takes place at home, the tablets will be posted to you by Royal Mail Special Delivery or delivered by local courier. We will ask for your permission to send your name and address to the Royal Mail or the local courier.

What will happen to the results of the MET-PREVENT study?

- The results will be published in medical journals and presented at meetings to other doctors, nurses, researchers and patients.
- A report will be written for the study funder.
- A report must be written for the European Union Drug Regulating Authorities Clinical Trials (EudraCT) database.
- All study results that are published will be anonymous. This means that no-one will be able to find out who you are. Your identity will always be protected.
- The results will be available at the end of the study through publications, in the wider press and directly to patient groups.
- Fully anonymised data may be made available to other researchers to help inform other research studies.

What if I have a complaint or a problem occurs?

a) Complaints

If you have any concern or complaint about any aspect of this clinical study, please contact your local study team by phone, letter or email. Their contact details are listed at the end of this information sheet. If you are still unhappy and wish to raise your concerns with someone who is not directly involved in your care, you can contact <site to localise with local details such as local PALS phone number and email address>

You may also contact Patient Advice and Liaison Service (PALS) for confidential advice on any aspect of care on 0800 032 0202.

b) Harm

For peer review only - http://bmjopen.bmj.com/site/about/guidelines.xhtml

<local NHS Trust logo>

In the unlikely event that you are harmed during the study and this is due to NHS staff neglect, you may have grounds for legal action and compensation. This is organised through the NHS Indemnity (insurance) scheme. You may need to pay for your own legal costs. NHS Indemnity does not offer no-fault compensation (for harm that is not anyone's fault).

The Newcastle Clinical Trials Unit, part of Newcastle University, are managing the study on behalf of the study NHS Sponsor. Newcastle University also have indemnity arrangements. This covers Newcastle University staff involved in designing and managing the MET-PREVENT study.

Will my taking part in the study be kept confidential?

Yes. All the information that you provide during the course of this study will be securely stored. Paper copies of your study information will be stored in locked files or rooms at your local hospital. Electronic copies of your study information will be stored on a secure, password-protected computer database provided by Sealed Envelope™. Only authorised members of the study team will be granted access to the database.

- At study visits, your name will not be written on completed test forms or questionnaires. Instead, we will use a study code number (called a Participant Unique Study Identifier). This number is unique for you. No-one else taking part in the study will have this number. This number will also be used in the study database. Only the study team at your hospital will be able to link this number back to you using your date of birth, name and NHS number
- The study team at your hospital will have access to your information during the study. They will use this information to contact you to organise study visits as well as for ongoing safety.
- If you opt for study visits to take place in your own home, your hospital pharmacy will have access to your contact details to post the study medicine
- Your contact details will never be shared with anyone outside of the study. The exception is Royal Mail or a local courier. You will be asked to consent to the postal service or local courier your local hospital uses having access to your contact details. This is so that they can deliver the

For peer review only - http://bmjopen.bmj.com/site/about/guidelines.xhtml

<local NHS Trust logo>

study medicine to your home address. The postal or courier services will not know you are in the study, just that they need to deliver a package to you

- You will not be named in any results, reports or on websites
- Very occasionally, information might be given during the study that, by law, we must pass on to others. For instance, information which suggested you or others were at risk of harm. In this case, confidentiality would be broken so that we could pass this information to the relevant people. You would be informed of this.
- At the end of the study, all study information will be kept in a secure storage area for at least 15 years. This is called archiving. Archiving means that any queries about the running of the study can still be answered after the study has ended. All information will be held securely to make sure we protect your confidentiality. After the archiving period has ended, your information will be safely destroyed.
- If there are any unexpected serious side effects to the medicine, we would send details of this to the government medicines agency (MHRA). There is a specific form to do this, and only your study number will be sent to them.

Will you look at information from my existing medical records?

Yes. The study team at your hospital will be able to look at your GP and hospital medical records. They need to do this to collect information that is needed for you to take part in the study. For example, they will collect results of your blood tests, and prescriptions and health history.

Authorised people from your local NHS Trust, the MHRA, Sponsor (Newcastle upon Tyne Hospitals NHS Foundation Trust) and/or the Newcastle Clinical Trials Unit will also need to look at your medical records. This is to check that the study is being carried out to the correct standards. Everyone who looks at your medical records will have a duty of confidentiality to you as a research participant.

What will happen if I don't want to carry on with the study?

For peer review only - http://bmjopen.bmj.com/site/about/guidelines.xhtml

<local NHS Trust logo>

You can withdraw from the study completely at any time, for any reason. You do not have to tell the study team why you want to withdraw. You will always be fully cared for and supported in line with your GP and hospital's standard practice.
If you do give a reason for withdrawing from the study, we will ask if you are happy for us to record why you decided to withdraw.

If you withdraw from the study, we will keep the information about you that we have already collected. You are free to request that the study team destroys all information donated by you. By destroying your information, this means that it cannot be used at all for the remainder of the study. However, if some of your information has already been used in calculations and reports, it would not be possible to remove that information from these.

If I stop taking the medicine, do I have to leave the trial?

If you become unwell, your doctor may ask you to stop taking the study medicine. This may be just for a short while until it is safe for you to start taking it again.

You can stop taking the study medicine altogether, but stay in the study and continue with the study visits and assessments. We will always ask you what you would prefer to do.

What happens if I lose the capacity to consent during the study?

During the study, if you lose the capacity to make your own decisions, we will stop your medicine. The doctor may decide that you need to be withdrawn from the study. We will keep your information that has been collected up to this point.

If your doctor thinks you have recovered and can make your own decisions again, we will ask you if you want to continue to take part in the study.

Who is funding the MET-PREVENT study?

For peer review only - http://bmjopen.bmj.com/site/about/guidelines.xhtml

<local NHS Trust logo>

The National Institute for Health Research (NIHR) Newcastle Biomedical Research Centre funds the study. The UK government funds the NIHR to carry out research for the benefit of the NHS and its patients.

How have patients and the public been involved in the design of the study?

Volunteers from the local VOICE organisation (https://www.voice-global.org/) helped to design this study. They have also looked at the patient information sheet. We will ask a member of the public to join our study management group meetings.

Will my expenses be reimbursed?

Yes – we will pay for your travel expenses including providing a taxi if you need this to attend study visits at the hospital. Alternatively, transport may be arranged for you if your local hospital is able to offer this. Your local study team will manage any payments to reimburse costs to you and you may be asked to provide receipts for your travel.

Will I be paid for taking part?

We will not give you a payment for taking part, but we will pay for your transport and make sure that you have some food and drink at each trial visit.

HOW WILL WE USE INFORMATION ABOUT YOU?

Where 'we' is stated below, this means the study Sponsor – the Newcastle upon Tyne Hospitals NHS Foundation Trust.

We will need to use information from you, your medical records and your GP for this clinical research study.

This information will include your initials, date of birth, NHS number, name and contact details. People will use this information to do the research or to check your records to make sure that the research is being done properly.

For peer review only - http://bmjopen.bmj.com/site/about/guidelines.xhtml

<local NHS Trust logo>

People who do not need to know who you are will not be able to see your name or contact details. Your data will have a code number, called your unique study identifier, instead.

We will keep all information about you safe and secure.

Once we have finished the study, we will keep some of the data so we can check the results. We will write our reports in a way that no-one can work out that you took part in the study.

The Sponsor will provide Royal Mail or a local courier with your name and the address to allow Royal Mail or the courier to deliver your study medicine to you. Royal Mail and the local courier have their own policies about keeping personal information that comply with UK law. This also covers how they destroy your information. The policy for the Royal Mail Group and any local courier is to only keep information for as long as it is required for the purpose for which they use it.

What are your choices about how your information is used?

- You can stop being part of the study at any time, without giving a reason, but we will keep information about you that we already have.
- We need to manage your records in specific ways for the research to be reliable. This means that we won't be able to let you see or change the data we hold about you.
- If your medical condition does not affect your existing insurance policies, then taking part in a clinical trial should not affect your insurance. For existing insurance, or if you make a new application for any kind of insurance, then you must answer the insurer's questions honestly and accurately.

Where can you find out more about how your information is used?

You can find out more about how we use your information:

- www.hra.nhs.uk/information-about-patients/

For peer review only - http://bmjopen.bmj.com/site/about/guidelines.xhtml

<local NHS Trust logo>

- our leaflet available from https://www.newcastle-hospitals.nhs.uk/help/privacy/privacy-notice-for-patients/ and https://www.qegateshead.nhs.uk/research

- by asking one of the study team

- by sending an email to the trial Sponsor Data Protection Officer at nuth.dpo@nhs.net

Further Information and contact details

If you have any further questions or would like further information about the study or rights of participants, please feel free to contact the people below.

They are also who you or a doctor should contact in the event of an emergency.

<insert local Research Nurse name and contact details>

<insert local site PI name and contact details>

Thank you for reading this information sheet

FUNDED BY NIHR | National Institute for Health Research

This research is funded by the National Institute for Health (NIHR) Newcastle Biomedical Research Centre (BRC). The views expressed are those of the author(s) and not necessarily those of the NIHR or the Department of Health and Social Care.

For peer review only - http://bmjopen.bmj.com/site/about/guidelines.xhtml

SPIRIT

STANDARD PROTOCOL ITEMS: RECOMMENDATIONS FOR INTERVENTIONAL TRIALS

SPIRIT 2013 Checklist: Recommended items to address in a clinical trial protocol and related documents*

Section/item	ItemNo	Description	Page
**Administrative information**			
Title	1	Descriptive title identifying the study design, population, interventions, and, if applicable, trial acronym	1
Trial registration	2a	Trial identifier and registry name. If not yet registered, name of intended registry	3
	2b	All items from the World Health Organization Trial Registration Data Set	3
Protocol version	3	Date and version identifier	20
Funding	4	Sources and types of financial, material, and other support	23
Roles and responsibilities	5a	Names, affiliations, and roles of protocol contributors	23
	5b	Name and contact information for the trial sponsor	19
	5c	Role of study sponsor and funders, if any, in study design; collection, management, analysis, and interpretation of data; writing of the report; and the decision to submit the report for publication, including whether they will have ultimate authority over any of these activities	19
	5d	Composition, roles, and responsibilities of the coordinating centre, steering committee, endpoint adjudication committee, data management team, and other individuals or groups overseeing the trial, if applicable (see Item 21a for data monitoring committee)	18,24
**Introduction**			

For peer review only

Background and rationale	6a	Description of research question and justification for undertaking the trial, including summary of relevant studies (published and unpublished) examining benefits and harms for each intervention	5-7
	6b	Explanation for choice of comparators	5-7
Objectives	7	Specific objectives or hypotheses	5-7
Trial design	8	Description of trial design including type of trial (eg, parallel group, crossover, factorial, single group), allocation ratio, and framework (eg, superiority, equivalence, noninferiority, exploratory)	5-7
**Methods: Participants, interventions, and outcomes**			
Study setting	9	Description of study settings (eg, community clinic, academic hospital) and list of countries where data will be collected. Reference to where list of study sites can be obtained	7
Eligibility criteria	10	Inclusion and exclusion criteria for participants. If applicable, eligibility criteria for study centres and individuals who will perform the interventions (eg, surgeons, psychotherapists)	7-9, Table 1
Interventions	11a	Interventions for each group with sufficient detail to allow replication, including how and when they will be administered	9
	11b	Criteria for discontinuing or modifying allocated interventions for a given trial participant (eg, drug dose change in response to harms, participant request, or improving/worsening disease)	10
	11c	Strategies to improve adherence to intervention protocols, and any procedures for monitoring adherence (eg, drug tablet return, laboratory tests)	Table 2
	11d	Relevant concomitant care and interventions that are permitted or prohibited during the trial	9-10

For peer review only - http://bmjopen.bmj.com/site/about/guidelines.xhtml

Outcomes	12	Primary, secondary, and other outcomes, including the specific measurement variable (eg, systolic blood pressure), analysis metric (eg, change from baseline, final value, time to event), method of aggregation (eg, median, proportion), and time point for each outcome. Explanation of the clinical relevance of chosen efficacy and harm outcomes is strongly recommended	10-11
Participant timeline	13	Time schedule of enrolment, interventions (including any run-ins and washouts), assessments, and visits for participants. A schematic diagram is highly recommended (see Figure)	Table 2
Sample size	14	Estimated number of participants needed to achieve study objectives and how it was determined, including clinical and statistical assumptions supporting any sample size calculations	15
Recruitment	15	Strategies for achieving adequate participant enrolment to reach target sample size	15-16
**Methods: Assignment of interventions (for controlled trials)**			
Allocation:			
Sequence generation	16a	Method of generating the allocation sequence (eg, computer-generated random numbers), and list of any factors for stratification. To reduce predictability of a random sequence, details of any planned restriction (eg, blocking) should be provided in a separate document that is unavailable to those who enrol participants or assign interventions	16
Allocation concealment mechanism	16b	Mechanism of implementing the allocation sequence (eg, central telephone; sequentially numbered, opaque, sealed envelopes), describing any steps to conceal the sequence until interventions are assigned	16
Implementati on	16c	Who will generate the allocation sequence, who will enrol participants, and who will assign participants to interventions	16

For peer review only - http://bmjopen.bmj.com/site/about/guidelines.xhtml

Blinding (masking)	17a	Who will be blinded after assignment to interventions (eg, trial participants, care providers, outcome assessors, data analysts), and how	16
	17b	If blinded, circumstances under which unblinding is permissible, and procedure for revealing a participant's allocated intervention during the trial	16
**Methods: Data collection, management, and analysis**			
Data collection methods	18a	Plans for assessment and collection of outcome, baseline, and other trial data, including any related processes to promote data quality (eg, duplicate measurements, training of assessors) and a description of study instruments (eg, questionnaires, laboratory tests) along with their reliability and validity, if known. Reference to where data collection forms can be found, if not in the protocol	16-17
	18b	Plans to promote participant retention and complete follow-up, including list of any outcome data to be collected for participants who discontinue or deviate from intervention protocols	16-17
Data management	19	Plans for data entry, coding, security, and storage, including any related processes to promote data quality (eg, double data entry; range checks for data values). Reference to where details of data management procedures can be found, if not in the protocol	16-17
Statistical methods	20a	Statistical methods for analysing primary and secondary outcomes. Reference to where other details of the statistical analysis plan can be found, if not in the protocol	17
	20b	Methods for any additional analyses (eg, subgroup and adjusted analyses)	17-18
	20c	Definition of analysis population relating to protocol non-adherence (eg, as randomised analysis), and any statistical methods to handle missing data (eg, multiple imputation)	18
**Methods: Monitoring**			

Data monitoring	21a	Composition of data monitoring committee (DMC); summary of its role and reporting structure; statement of whether it is independent from the sponsor and competing interests; and reference to where further details about its charter can be found, if not in the protocol. Alternatively, an explanation of why a DMC is not needed	18,24
	21b	Description of any interim analyses and stopping guidelines, including who will have access to these interim results and make the final decision to terminate the trial	N/A
Harms	22	Plans for collecting, assessing, reporting, and managing solicited and spontaneously reported adverse events and other unintended effects of trial interventions or trial conduct	18-19
Auditing	23	Frequency and procedures for auditing trial conduct, if any, and whether the process will be independent from investigators and the sponsor	17
**Ethics and dissemination**			
Research ethics approval	24	Plans for seeking research ethics committee/institutional review board (REC/IRB) approval	19-20
Protocol amendments	25	Plans for communicating important protocol modifications (eg, changes to eligibility criteria, outcomes, analyses) to relevant parties (eg, investigators, REC/IRBs, trial participants, trial registries, journals, regulators)	19-20
Consent or assent	26a	Who will obtain informed consent or assent from potential trial participants or authorised surrogates, and how (see Item 32)	20
	26b	Additional consent provisions for collection and use of participant data and biological specimens in ancillary studies, if applicable	20
Confidentiality	27	How personal information about potential and enrolled participants will be collected, shared, and maintained in order to protect confidentiality before, during, and after the trial	20
Declaration of interests	28	Financial and other competing interests for principal investigators for the overall trial and each study site	23

For peer review only

Access to data	29	Statement of who will have access to the final trial dataset, and disclosure of contractual agreements that limit such access for investigators	20
Ancillary and post-trial care	30	Provisions, if any, for ancillary and post-trial care, and for compensation to those who suffer harm from trial participation	20
Dissemination policy	31a	Plans for investigators and sponsor to communicate trial results to participants, healthcare professionals, the public, and other relevant groups (eg, via publication, reporting in results databases, or other data sharing arrangements), including any publication restrictions	21
	31b	Authorship eligibility guidelines and any intended use of professional writers	21
	31c	Plans, if any, for granting public access to the full protocol, participant-level dataset, and statistical code	20-21
**Appendices**			
Informed consent materials	32	Model consent form and other related documentation given to participants and authorised surrogates	Not supplied
Biological specimens	33	Plans for collection, laboratory evaluation, and storage of biological specimens for genetic or molecular analysis in the current trial and for future use in ancillary studies, if applicable	Not supplied

*It is strongly recommended that this checklist be read in conjunction with the SPIRIT 2013 Explanation & Elaboration for important clarification on the items. Amendments to the protocol should be tracked and dated. The SPIRIT checklist is copyrighted by the SPIRIT Group under the Creative Commons "Attribution-NonCommercial-NoDerivs 3.0 Unported" license.

For peer review only - http://bmjopen.bmj.com/site/about/guidelines.xhtml

BMJ Open

MET-PREVENT: Metformin to improve physical performance in older people with sarcopenia and physical prefrailty/frailty – protocol for a double blind, randomised controlled proof of concept trial.

Journal:	*BMJ Open*
Manuscript ID	bmjopen-2022-061823.R1
Article Type:	Protocol
Date Submitted by the Author:	09-Jun-2022

Complete List of Authors:	Rennie, Katherine ; Newcastle Clinical Trials Unit<br>Witham, Miles; Newcastle University, NIHR Newcastle Biomedical Research Centre<br>Bradley, Penny; Newcastle Upon Tyne Hospitals NHS Trust, Pharmacy directorate<br>Clegg, Andrew; University of Leeds, Academic Unit of Elderly Care & Rehabilitation<br>Connolly, Stephen; Patient and Public Involvement Representative<br>Hancock, Helen; Newcastle University, Newcastle Clinical Trials Unit<br>Hiu, Shaun; Newcastle University Institute for Health and Society, Newcastle University<br>Marsay, Leanne; Newcastle University, Newcastle Clinical Trials Unit<br>McDonald, Claire; Gateshead Health NHS Foundation Trust<br>Robertson, Laura; Newcastle University, Newcastle Clinical Trials Unit<br>Simms, Laura; Newcastle University, Newcastle Clinical Trials Unit<br>Steel, Alison; Newcastle University, Newcastle Clinical Trials Unit<br>Steves, Claire J.; King's College London<br>Storey, Bryony; Gateshead Health NHS Foundation Trust<br>Wason, James; Newcastle University, Population Health Sciences Institute<br>Wilson, Nina; Newcastle University Faculty of Medical Sciences, Population Health Sciences Institute<br>von Zglinicki, Thomas ; Newcastle University, Campus for Ageing and Health<br>Sayer, Avan; Newcastle Upon Tyne Hospitals NHS Foundation Trust, NIHR Biomedical Research Centre

**Primary Subject Heading**:	Geriatric medicine
Secondary Subject Heading:	Rehabilitation medicine
Keywords:	GERIATRIC MEDICINE, CLINICAL PHARMACOLOGY, REHABILITATION MEDICINE

For peer review only - http://bmjopen.bmj.com/site/about/guidelines.xhtml

MET-PREVENT: Metformin to improve physical performance in older people with sarcopenia and physical prefrailty/frailty – protocol for a double blind, randomised controlled proof of concept trial.

Katherine J Rennie*[1]

Miles D Witham*[2]

Penny Bradley[3]

Andrew P Clegg[4]

Stephen Connolly[5]

Helen Hancock[1]

Shaun Hiu[6]

Leanne Marsay[1]

Claire McDonald[7]

Laura Robertson[1]

Laura Simms[1]

Alison J Steel[1]

Claire J Steves[8]

Bryony Storey[7]

James Wason[6]

Nina Wilson[6]

Thomas von Zglinicki[9]

Avan A Sayer[2]

*joint first author.

1. Newcastle Clinical Trials Unit, Newcastle University, Newcastle upon Tyne, NE2 4AE, UK.

2. AGE Research Group, NIHR Newcastle Biomedical Research Centre, Newcastle University and Newcastle upon Tyne Hospitals NHS Foundation Trust, Newcastle, UK

For peer review only - http://bmjopen.bmj.com/site/about/guidelines.xhtml

For peer review only

3. Pharmacy Directorate, Newcastle upon Tyne Hospitals NHS Foundation Trust, Newcastle Upon Tyne NE7 7DN

4. Academic Unit for Ageing and Stroke Research, University of Leeds, Bradford Royal Infirmary, Bradford, BD9 6RJ, UK.

5. Patient and Public Involvement Representative

6. Population Health Sciences Institute, Newcastle University, Newcastle upon Tyne, NE2 4AX, UK.

7. Queen Elizabeth Hospital, Gateshead Health NHS Trust, Gateshead, NE9 6SX, UK.

8. Kings College London and St. Thomas' Hospital, London, SE1 7EH, UK.

9. Ageing Research Laboratories, Newcastle University, Newcastle upon Tyne, NE4 5PL, UK.

Correspondence to:

Professor Miles D Witham, NIHR Newcastle Biomedical Research Centre, Newcastle University Campus for Ageing and Vitality, Newcastle upon Tyne, NE4 5PL, UK. Tel: +44 191 208 1317. Email: Miles.Witham@newcastle.ac.uk

Word count (abstract): 300

Word count (main text): 4958

Tables: 2

Figures: 2

For peer review only

For peer review only - http://bmjopen.bmj.com/site/about/guidelines.xhtml

Abstract

Introduction

Skeletal muscle dysfunction is central to both sarcopenia and physical frailty, which are associated with a wide range of adverse outcomes including falls and fractures, longer hospital stays, dependency, and the need for care. Resistance training may prevent and treat sarcopenia and physical frailty, but not everyone can or wants to exercise. Finding alternatives is critical to alleviate the burden of adverse outcomes associated with sarcopenia and physical frailty. This trial will provide proof of concept evidence as to whether metformin can improve physical performance in older people with sarcopenia and physical prefrailty or frailty.

Methods and analysis

MET-PREVENT is a parallel group, double-blind, placebo-controlled proof of concept trial. Trial participants can participate from their own homes, including completing informed consent and screening assessments. Eligible participants with low grip strength or prolonged sit-to-stand time together with slow walk speed will be randomised to either oral metformin hydrochloride 500mg tablets or matched placebo, taken three times a day for four months. The recruitment target is 80 participants from two secondary care hospitals in Newcastle and Gateshead, UK. Local primary care practices will act as Participant Identification Centres (PICs). Randomisation will be performed using a web-based minimisation system with a random element, balancing on sex and baseline walk speed. Participants will be followed up for four months post-randomisation, with outcomes collected at baseline and four months. The primary outcome measure is the four metre walk speed at the four month follow-up visit.

Ethics and dissemination

The trial has been approved by the Liverpool NHS Research Ethics Committee (20/NW/0470), the Medicines and Healthcare Regulatory Authority (EudraCT 2020-004023-16), and the UK Health

For peer review only - http://bmjopen.bmj.com/site/about/guidelines.xhtml

Research Authority (IRAS 275219). Results will be made available to participants, their families, patients with sarcopenia, the public, regional and national clinical teams, and the international scientific community.

Trial Registration

ISRCTN29932357

Keywords

Frailty, sarcopenia, older people, metformin, clinical trial.

Strengths and limitations of the study

- Seeks to enrol older people with sarcopenia and physical prefrailty or frailty – an under-served group
- Wide-ranging mechanistic studies embedded in the trial
- Flexible recruitment methods and flexible study visits including option for participants to be seen at home
- Limitations of short-term follow up and small sample size
- Will exclude some people with multimorbidity due to safety considerations

For peer review only - http://bmjopen.bmj.com/site/about/guidelines.xhtml

Background

Skeletal muscle dysfunction is central to both sarcopenia and physical frailty, which are common syndromes in older people. Sarcopenia, the loss of muscle strength and mass that commonly accompanies ageing [1] is a major health problem for many older people. Sarcopenia is a major risk factor for falls, hospitalisation, increased length of stay, care home admission and earlier death [2-4]. In addition, sarcopenia is an important component of the physical frailty syndrome, which is associated with a similar range of adverse outcomes [5]. Physical frailty is typically defined as the presence of three or more of five physical characteristics (weight loss, low energy expenditure, exhaustion, slow gait speed and low grip strength) [6]. The presence of one or two of these characteristics indicates the presence of physical prefrailty as a precursor to physical frailty. Impaired physical performance is therefore a key characteristic of both sarcopenia and physical frailty and is a target for intervention that is prioritised by patients [7].

At present, resistance exercise training is the only intervention proven to improve outcomes for people with sarcopenia or physical frailty, with limited evidence on its effects in physical prefrailty [8-10]. Not all older people with sarcopenia or physical frailty are able or willing to undertake resistance training. As a result, alternative therapeutic options are needed to both prevent and improve sarcopenia and physical frailty, including through targeting physical prefrailty as a precursor state to more advanced physical frailty. Although the aetiology of sarcopenia and physical frailty are incompletely understood, it is becoming clear that multiple fundamental biological pathways related to ageing are important in driving these syndromes. Key pathways include inflammation, mitochondrial dysfunction, neuromuscular junction dysfunction, cellular senescence, and dysregulation of nutrient sensing and intracellular metabolism [1,11].

Metformin, a biguanide molecule, has been used as a treatment for type 2 diabetes mellitus for decades; it is a generally safe and well tolerated therapy even in older people with physical frailty or

For peer review only - http://bmjopen.bmj.com/site/about/guidelines.xhtml

multiple health conditions. Importantly, the mode of action of metformin in lowering glucose in patients with type 2 diabetes does not rely on stimulating insulin release, and thus users are not subjected to an increased risk of hypoglycaemia. Metformin has pleiotropic effects on glucose metabolism and energy utilisation as well as on a range of other age-related pathophysiological pathways; several of these actions may be beneficial in treating the muscle dysfunction seen in sarcopenia and physical frailty as summarised in Figure 1 [12-23]. Not all mechanisms of action of metformin may be beneficial however. Some pathways (e.g. AMP kinase activation) are similar to those triggered by caloric restriction [24], which whilst potentially beneficial for longevity over a long period may have adverse catabolic consequences in the shorter-term. It is only by testing metformin in older people with sarcopenia that the balance of benefits and risks can be properly assessed.

Observational studies show lower rates of cardiovascular events and cancer mortality in metformin users [25,26], consistent with a wide range of physiological effects as outlined above. Few studies have examined the relationship between metformin use, sarcopenia and physical frailty. Such observational studies are challenging because metformin is currently indicated for use in diabetes mellitus – itself implicated in accelerated ageing, skeletal muscle dysfunction and earlier onset of physical frailty. However, incident and prevalent frailty-related diseases (including falls, weight loss, gait disorders, and frailty diagnosed using a cumulative deficits frailty index) have been found to be less common in patients with type 2 diabetes treated with metformin compared to those treated with other glucose-lowering agents in observational studies [26,27].

One previous trial conducted in Indonesia studied people aged 60 and over with prefrailty (defined by either the Fried criteria or by a cumulative deficits index) but without diabetes [28]. Participants were randomised to receive 16 weeks of 500mg metformin thrice daily or matched placebo. Walk speed increased significantly in the treatment group compared to placebo (by 0.13m/s; which exceeds the minimum clinically important difference of 0.10m/s [29]). The recent MASTERS randomised trial did

For peer review only - http://bmjopen.bmj.com/site/about/guidelines.xhtml

not show evidence that metformin enhanced the effect of resistance training, but the focus of this trial (augmentation of resistance training) was different and did not target people with sarcopenia or physical frailty [30].

Trial objectives

MET-PREVENT is a parallel group, randomised, double-blind, placebo-controlled trial. The primary objective of MET-PREVENT is to provide proof of concept evidence as to whether metformin is superior to placebo in improving physical performance in older people with sarcopenia and physical prefrailty or frailty. The secondary objectives are to elucidate potential mechanisms of action of metformin on sarcopenia and physical frailty by correlating changes in biomarkers with changes in physical performance measures between baseline and four months. A placebo-controlled design has been selected to minimise bias and is appropriate given the absence of other pharmacological interventions for sarcopenia or physical frailty.

Methods: Participants, interventions and outcomes

Trial Setting

Participants will be recruited from the catchment areas of two secondary care hospitals in the North-East of England. Participants will be approached through Older Peoples Medicine Clinics, Day Units, assessment units and rehabilitation facilities at the hospitals. In addition, local general practices (GPs) will act as Participant Identification Centres (PICs), a registry of patients with sarcopenia [31] and the National Institute for Health Research BioResource will be used to identify additional potential participants. A flexible approach to the setting for participation will be offered, with the option of either research centre, clinic or the participant's own home as the setting for trial visits.

Eligibility criteria

For peer review only - http://bmjopen.bmj.com/site/about/guidelines.xhtml

The target population for MET-PREVENT is older adults (≥65 years) with sarcopenia and either physical prefrailty or frailty. Sarcopenia is defined using the handgrip strength and sit to stand thresholds from the 2019 European Working Group on Sarcopenia in Older People (EWGSOP) guidance [32]. Low muscle mass is not used as an eligibility criterion in this trial; the 2019 EWGSOP guidance allows a diagnosis of probable sarcopenia without muscle mass measurement. Muscle mass is not routinely measured in clinical practice, and the omission of this criterion will improve the relevance of the trial results to clinical practice. Physical prefrailty and frailty are defined following the method of Fried et al [6]; participants will have a minimum of two of the five Fried criteria (and thus fulfil the definition for prefrailty) but may have three or more criteria (fulfilling the definition of frailty). The MET-PREVENT eligibility criteria are listed in Table 1. Exclusion criteria are primarily designed to exclude participants at higher risk of adverse events from taking metformin, those where metformin therapy is already indicated (e.g. diabetes mellitus), and other types of skeletal myopathy (e.g. steroid myopathy, heart failure myopathy) where confusion with sarcopenia might arise.

Table 1. MET-PREVENT inclusion and exclusion criteria

**Inclusion Criteria**
• Adults aged ≥ 65 years
• Low maximum handgrip strength (<16kg for women, <27kg for men) OR 5x sit to stand time >15 seconds
• Slow walk speed (<0.8 m/s on 4 metre walk test).

**Exclusion Criteria**
• Diabetes mellitus (type 1 or type 2)
• Previous intolerance of metformin or taking metformin for another condition
• Any contraindication to metformin, as listed in the current Summary of medicinal Product Characteristics for metformin

For peer review only - http://bmjopen.bmj.com/site/about/guidelines.xhtml

- Any medication which significantly interacts with metformin, as listed in the current Summary of medicinal Product Characteristics for metformin
- eGFR <45ml/min/1.73m^2 by MDRD4 or CKD-EPI equation
- History of diarrhoeal illness within the last three months (>48 hours of Bristol stool chart grade 6 or 7)
- Alcohol intake >21 units/week (women) or >35 units/week (men)
- Symptomatic chronic heart failure, diagnosed according to European Society of Cardiology guidelines (asymptomatic left ventricular systolic dysfunction will not be an exclusion criterion)
- Liver function tests (bilirubin, alanine aminotransferase or alkaline phosphatase) >3x upper limit of normal
- Oral steroid dose >7.5mg prednisolone equivalent per day
- Unable to mobilise without human assistance
- Unable to give written informed consent
- Life expectancy of <3 months as adjudicated by the local Investigator
- Participation in other interventional studies within 30 days prior to trial entry. Co-enrolment with other interventional studies is not allowed (observational studies and registries are permitted

Interventions

All randomised participants will take either metformin hydrochloride (500mg film-coated) tablets or matched placebo tablets, orally, three times a day with food or just after a meal. No dose adjustments are planned. Study medication is dispensed in a single bottle which is identical for metformin and placebo.

Investigators may discontinue the trial treatment in the event of side effects occurring, that are possibly, probably or definitely related to trial medication and which are not tolerable to the participant, or which constitute a serious adverse reaction (SAR) or suspected unexpected serious adverse reaction (SUSAR). Treatment will also be discontinued if the participant requests the medication to be withdrawn; in the event of a new diagnosis of diabetes mellitus (type 1 or type 2) or symptomatic chronic heart failure; if the estimated glomerular filtration rate (GFR) falls below

For peer review only - http://bmjopen.bmj.com/site/about/guidelines.xhtml

30ml/min/1.73m^2 or plasma lactate concentration is >4mmol/L in the safety follow up visit blood tests, or if lactic acidosis is diagnosed as part of clinical workup for an intercurrent illness. The trial medication may be temporarily discontinued at the instigation of the treating clinical team or the study team if an episode of acute kidney injury or an intercurrent illness with a risk of dehydration or hypovolemia occurs. Once this has resolved, trial medication may be restarted if the estimated GFR is >30ml/min/1.73m^2. Participants who discontinue allocated trial medication and wish to remain in the trial will be followed up as per their allocated treatment intervention arm. If a participant does not want to attend any further trial visits but is willing to complete questionnaires by phone/post then they may continue in the trial.

Outcomes

Primary Outcome

The primary outcome is the between-group difference in the four metre walk speed at four months. The four metre walk speed is performed from a standing start, with the participant asked to walk at their usual pace. Short-course walking speed is a powerful predictor of a range of adverse outcomes in older people, including death, dependency and cognitive impairment [33-35]. The four metre walk speed has been used as an outcome measure in previous trials, including those of metformin [30] and the minimum clinically important difference for this measure has been estimated to be 0.1m/s [28].

Secondary Outcome Measures

Secondary outcome measures in the MET-PREVENT trial include other measures of physical performance, health-related quality of life, mechanistic outcomes and outcomes related to trial performance. Maximum handgrip strength will be measured using a Jamar dynamometer [36]; three readings will be taken for each hand and the highest reading used. Six-minute walk distance will be recorded over a 10m course with standardised encouragement [37]. The Short Physical Performance Battery (SPPB) [38] will be measured as a composite test of lower limb function with the five-times sit

For peer review only - http://bmjopen.bmj.com/site/about/guidelines.xhtml

to stand time performed as part of the SPPB analysed as an additional, separate outcome. Lean body mass will be estimated using the Akern 101 bioimpedance system (Akern, Pontassieve, Italy), with the Sergi equation used to derive measures of total lean body mass [39]. The number of physical frailty characteristics (0-5) and the components of the score, and transitions between robust (0 characteristics), physical prefrailty (1-2 characteristics) and physical frailty (3+ characteristics) will be recorded.

Generic health-related quality of life will be recorded using the EQ5D-5L and SF-36 (physical and mental component summary scores) questionnaires [40,41]. Instrumental activities of daily living (ADLs) will be recorded using the Nottingham extended ADL (NEADL) score [42]. Glycosylated haemoglobin will be measured along with the homeostatic measure of insulin resistance (HOMA-IR) derived from peripheral glucose and insulin measures [43]. Advanced glycosylation end-products (AGE) presence in the skin will be measured by auto fluorescence using the AGE Reader (Diagnoptics, Gronigen, Netherlands) [44]. Blood samples will be processed and stored for later mechanistic studies, including measurement of a panel of pro-inflammatory cytokines and markers of cellular senescence and oxidative stress. Stool samples will be collected and stored for later microbiome studies. Metrics describing the conversion rate from screening to randomisation, and the retention rate of recruited participants over their four month study participation will also be collected.

Participant timeline

Participants who express interest in the trial will enter a brief pre-screening process by telephone to check provisional eligibility. Verbal consent will be sought for the pre-screen process, including access to medical records for further review of suitability for the trial. At pre-screening, participants are asked if they have a diagnosis of diabetes mellitus, are taking metformin, or have previously been intolerant of metformin. The SARC-F questionnaire will be administered [45]; this comprises five questions on physical function with a score between zero and ten. Based on previous data [46], a score of one or

For peer review only - http://bmjopen.bmj.com/site/about/guidelines.xhtml

more will be sufficient to identify those more likely to have sarcopenia and will enable progression to the screening visit. Participants who pass the pre-screen will be given or posted the full participant information sheet (PIS), and all participants will be given at least 48 hours to consider their participation. The PIS can be found in Appendix 1.

Written informed consent will be obtained at the screening visit, which may take place at home, at clinic, or in a research facility. After informed consent is given, information on demographics, medical and medication history and alcohol use will be collected. Maximum handgrip and 4 metre walk speed will be measured. Blood will be taken for urea, creatinine and electrolytes (U&Es) and liver function tests (LFTs) unless results are already available from within three months prior to the screening visit. Participant eligibility to proceed to the baseline visit is confirmed after the screening visit, once screening assessment results are available.

The schedule of events is given in Table 2. Main study outcomes are measured at baseline and the four month final visit; safety visits to check renal function, lactate concentrations and adverse events take place at one, two and three months, and a telephone call takes place one week after the baseline visit to ensure that the participant has received and started the study medication.

For peer review only

For peer review only - http://bmjopen.bmj.com/site/about/guidelines.xhtml

Table 2. MET-PREVENT trial schedule of events

Timepoint	Pre-Screening	Screen	Baseline	1 week (+ 3 days) after randomisation	1 month (+ 1 week) after randomisation	2 months (+ 1 week) after randomisation	3 months (+ 1 week) after randomisation	4 months +/-2 weeks
**SCREENING, CONSENT and PRE-ALLOCATION ASSESSMENTS**								
Brief Study Information Sheet and Invitation Letter posted/given to patients identified via SarcNet Registry, older peoples medicine clinics, day units and rehabilitation facilities and GP practices	X							
Telephone pre-screening (verbal consent, demographics, SARC-F tool) for participants who send a positive reply slip	X							
Patient Information Sheet posted to participants who pass pre-screen	X							
Informed written consent		X						
Eligibility assessments (U&Es, LFTs, demographics, medical history, concomitant medication, adverse events, four metre walk speed, 5x sit to stand time, grip strength)		X						
Eligibility confirmation		X						
Baseline assessments pre-allocation (blood glucose and HbA1c, height and weight, bioimpedance, AGE skin fluorescence, physical performance tests (SPPB, grip strength, six min walk), quality of life questionnaires (EQ5D5L, SF-36, Nottingham EADL), frailty screening questions (activity, exhaustion).			X					
Allocation (randomisation) - following completion of assessments			X					
Dispensing/posting trial medication			X					
**BIOLOGICAL SAMPLES**								

For peer review only - http://bmjopen.bmj.com/site/about/guidelines.xhtml

Blood and stool			X					X
**INTERVENTION**								
4 month supply of Metformin hydrochloride 500mg tablets or matching placebo tablets			←					→
**FOLLOW UP**								
Confirm medication receipt, ability to open medication bottle, date of first dose				X				
Safety blood tests (U&Es, LFTs, glucose, lactate)					X	X	X	X
Adverse event assessments		X	X	X	X	X	X	X
Concomitant Medications		X	X	X	X	X	X	X
Follow up assessments (blood glucose and HbA1c, weight, bioimpedance, AGE skin fluorescence, physical performance tests (SPPB, grip strength, six min walk), quality of life questionnaires (EQ5D5L, SF-36, Nottingham EADL), frailty screening questions (activity, exhaustion).								X
Return of unused trial medication								X
Medication compliance, accountability								X

For peer review only

For peer review only - http://bmjopen.bmj.com/site/about/guidelines.xhtml

Sample size

Based on previous work, we assumed a minimum clinically important difference for the four metre walk speed of 0.1m/s [28]. Assuming a SD of 0.24 as seen in the English Longitudinal Study of Ageing [47], and a correlation between baseline and six month measures of 0.8, as seen in a recent trial of older people at risk of falls [48] (a similar population to those who will be enrolled in this trial), a total of 66 participants would be needed (33 per group) to give 80% power to detect this difference at a two-sided alpha=0.05 in an analysis that is adjusted for baseline values. Recruiting 80 participants will allow for 17.5% dropout (higher than observed in previous trials of spironolactone and of vitamin D in similar populations [49,50]) to give 66 participants at follow up.

Recruitment

Recruitment will take place over ten months and participants will be followed up for four months; recruitment started in September 2021. Potential participants for MET-PREVENT will be identified by four routes. The SarcNet Registry [31] contains details of participants who have had grip strength and walk speed measured previously and have consented to be recontacted with offers to participate in other studies. The NIHR BioResource Centre Newcastle registry contains details of participants who have previously given consent for re-contact with information about potential trial opportunities suitable for them (REC REF: 18/NE/0138). Participants will also be sought via Older Peoples Medicine Clinics, Day Units, assessment units and rehabilitation facilities in participating centres. Where available, existing information on handgrip strength and walk speed held in the clinical record will be used to identify potential participants. Finally, potential participants will be identified via screening of general practice (GP) lists, in collaboration with the NIHR Primary Care Research Network. Where interrogation of the electronic Frailty Index (eFI) is possible [51], invitations will be targeted to those with at least mild frailty denoted by an eFI of 0.12 or greater.

For peer review only

For peer review only - http://bmjopen.bmj.com/site/about/guidelines.xhtml

Potentially eligible participants will be sent or given a brief study information sheet, an invitation letter with a reply slip and a prepaid envelope to express interest. The reply slip will be returned to the central study team, who will pass on details to the site recruiting teams. Those who are interested will be contacted for pre-screening as described below.

Assignment of intervention

Participants will be randomised on a 1:1 basis using an interactive web-based randomisation system (Sealed Envelope Ltd). Minimisation (with a 30% random element) will ensure balance across the two arms based on the following stratification variables: sex and baseline walk speed (<=0.6 or >0.6 m/s). The allocation sequence is prepared by Sealed Envelope and is concealed from the study team. Participants are allocated a pack number at randomisation; this pack number is used by the trial pharmacy to dispense pre-labelled medication packs. Participants, the clinical team and the study team (including investigators, research nurses collecting outcomes data, senior statistician and trial manager) are blind to treatment allocation during the trial. For the IDMC, the statisticians preparing the report will be partially blind. This means that analysis will be conducted by arm, but the statisticians will not know the treatment allocation for each arm. They will become unblinded if the IDMC requests fully unblinded data. The randomisation system has functionality for emergency unblinding. This will only occur for valid medical or safety reasons where it is necessary for the treating clinician to know which treatment the participant has been receiving; the randomisation system is able to inform the treating clinician by email without unblinding other members of the study team or the participant.

Data collection and management

The trial schedule of events is presented as a flow diagram (Figure 2) and a table of trial processes (Table 2). Recruited participants will be followed up for four months from the point of randomisation. Data including the number of participants identified, approached, pre-screened and screened will be

For peer review only - http://bmjopen.bmj.com/site/about/guidelines.xhtml

collected and documented on a site screening log. Data will be handled, computerised, stored and archived in accordance with the General Data Protection Regulation (2018), and the latest Directive on GCP (2005/28/EC). Patient identifiable data will remain at each site and will not be collected as part of the trial dataset. Patient identification on data collection tools used will be through a unique sequential screening number allocated by site staff. Data will be transcribed by site staff from data collection worksheets to the trial's secure, password-limited, validated database (Sealed Envelope Ltd, London, UK). The participant trial record, including completed paper data collection tools, will be archived at site for 15 years following the end of the trial. Newcastle Clinical Trials Unit (NCTU) staff monitor trial conduct and data integrity; this is detailed in a Data Management Plan and a Monitoring Plan approved by the trial Sponsor.

Statistical analysis

A Statistical Analysis Plan will document the planned analyses and will be finalised prior to database lock at the end of the trial. For the primary outcome (four metre walk speed at four months post-randomisation), the difference between arms will be tested through fitting a linear regression model that is adjusted for baseline four metre walk speed and sex. A suitable transform of the data (for example the best fitting Box-Cox transform) will be applied to ensure the data are approximately normally distributed. The model will allow estimation of the difference between arms together with the 95% confidence interval and p-value for testing the null hypothesis of no difference. Continuous secondary endpoints will be analysed in a similar manner to the primary outcome. Binary secondary endpoints will be analysed with a logistic regression model. All models will be adjusted for the outcome under test at baseline, baseline 4 metre walk speed and sex.

The following pre-planned subgroup analyses will be performed for the primary outcome: age >75 years vs <=75 years, men vs women, baseline walk speed >0.6m/s vs <=0.6m/s. In addition, a per-protocol analysis will be performed in participants with adherence to study medication >=80%.

For peer review only - http://bmjopen.bmj.com/site/about/guidelines.xhtml

The primary analysis will be a complete case analysis. In the event the mortality rate is >5%, we will perform a sensitivity analysis by imputing the worst score possible for the primary outcome (i.e. 0 m/s) in participants who died before the four month visit. A further sensitivity analysis will be performed by imputing the primary outcome to the worst score possible in the event both mortality and withdrawal due to illness is >5%. If more than 10% of primary outcome data is missing after imputation for missing-ness due to death and withdrawal due to illness, a sensitivity analysis using multiple imputation via chained equations using the baseline covariates in the imputation model will be performed. Twenty imputed datasets will be used.

Trial oversight

A Trial Management Group (TMG), facilitated by NCTU, will convene approximately monthly throughout the duration of the trial. Members will consist of key NCTU staff, the Chief Investigator, co-applicants, trial statisticians, local site research staff and a Sponsor representative. A patient and public involvement (PPI) representative will attend TMG meetings at a frequency determined on an ongoing basis. The TMG undertakes, with the additional of external membership, the role of a Trial Steering Committee for this small, relatively low risk CTIMP study. An Independent Data Monitoring Committee (IDMC) has been appointed to provide an independent review of participant safety. The independent members comprise two clinicians and a statistician. The IDMC will meet at least annually, and report directly to the TMG.

Patient and Public Involvement

A patient panel (convened by VOICE Global) consisting of older people with lived experience of muscle weakness was involved in the design of the trial protocol, selection of outcome measures and reviewed the participant information sheets. A lay member (SC) sits on the Trial Management Group and is involved in the overall management of the trial and in dissemination plans for the results.

For peer review only

For peer review only - http://bmjopen.bmj.com/site/about/guidelines.xhtml

Harms

Data from all adverse events (AEs) will be recorded on the trial database at every trial visit. Serious adverse events (SAEs) will be assessed for any relationship to the treatment intervention (causality), by a delegated, medically qualified site doctor. The following SAEs will be recorded on the database, but do not require reporting to Sponsor: any death or hospitalisation due to a new cardiovascular event, new diagnosis or treatment of cancer, fall or fracture, infection, delirium, reduced mobility, exacerbation of an existing medical condition, admission for elective or planned investigation or treatment, or hospitalisation due to nausea, vomiting, constipation or diarrhoea. The above exceptions to immediate SAE reporting refer only to SAEs where the trial medication is not deemed to be causally related to the event by the local site Principal Investigator. The Chief Investigator will assess, on behalf of Sponsor, expectedness (by reference to the approved Reference Safety Information) of any serious adverse reactions (SARs).

Safety assessments will be conducted at screening and at the one, two, three and four month follow up visits. Key safety data will include serum creatinine concentrations – eGFR will be calculated to ensure adequate renal function (>30ml/min/1.73m^2), liver function tests (LFTs), blood glucose and blood lactate concentrations. Lactate levels of >4mmol/L will lead to discontinuation of trial medication.

Consent

Informed, written consent will be sought at the screening visit, prior to conducting any trial procedures, including screening assessments. Where the screening visit takes place in the participant's home, the informed consent discussion with a delegated local investigator may take place in person or remotely

For peer review only - http://bmjopen.bmj.com/site/about/guidelines.xhtml

by telephone, teleconference or videoconference. A delegated research nurse will be present in the participant's home to facilitate this, and to countersign the consent form. The investigator will complete, date and sign a consent interview proforma to provide a comprehensive account of the remote consent interview.

Ancillary and post-trial care

No provision for continuation of trial medication will be made by the trial team or Sponsor. Metformin is not licensed for the indication under study in this trial; any off-licence use of metformin after the end of the trial would be the responsibility of the participant's usual primary or secondary care clinical team. Participants and their GP will be informed by letter of which treatment they took after all participants have completed their final visit and the database is locked.

Ethics and Dissemination

Ethics approval

A favourable ethical opinion has been granted from the UK Health Research Authority Research Ethics Committee (North-West - Liverpool Central Research Ethics Committee; trial reference approval number 20/NW/0470). The trial has also received approval from the UK Medicines and Healthcare products Regulatory Agency (MHRA; trial reference number 2020-004023-16). The trial has been included in the National Institute for Health Research Clinical Research Network (NIHR CRN) portfolio (NIHR CLRN study ID: 47772). The trial Sponsor is the Newcastle Upon Tyne Hospitals NHS Foundation Trust, Freeman Hospital, Freeman Road, High Heaton, Newcastle upon Tyne, NE7 7DN. The trial Sponsor has delegated responsibility for trial management to NCTU, including trial design; review and approval of all localised patient-facing documentation prior to implementation at each site; collection, analysis and interpretation of data; writing of the protocol publication and final clinical report

For peer review only - http://bmjopen.bmj.com/site/about/guidelines.xhtml

manuscripts. This protocol manuscript is based on version 7.0 of the trial protocol (dated 13th December 2021)

Dissemination policy

A final report of the trial will be provided to the Sponsor, Research Ethics Committee and the trial Funder within one year of the end of the study. The trial results will be uploaded to the European Union Drug Regulating Authorities Clinical Trials (EudraCT) database, as per the European Commission's guidelines on posting and publication of result-related information within 12 months. The trial is registered on the ISRCTN trial database and trial results will be made publicly available on the ISRCTN trial registry within 12 months of the end of the trial, defined as Last Patient Last Visit date. The final clinical trial report will be used for publication and presentation at scientific meetings. Summaries of results will also be made available to investigators for dissemination within their clinical areas and to the wider public, and a summary of results will be sent to all participants along with their treatment allocation. If feasible within pandemic meeting restrictions, we will hold a dissemination event for participants and their families to present and discuss the study results.

Access to data

Access to the full blinded dataset will be limited to the TMG and to authors of the trial publication. At the end of the trial, a de-identified dataset will be prepared and stored by Newcastle University. Requests for data sharing with study teams outside Newcastle University or the study Sponsor, including international requests, will be considered by a Data Access Committee with representation from the Funder, Sponsor and the trial Chief Investigator.

Discussion

For peer review only - http://bmjopen.bmj.com/site/about/guidelines.xhtml

Randomised controlled trials of treatments for sarcopenia and physical frailty are challenging to perform; previous trials have struggled to recruit their anticipated target numbers within the allocated time [52]. This is in part because sarcopenia (and to a lesser extent physical frailty) are not diagnoses commonly made or recorded in clinical practice and thus identifying people with these conditions is not straightforward. In addition, people with sarcopenia usually have multimorbidity [53] and by definition have components of the physical frailty syndrome. As a result, they may find it difficult to take part in clinical trials unless these trials are designed to facilitate their inclusion and retention.

We have sought to broaden the range of recruitment strategies employed to identify patients with sarcopenia and physical frailty. MET-PREVENT is one of the first sarcopenia trials to use a sarcopenia registry [31] to facilitate recruitment, and we also build on recent work deploying measures of muscle strength and walk speed into routine clinical practice to facilitate recruitment. Although recruitment through primary care lacks specificity for identifying those with sarcopenia or physical frailty, the use of the electronic frailty index can improve the specificity of searches, and large numbers of potentially eligible participants can be reached via this route. We have aimed to minimise the burden of study visits in MET-PREVENT, both in terms of visit duration and tasks to complete during each visit, but also by providing a flexible approach to the venue in which study visits are undertaken. Many patients with sarcopenia or physical frailty find it difficult to travel to study centres and some are unable to leave their home at all. Our flexible approach to study visits with many of them taking place in the homes of participants enables this group of patients who would normally be excluded from participation to take part in research.

Although the trial is powered to detect the minimum clinically important difference in four metre walk speed, it is not powered to detect clinically important differences for some of the secondary outcomes. However, the trial seeks to provide proof-of-concept for a larger, multicentre trial that would be powered to detect differences in these outcomes, including transitions from physical

For peer review only - http://bmjopen.bmj.com/site/about/guidelines.xhtml

prefrailty to frailty. The trial population excludes some groups for safety reasons (for instance those with chronic kidney disease) which limits the generalisability of the results. Muscle biopsies are not being performed, thus direct information on changes in skeletal muscle structure and function will not be obtainable from this trial.

The collection of blood and stool samples for future mechanistic analyses will maximise the scientific value of the trial but also look to identify those groups that may be more likely to benefit from metformin as an intervention in a future large trial. In addition, the mechanistic analyses (which will be performed after the main trial is complete) will identify which mechanistic pathways are most important in mediating any benefit of metformin and in doing so will help us identify pathways and featured targets for other interventions to prevent or treat sarcopenia and physical frailty. We believe that this study design provides a template for other phase II intervention trials for sarcopenia and physical frailty; this template should both accelerate progress in translational research and also enable easier comparison of results across different trials [54].

For peer review only

Author contributions

MDW is the trial Chief Investigator and senior author. MDW, AAS, AC, HH, CMcD, CS, and TvZ led the funding application and protocol development. JW was the trial Senior Statistical Advisor during the funding bid and advised on trial design. SH is the Trial Statistician. NW is the Senior Statistician and leads on the statistical analysis plan. PB is the Sponsor Pharmacy representative, and SC provided patient and public input. AJS, KJR, LM, LS and LR provided trial management and trial monitoring. KJR and MDW drafted the manuscript for this publication. All authors contributed to protocol development and critical revision of the manuscript.

Funding

For peer review only - http://bmjopen.bmj.com/site/about/guidelines.xhtml

This work was supported by the NIHR Newcastle Biomedical Research Centre, reference NU001533

Competing Interests

The authors declare that they have no competing interests.

Acknowledgements

The authors would like to thank the members of the MET-PREVENT Independent Data Monitoring Committee (DMC) for their continuing valuable expertise:

- DMC Chair - Dr Terry Quinn (Senior Lecturer and Honorary Consultant, University of Glasgow)
- DMC Statistician – Dr Lorna Aucott (Senior Statistician, Health Services Research Unit, University of Aberdeen)
- DMC Clinician - Dr Victoria Haunton (Honorary Senior Lecturer / Consultant Geriatrician, University of Plymouth/University Hospitals Plymouth NHS Trust).

References

[1] Cruz-Jentoft AJ, Sayer AA. Sarcopenia. *Lancet* 2019;393:2636-46.

[2] Lunt E, Ong T, Gordon AL, et al. The clinical usefulness of muscle mass and strength measures in older people: a systematic review. *Age Ageing* 2021;50:88-95.

[3] Liu P, Hao Q, Hai S, et al. Sarcopenia as a predictor of all-cause mortality among community-dwelling older people: A systematic review and meta-analysis. *Maturitas* 2017;103:16-22.

[4] Yeung SSY, Reijnierse EM, Pham VK, et al. Sarcopenia and its association with falls and fractures in older adults: A systematic review and meta-analysis. *J Cachexia Sarcopenia Muscle* 2019;10:485-500.

[5] Clegg A, Young J, Iliffe S, et al. Frailty in elderly people. *Lancet* 2013;381:752–62.

For peer review only - http://bmjopen.bmj.com/site/about/guidelines.xhtml

[6] Fried LP, Tangen CM, Walston J, et al. Frailty in older adults: evidence for a phenotype. *J Gerontol A Biol Sci Med Sci* 2001;56:M146-56.

[7] Roberts H, Khee TS, Philp I. Setting priorities for measures of performance for geriatric medical services. *Age Ageing* 1994;23:154-7.

[8] Cruz-Jentoft AJ, Landi F, Schneider SM, et al. Prevalence of and interventions for sarcopenia in ageing adults: a systematic review. Report of the International Sarcopenia Initiative (EWGSOP and IWGS). *Age Ageing* 2014;43:748-59.

[9] Moore SA, Hrisos N, Errington L, et al. Exercise as a treatment for sarcopenia: An umbrella review of systematic review evidence. *Physiotherapy* 2019;107:189-201.

[10] Apóstolo J, Cooke R, Bobrowicz-Campos E, et al. Effectiveness of interventions to prevent pre-frailty and frailty progression in older adults:a systematic review. *JBI Database System Rev Implement Rep* 2018;16:140–232.

[11] Morley JE. Pharmacologic Options for the Treatment of Sarcopenia. *Calcif Tissue Int* 2016;98:319-33.

[12] Murphy MP. How mitochondria produce reactive oxygen species. *Biochem J* 2009;417:1-13.

[13] Kane DA, Anderson EJ, Price JW, et al. Metformin selectively attenuates mitochondrial H2O2 emission without affecting respiratory capacity in skeletal muscle of obese rats. *Free Radic Biol Med* 2010;49:1082–7.

[14] Hardie DG. AMPK: positive and negative regulation, and its role in whole-body energy homeostasis. *Curr Opin Cell Biol* 2015;33:1–7.

[15] Saxton RA, Sabatini DM. mTOR Signaling in Growth, Metabolism, and Disease. *Cell* 2017;168:960-76.

[16] Castets P, Lin S, Rion N, et al. Sustained activation of mTORC1 in skeletal muscle inhibits constitutive and starvation-induced autophagy and causes a severe, late-onset myopathy. *Cell Metabol* 2013; 17:731–44.

For peer review only - http://bmjopen.bmj.com/site/about/guidelines.xhtml

[17] Kim SA, Choi HC. Metformin inhibits inflammatory response via AMPK-PTEN pathway in vascular smooth muscle cells. *Biochem Biophys Res Comm* 2012;425:866-72.

[18] Gu J, Ye S, Wang S, et al. Metformin inhibits nuclear factor-kappaB activation and inflammatory cytokines expression induced by high glucose via adenosine monophosphate-activated protein kinase activation in rat glomerular mesangial cells in vitro. *Chin Med J* 2014;127:1755-60.

[19] Andrews M, Soto N, Arredondo M. Effect of metformin on the expression of tumor necrosis factor-α, Toll like receptors 2/4 and C reactive protein in obese type-2 diabetic patients. *Revista Medica De Chile* 2012;140:1377-82.

[20] Prattichizzo F, Giuliani A, Mensà E, et al. Pleiotropic effects of metformin: Shaping the microbiome to manage type 2 diabetes and postpone ageing. *Ageing Res Rev* 2018;48:87-98.

[21] Heintz C, Mair W. You are what you host: microbiome modulation of the aging process. *Cell* 2014;156:408-11.

[22] Jackson MA, Verdi S, Maxan ME, et al. Gut microbiota associations with common diseases and prescription medications in a population-based cohort. *Nat Commun* 2018;9:2655.

[23] Short S, Fielder E, Miwa S, et al. Senolytics and senostatics as adjuvant tumour therapy. *EBioMedicine* 2019;41:683-92.

[24] Madeo F, Carmona-Gutierrez D, Hofer SJ, Kroemer G. Caloric Restriction Mimetics against Age-Associated Disease: Targets, Mechanisms, and Therapeutic Potential. *Cell Metabol* 2019;29:592-610.

[25] Xu H, Aldrich MC, Chen Q, et al. Validating drug repurposing signals using electronic health records: a case study of metformin associated with reduced cancer mortality. *Am Med Inform Assoc* 2015;22:179–91

[26] Wang CP, Lorenzo C, Habib SL, et al. Differential effects of metformin on age related comorbidities in older men with type 2 diabetes. *J Diabet Complic* 2017;31:679-86.

[27] Sumantri S, Setiati S, Purnamasari D, et al. Relationship between metformin and frailty syndrome in elderly people with type 2 diabetes. *Acta Med Indones* 2014;46:183–8.

For peer review only

For peer review only - http://bmjopen.bmj.com/site/about/guidelines.xhtml

[28] Laksmi PW, Setiati S, Tamin TZ, et al. Effect of Metformin on Handgrip Strength, Gait Speed, Myostatin Serum Level, and Health-related Quality of Life: A Double Blind Randomized Controlled Trial among Non-diabetic Pre-frail Elderly Patients. *Acta Med Indones* 2017;49:118-27.

[29] Perera S, Mody SH, Woodman RC, et al. Meaningful change and responsiveness in common physical performance measures in older adults. *J Am Geriatr Soc* 2006;54:743-9.

[30] Walton RG, Dungan CM, Long DE, et al. Metformin blunts muscle hypertrophy in response to progressive resistance exercise training in older adults: A randomized, double-blind, placebo-controlled, multicenter trial: The MASTERS trial. *Aging Cell* 2019;18:e13039.

[31] Witham MD, Heslop P, Dodds RM, et al. Developing a UK sarcopenia registry: recruitment and baseline characteristics of the SarcNet pilot. *Age Ageing* 2021;50:1762-9.

[32] Cruz-Jentoft AJ, Bahat G, Bauer J, et al. Sarcopenia: revised European consensus on definition and diagnosis. *Age Ageing* 2019;48:16-31.

[33] Studenski S, Perera S, Patel K, et al. Gait speed and survival in older adults. *JAMA* 2011;305:50-8.

[34] Hoogendijk EO, Rijnhart JJM, Skoog J, et al. Gait speed as predictor of transition into cognitive impairment: Findings from three longitudinal studies on aging. *Exp Gerontol* 2020;129:110783.

[35] Abellan van Kan G, Rolland Y, Andrieu S, et al. Gait speed at usual pace as a predictor of adverse outcomes in community-dwelling older people an International Academy on Nutrition and Aging (IANA) Task Force. *J Nutr Health Aging* 2009;13:881-9.

[36] Roberts HC, Denison HJ, Martin HJ, et al. A review of the measurement of grip strength in clinical and epidemiological studies: towards a standardised approach. *Age Ageing* 2011;40:423-9.

[37] Guyatt GH, Sullivan MJ, Thompson PJ, et al. The 6-minute walk: a new measure of exercise capacity in patients with chronic heart failure. *Can Med Assoc J* 1985;132:919-23.

[38] Guralnik JM, Simonsick EM, Ferrucci L, et al. A short physical performance battery assessing lower extremity function: association with self-reported disability and prediction of mortality and nursing home admission. *J Gerontol* 1994;49:M85-94.

For peer review only

For peer review only - http://bmjopen.bmj.com/site/about/guidelines.xhtml

[39] Sergi G, De Rui M, Veronese N, et al. Assessing appendicular skeletal muscle mass with bioelectrical impedance analysis in free-living Caucasian older adults. *Clin Nutr* 2015;34:667-73.

[40] Herdman M, Gudex C, Lloyd A, et al. Development and preliminary testing of the new five-level version of EQ-5D (EQ-5D-5L). *Qual Life Res* 2011;20:1727–36.

[41] Ware JE, Kosinski M, Bjorner JB, et al. User's Manual for the SF-36v2TM Health Survey. 2nd edition. Lincoln (RI): QualityMetric; 2007.

[42] Nouri FM, Lincoln NB. An extended activities of daily living scale for stroke patients. *Clin Rehabilitation* 1987;1:301-5.

[43] Matthews DR, Hosker JP, Rudenski AS, et al. Homeostasis model assessment: insulin resistance and beta-cell function from fasting plasma glucose and insulin concentrations in man. *Diabetologia* 1985;28:412-9.

[44] Drenth H, Zuidema SU, Krijnen WP, et al. Advanced Glycation End Products Are Associated With Physical Activity and Physical Functioning in the Older Population. *J Gerontol A Biol Sci Med Sci* 2018;73:1545-51.

[45] Malmstrom TK, Morley JE. SARC-F: a simple questionnaire to rapidly diagnose sarcopenia. *J Am Med Dir Assoc* 2013;14:531-2.

[46] Dodds RM, Murray JC, Robinson SM, et al. The identification of probable sarcopenia in early old age based on the SARC-F tool and clinical suspicion: findings from the 1946 British birth cohort. *Eur Geriatr Med* 2020;11:433-441.

[47] Witham MD, Wason J, Dodds R, et al. Developing a composite outcome measure for frailty prevention trials - rationale, derivation and sample size comparison with other candidate measures. *BMC Geriatr* 2020;20:113.

[48] Witham MD, Price RJG, Band MM, et al. Effect of Vitamin K2 on Postural Sway in Older People Who Fall: A Randomized Controlled Trial. *J Am Geriatr Soc* 2019;67:2102-7.

[49] Burton LA, Sumukadas D, Witham MD, et al. Effect of spironolactone on physical performance in older people with self-reported physical disability. *Am J Med* 2013;126:590-7.

For peer review only

For peer review only - http://bmjopen.bmj.com/site/about/guidelines.xhtml

[50] Witham MD, Price RJ, Struthers AD, et al. Cholecalciferol treatment to reduce blood pressure in older patients with isolated systolic hypertension: the VitDISH randomized controlled trial. *JAMA Intern Med* 2013;173:1672-9.

[51] Clegg A, Bates C, Young J, et al. Development and validation of an electronic frailty index using routine primary care electronic health record data. *Age Ageing* 2016;45:353-60.

[52] Witham MD, Achison M, Aspray TJ, et al. Recruitment strategies for sarcopenia trials: lessons from the LACE randomized controlled trial. *JCSM Rapid Comms* 2021;4:93-102.

[53] Dodds RM, Granic A, Robinson SM, et al. Sarcopenia, long-term conditions, and multimorbidity: findings from UK Biobank participants. *J Cachexia Sarcopenia Muscle* 2020;11:62-8.

[54] Witham MD. Bridging the gap between the laboratory and the clinic for patients with sarcopenia. *Biogerontology* 2019;20:241-8.

For peer review only

Figure 1. Potential mechanisms of action through which metformin could improve skeletal muscle function

AMP Kinase: Adenosine MonoPhosphate activated protein Kinase

mTOR: mammalian Target Of Rapamycin

Figure 2. MET-PREVENT trial flow diagram

For peer review only

For peer review only - http://bmjopen.bmj.com/site/about/guidelines.xhtml

For peer review only

For peer review only - http://bmjopen.bmj.com/site/about/guidelines.xhtml

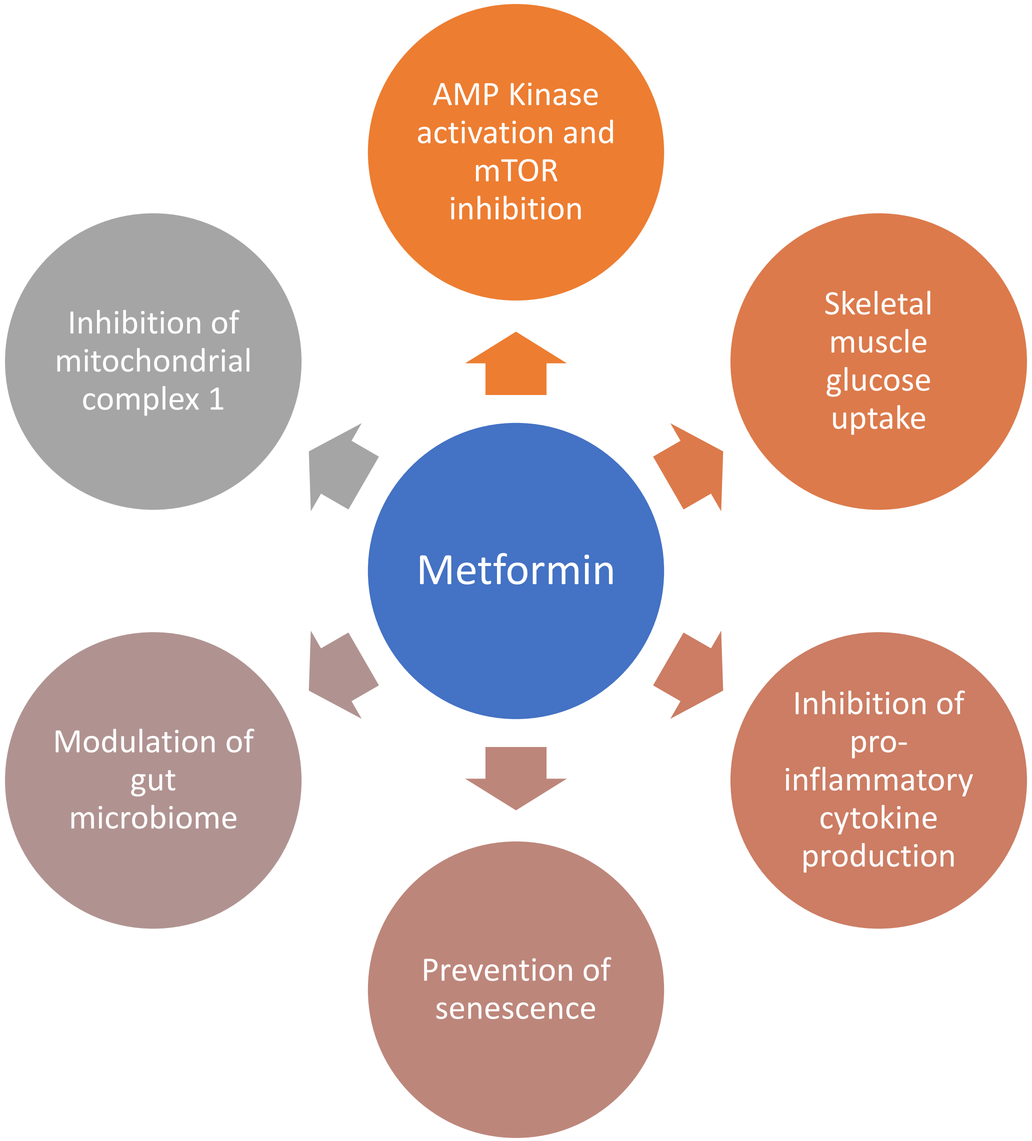
AMP Kinase activation and mTOR inhibition
Skeletal muscle glucose uptake
Inhibition of pro-inflammatory cytokine production
Prevention of senescence
Modulation of gut microbiome
Inhibition of mitochondrial complex 1
Metformin

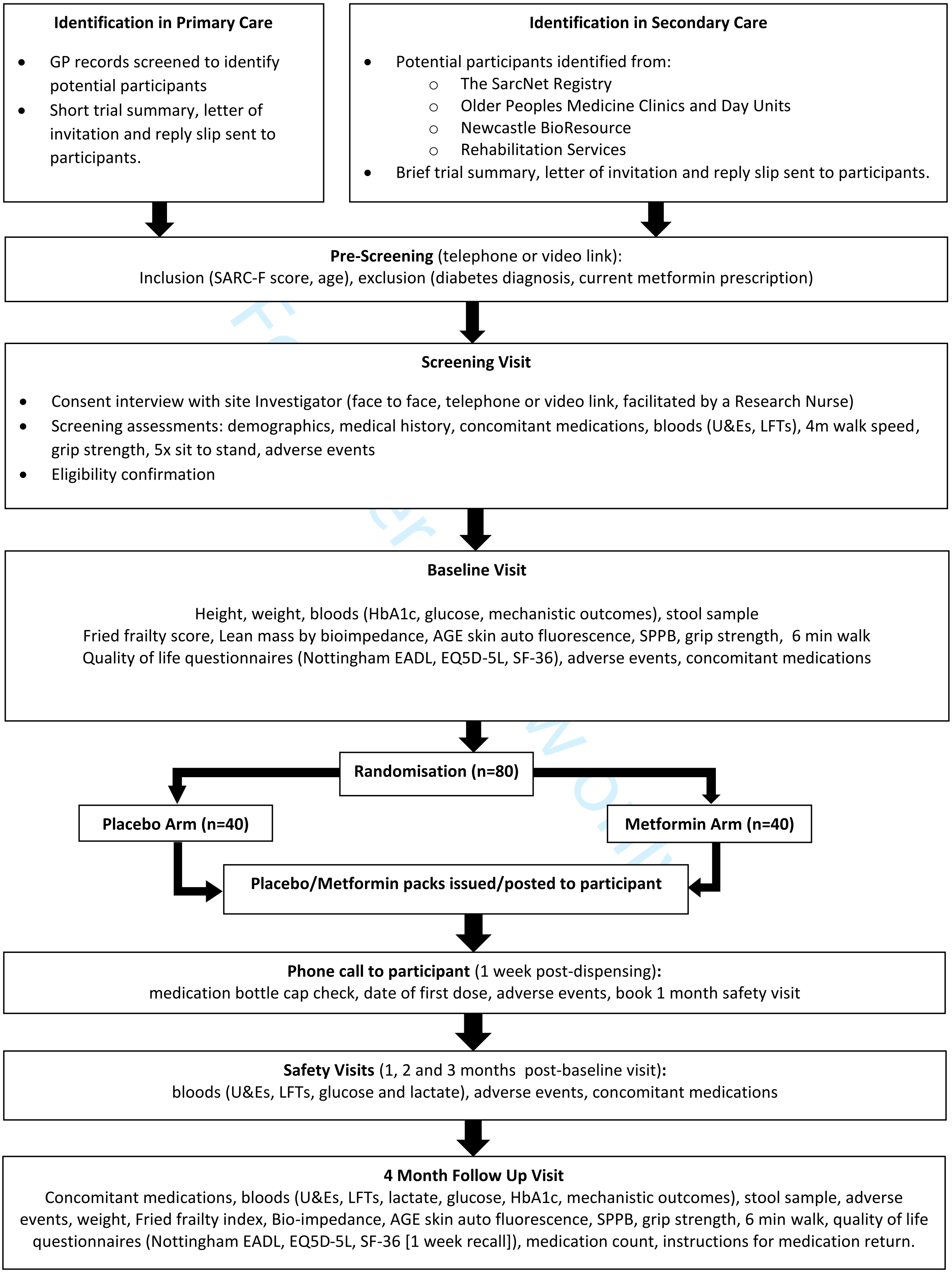

For peer review only - http://bmjopen.bmj.com/site/about/guidelines.xhtml

<local NHS Trust logo>

MET-PREVENT

Metformin to prevent progression to frailty for older people

– a randomised controlled proof of concept trial

Introduction

Thank you for considering taking part in the MET-PREVENT study. Before you decide whether to take part, please take time to read this information about the study carefully. Feel free to discuss it with your family, friends, carers or your GP if you wish to do so. If anything is unclear, or you need more information, please ask. You will find our contact details at the back of the information. This leaflet is yours to keep.

For peer review only

SUMMARY of the MET-PREVENT STUDY

**Purpose of the study**	• We would like to find out whether a medicine called metformin (used to treat type 2 diabetes) can help people over the age of 65 with muscle weakness but without diabetes to become stronger and improve their daily activities.
**Will I definitely get metformin?**	• No. To ensure that the study is a fair test, a computer will randomly allocate you to either metformin or the placebo (dummy) tablets. There is an equal chance that you will receive metformin or the dummy tablets. Both types of tablet are identical. Neither you nor the doctor, nurse or study team will know which treatment you are receiving.
**What are the side effects of the medicine?**	• The medicine may cause nausea (feeling sick) or loose bowels in some people. Most people do not get either

For peer review only - http://bmjopen.bmj.com/site/about/guidelines.xhtml

Newcastle University

<local NHS Trust logo>

of these effects. These side effects usually disappear after a few days or a couple of weeks.

If I take part, what will I have to do?

- All of the study visits may take place in your own home, at a research centre, or at a hospital clinic.
- You will be asked to attend a screening visit. The doctor will discuss the study with you and you can ask questions. We will ask to take a small blood sample to see if it is safe for you to take part.
- If you are suitable, we will ask you to take part in another 5 visits (3 of these are short safety visits).
- You will be asked to take a tablet 3 times a day for 4 months.
- We will ask you to give small blood samples at every visit (6 teaspoons), and a small stool (poo) sample at 2 visits (you can collect this in advance of the visit).
- At 2 visits we will ask you to do some simple tests that involve balance, walking a short distance, getting up from a chair and gripping a measuring device with your hand. You will also be asked to complete 3 questionnaires that ask about your daily activities.

Do I have to visit the hospital to take part?

- Not if you don't want to. We can visit you in your own home if you wish. A member of the local clinical team will bring all the equipment.

Do I have to take part?

- No – it is entirely your choice whether you wish to take part or not. Your care will not be affected in any way.

If you are still interested in taking part in the MET-PREVENT study, please read on for more information.

For peer review only - http://bmjopen.bmj.com/site/about/guidelines.xhtml

<local NHS Trust logo>

Figure 1. Summary of the MET-PREVENT study visits

SCREENING VISIT – 1 to 2 HOURS

- Written informed consent with doctor
- Medical history, other medicines
- Physical tests (short walk, sit to stand and handgrip strength)
- Blood tests (2 teaspoons)
- After visit - eligibility for the study confirmed

BASELINE VISIT – 1 to 2 HOURS

- Have stool sample ready
- Height and weight
- Physical tests (walking, balance, sit to stand and handgrip strength)
- Light beam skin test and muscle measurement
- 3 questionnaires
- Blood samples (6 teaspoons)

1, 2 and 3 MONTH FOLLOW UPS – about 30 minutes

- Medicines review
- Review of any episodes of ill health
- Safety blood sample (2 teaspoons)

4 MONTH FOLLOW UP (FINAL) VISIT – 1 to 2 HOURS

- Have stool sample ready
- Height and weight
- Physical tests (walking, balance, sit to stand and handgrip strength)
- Light beam skin test and muscle measurement
- 3 questionnaires
- Blood samples (6 teaspoons)
- Review of other medicines
- Return unused study medicine

For peer review only - http://bmjopen.bmj.com/site/about/guidelines.xhtml

Newcastle University

<local NHS Trust logo>

What is the purpose of the MET-PREVENT study?

Many of us lose muscle size and strength as we get older – this is called sarcopenia. People with weaker muscles are more likely to fall over and may start to have problems carrying out normal daily activities. They may also take longer to recover from other illnesses. Falling, struggling with daily living and taking a long time to get better from being unwell is a condition called frailty.

The best way to keep up your muscle strength is to do strengthening exercises, but not everyone wants to, or is able to do these.

Metformin is a medicine that is already safely used in older people to treat type 2 diabetes. Recent research suggests that metformin might help to improve muscle strength, even in people who do not have diabetes. To know if this is correct, we need to test metformin in a clinical trial. The MET-PREVENT study will test if metformin can improve muscle strength in older people who have signs of muscle weakness.

What are the possible side effects of the study medicine?

As with any medicine, the medicine used in this study (**metformin**) may cause side effects in some people. Doctors in England write millions of prescriptions for metformin every year, and the side effects are well understood.

In some people, metformin can cause nausea (occasionally vomiting), loss of appetite, tummy ache or loose bowel motions. However, we are using a low dose of metformin to reduce the chance of any of these side effects. These effects usually occur during the first few weeks of treatment and then ease in most people. To help prevent them, we will ask you to take your study medicine in 3 daily doses during or just after meals.

If you become unwell for some other reason, metformin can cause a build-up of acid in the blood. This is very rare. Occasionally, metformin may also cause a skin rash or irritate the liver. We will do regular blood tests to watch out for these problems. If you do become ill for some other reason, your doctors may stop the study medicine for a few days until you get better. This helps to prevent acid build-up in the blood. **More detail on these side effects is provided in the Supporting Information on page 10.**

For peer review only - http://bmjopen.bmj.com/site/about/guidelines.xhtml

<local NHS Trust logo>

We will issue you with a safety card that will have your medicine dose printed on it. The safety card will also have the contact details of the study team at your local hospital. If you see other healthcare professionals, you should show them the safety card to let them know that you are taking part in a clinical study.

The 4 month follow-up visit is the last study visit. For the 4 weeks (28 days) following on from this final visit, we would like to know if you have been unwell. We will ask you to contact your study doctor or a member of their team to tell them about this. You can contact your study doctor and team on the contact details given on the last page of this information sheet.

If you are unsure whether the study doctor needs to know about an illness or not, please get in touch with your study doctor or a member of their team to check.

What would I have to do if I took part?

We will ask you to attend 6 visits, either at hospital or in your own home:

- Screening visit
- Baseline visit
- 1, 2 and 3 month safety follow-up visits
- 4 month follow-up visit

These visits are extra, and not part of your usual NHS care. There is a diagram (Figure 1) on page <insert page number>that shows the details of the trial visits.

Screening Visit

At the screening visit the doctor will discuss the study with you, and you will be able to ask questions. If this visit takes place in your home, a member of the research clinical team (usually a research nurse) will use a phone to let you talk to the doctor. This may be over a videolink. We will then ask if you are happy to give written consent to take part in the study. We will ask you some questions about yourself and your health. We will also ask to take a small blood sample (2 teaspoons) to make sure that it is safe for you to take part in

For peer review only - http://bmjopen.bmj.com/site/about/guidelines.xhtml

<local NHS Trust logo>

this study. We will ask you to walk a short distance (4 metres), stand up and sit down from a chair five times, and test your handgrip strength.

We will ask you if you are able to open child-resistant caps on medicine bottles

When we have the results of the blood test, we will call you and arrange the next visit.

Baseline Visit

We will ask you to take part in a number of assessments before you start the study medicine. We will ask that you have a small stool (poo) sample ready in advance. We will ask you to do the following:

- We will measure your weight and height.
- Walk a short distance (4 metres), with your usual walking aids.
- Walk for 6 minutes, with your usual walking aids.
- To test your balance, we will ask you to stand beside the researcher for a short time (no more than 30 seconds).
- Stand up from a chair.
- Use a handgrip on both hands.
- Put sticky pads on your hand and foot connected to a device that measures the amount of muscle in your body.
- Measure the response of your skin to shining a light beam on it. This device tells us about different substances inside your skin without needing to take a skin sample).
- Complete 3 questionnaires that ask how you feel about your health and your activities of daily living (such as washing and cooking).
- Have a small amount of blood taken (6 teaspoons).
- Ask about other medicine that you may be taking.

The study medicine will then be issued to you. If you are attending hospital, there will be a short wait for this. If the baseline visit takes place in your own home, or if the wait at hospital may be too long, the study medicine will be posted to you via Royal Mail or delivered by local courier. A member of the study team will phone you to check that you have received the medicine. They will also check that you can open the bottle, and have taken the first dose.

For peer review only - http://bmjopen.bmj.com/site/about/guidelines.xhtml

<local NHS Trust logo>

1, 2 and 3 month follow up visits

These visits are to check that it is safe for you to continue in the study. To do this, we will ask you for a small blood sample (2 teaspoons) at each visit. We will ask you for a list of all of your other medicines that you take. We will also collect information about all episodes of ill-health that you have experienced in the past month.

4 month follow up visit

This the last study visit. We will ask you to have a stool (poo) sample ready. We will ask you to repeat all of the tests that you did at the baseline visit, including the blood samples. We will also collect any unused trial medicine.

What are the benefits and disadvantages of taking part in this study?

We cannot promise the study will help you directly. However, the information we collect from this study may help to improve the treatment for people with muscle weakness. If you want to find out more about taking part in research studies, you can visit the NHS Choices website www.nhs.uk.

As with any medicine, metformin can cause side effects in some people. We are using a low dose to reduce the chance of this happening. If you do get a side effect from the study medicine, your doctor can stop your medicine. The side effects should disappear rapidly. In case of an emergency, the doctors can find out which treatment you are taking (metformin or placebo) if they need to.

Is it safe for me to take part in this study during COVID-19?

The study will only take place if your hospital says that it is safe to do so. You can take part in the study in your own home if you wish to avoid visiting the hospital. The study team will follow all of the hospital COVID-19 policies and procedures when they visit you, including wearing masks, gloves and visors. These will be explained to you when your appointments are arranged.

For peer review only - http://bmjopen.bmj.com/site/about/guidelines.xhtml

Newcastle University

<local NHS Trust logo>

What happens to my blood and stool samples?

At the baseline and 4 month visits, giving the stool (poo) sample and the large blood sample (4 teaspoons) is optional. We still need a small blood sample for safety tests (2 teaspoons). The samples will be stored in a licenced Newcastle University Biobank at the end of the study. **Your consent to store these additional samples in the biobank is optional.** Your biobank samples may be used in further research linked to this study. They may also be used by other researchers in different research studies. Your samples can only be identified by using your unique study identity number. Researchers who use your samples will not know who you are.

Other researchers may want to use parts of your biobank sample in animal or commercial (paid) research. You can give samples to the biobank, but opt out of them being used for animal or paid research.

Pregnancy

We know that female participants over the age of 65 years cannot become pregnant.

However, if you are a male participant, we will ask you to inform us if your female partner becomes pregnant, or is breast-feeding an infant. We will ask for your consent to do this. We will also ask your partner to sign a consent form. This will allow the study team to collect safety information about their pregnancy and their baby.

What happens at the end of the study?

At the end of the study (4 months) you will stop taking the study medicine. You will continue to receive standard care like any other patient with your condition under the care of your GP and/or hospital doctor.

When everyone has completed the study, we will analyse the results and we will tell you what the results are. We can either invite you to a study event to or send you a written newsletter – whichever you prefer.

For peer review only - http://bmjopen.bmj.com/site/about/guidelines.xhtml

<local NHS Trust logo>

We would like to follow your progress with your health over the next 5 years. We will do this by looking at your medical records, and we will ask your permission to do this. You do not have to do anything else.

Who do I contact for further information?

We will be happy to answer any questions you, your family or your carers may have about any aspect of this clinical study. Please call the number at the end of this booklet to speak to the Research Nurse at your local hospital.

For peer review only

For peer review only - http://bmjopen.bmj.com/site/about/guidelines.xhtml

<local NHS Trust logo>

SUPPORTING INFORMATION

Metformin side effects

Very common – nausea, vomiting, diarrhoea, abdominal pain and loss of appetite (this usually stops in a few weeks in most people).

Common – taste disturbance.

Very rare – lactic acid build up in the blood

Very rare - abnormalities in liver function tests or hepatitis (goes away after metformin is stopped).

Very rare – skin redness, itching, hives.

Very rare – decreased vitamin B12 absorption (only seen with long term use of metformin or in patients who already have a vitamin B12 absorption problem called megaloblastic anaemia)

Why was I contacted?

We have contacted you because you are aged 65 or over and our measurements or questionnaire results suggest that your muscles are not as strong as they used to be. This trial is testing a medicine (metformin) to see if we can prevent further weakening of muscles in people like you.

Do I have to take part?

No - it is up to you to decide whether or not to take part in this study. You do not have to take part. If you choose not to, you will continue to get the standard care arranged by your doctor.

If you agree to take part, we will ask you to sign a consent form. We will give you a copy of your signed consent form which is yours to keep. You can still change your mind and withdraw at any time without having to give a reason. If you decide not to take part, or withdraw from the study later on, your current or future medical care will not be affected in any way.

What does giving consent mean for me?

By signing a consent form, this means that you fully understand what taking part in the study means for you. That's why it is really important that you take as much time as you want to read this information sheet. Feel free to discuss

For peer review only - http://bmjopen.bmj.com/site/about/guidelines.xhtml

Newcastle University

<local NHS Trust logo>

the study with your family, friends or any healthcare professional. At the screening visit, you will discuss the study with a study doctor, who will answer all of your questions.

Will I know what treatment I am on?

No. A computer will decide whether you will be given either the active medicine (metformin) or the placebo (dummy) tablets for 4 months. There is an equal chance that you will be given metformin or placebo tablets. The metformin and placebo tablets look identical. This means that neither you, the researchers, nor your medical teams will know what tablets you are taking. If no-one knows what tablets you are taking, no-one can influence the results of the study.

At the end of the study, when everyone has completed their visits, you may find out which tablets you were taking. We will also write to your GP to tell them what tablets you were taking. We will ask your permission to do this.

Who has checked the scientific quality of the MET-PREVENT study?

Independent experts at Newcastle University have checked the quality of the science used to plan this study. The study has also been checked and approved by a research ethics committee (North West - Liverpool Central Research Ethics Committee). The ethics committee ensures that when you take part in the study, your rights and wellbeing will be protected. The study has also been checked by the government Medicines and Healthcare products Regulatory Agency (MHRA). The MHRA are responsible for approving all studies involving medicines. The Health Research Authority gives final overall approval for the study. The Newcastle upon Tyne Hospitals NHS Foundation Trust is the study Sponsor, which means that they have overall responsibility for the study. The Sponsor has carefully checked all of the study documentation. The Sponsor has also assessed the risks of this study. This is to ensure that we are not doing anything harmful to you during the study and that your information is collected safely and stored securely.

What happens if relevant new information becomes available?

For peer review only - http://bmjopen.bmj.com/site/about/guidelines.xhtml

Newcastle University

<local NHS Trust logo>

During the course of the study, if new information on the risks or benefits of metformin becomes available, we will let you know at your next study visit. If this new information requires urgent action, we will contact you before your next visit. If necessary, we will then discuss whether you should or would like to withdraw from the study.

Will you tell my GP that I'm taking part in a clinical study?

With your permission, we will write to your GP to tell them that you are taking part in this study. Your hospital medical record will also show that you are taking part in a clinical study. It is important for your safety that that your GP practice and hospital medical records show that you took part in a clinical study. If we discover a new health problem during the study, we will tell you. With your permission, we will also tell your GP. Any blood test results from taking part in this study will also be added to your medical records. Your GP will be asked to let the study team know of any side effects from taking the medicine or if you have had any emergency hospital admissions.

Your GP will not know if you have received active treatment (metformin) or placebo until after the end of the study.

Who has overall responsibility for the study?

The study Sponsor is the Newcastle upon Tyne Hospitals NHS Foundation Trust, who have overall responsibility for the study.

The doctor in charge of the study (the Chief Investigator) is Professor Miles Witham, a Consultant Geriatrician based in Newcastle upon Tyne.

The Newcastle University Clinical Trials Unit manages the study on behalf of the Sponsor.

Who is providing the study drug?

We have paid a company called ModePharma to make the metformin and placebo (dummy) tablets for this study. ModePharma specialise in making medicines for studies but they do not own the rights to make Metformin. ModePharma do not make profits by selling metformin. Your hospital pharmacy will give you the study medication (metformin or placebo) at the

For peer review only - http://bmjopen.bmj.com/site/about/guidelines.xhtml

Newcastle University <local NHS Trust logo>

end of the baseline visit. If your visit takes place at home, the tablets will be posted to you by Royal Mail Special Delivery or delivered by local courier. We will ask for your permission to send your name and address to the Royal Mail or the local courier.

What will happen to the results of the MET-PREVENT study?

- The results will be published in medical journals and presented at meetings to other doctors, nurses, researchers and patients.
- A report will be written for the study funder.
- A report must be written for the European Union Drug Regulating Authorities Clinical Trials (EudraCT) database.
- All study results that are published will be anonymous. This means that no-one will be able to find out who you are. Your identity will always be protected.
- The results will be available at the end of the study through publications, in the wider press and directly to patient groups.
- Fully anonymised data may be made available to other researchers to help inform other research studies.

What if I have a complaint or a problem occurs?

a) Complaints

If you have any concern or complaint about any aspect of this clinical study, please contact your local study team by phone, letter or email. Their contact details are listed at the end of this information sheet. If you are still unhappy and wish to raise your concerns with someone who is not directly involved in your care, you can contact <site to localise with local details such as local PALS phone number and email address>

You may also contact Patient Advice and Liaison Service (PALS) for confidential advice on any aspect of care on 0800 032 0202.

b) Harm

For peer review only - http://bmjopen.bmj.com/site/about/guidelines.xhtml

<local NHS Trust logo>

In the unlikely event that you are harmed during the study and this is due to NHS staff neglect, you may have grounds for legal action and compensation. This is organised through the NHS Indemnity (insurance) scheme. You may need to pay for your own legal costs. NHS Indemnity does not offer no-fault compensation (for harm that is not anyone's fault).

The Newcastle Clinical Trials Unit, part of Newcastle University, are managing the study on behalf of the study NHS Sponsor. Newcastle University also have indemnity arrangements. This covers Newcastle University staff involved in designing and managing the MET-PREVENT study.

For peer review only

Will my taking part in the study be kept confidential?

Yes. All the information that you provide during the course of this study will be securely stored. Paper copies of your study information will be stored in locked files or rooms at your local hospital. Electronic copies of your study information will be stored on a secure, password-protected computer database provided by Sealed Envelope™. Only authorised members of the study team will be granted access to the database.

- At study visits, your name will not be written on completed test forms or questionnaires. Instead, we will use a study code number (called a Participant Unique Study Identifier). This number is unique for you. No-one else taking part in the study will have this number. This number will also be used in the study database. Only the study team at your hospital will be able to link this number back to you using your date of birth, name and NHS number
- The study team at your hospital will have access to your information during the study. They will use this information to contact you to organise study visits as well as for ongoing safety.
- If you opt for study visits to take place in your own home, your hospital pharmacy will have access to your contact details to post the study medicine
- Your contact details will never be shared with anyone outside of the study. The exception is Royal Mail or a local courier. You will be asked to consent to the postal service or local courier your local hospital uses having access to your contact details. This is so that they can deliver the

For peer review only - http://bmjopen.bmj.com/site/about/guidelines.xhtml

<local NHS Trust logo>

study medicine to your home address. The postal or courier services will not know you are in the study, just that they need to deliver a package to you

- You will not be named in any results, reports or on websites
- Very occasionally, information might be given during the study that, by law, we must pass on to others. For instance, information which suggested you or others were at risk of harm. In this case, confidentiality would be broken so that we could pass this information to the relevant people. You would be informed of this.
- At the end of the study, all study information will be kept in a secure storage area for at least 15 years. This is called archiving. Archiving means that any queries about the running of the study can still be answered after the study has ended. All information will be held securely to make sure we protect your confidentiality. After the archiving period has ended, your information will be safely destroyed.
- If there are any unexpected serious side effects to the medicine, we would send details of this to the government medicines agency (MHRA). There is a specific form to do this, and only your study number will be sent to them.

Will you look at information from my existing medical records?

Yes. The study team at your hospital will be able to look at your GP and hospital medical records. They need to do this to collect information that is needed for you to take part in the study. For example, they will collect results of your blood tests, and prescriptions and health history.

Authorised people from your local NHS Trust, the MHRA, Sponsor (Newcastle upon Tyne Hospitals NHS Foundation Trust) and/or the Newcastle Clinical Trials Unit will also need to look at your medical records. This is to check that the study is being carried out to the correct standards. Everyone who looks at your medical records will have a duty of confidentiality to you as a research participant.

What will happen if I don't want to carry on with the study?

For peer review only - http://bmjopen.bmj.com/site/about/guidelines.xhtml

<local NHS Trust logo>

You can withdraw from the study completely at any time, for any reason. You do not have to tell the study team why you want to withdraw. You will always be fully cared for and supported in line with your GP and hospital's standard practice.

If you do give a reason for withdrawing from the study, we will ask if you are happy for us to record why you decided to withdraw.

If you withdraw from the study, we will keep the information about you that we have already collected. You are free to request that the study team destroys all information donated by you. By destroying your information, this means that it cannot be used at all for the remainder of the study. However, if some of your information has already been used in calculations and reports, it would not be possible to remove that information from these.

If I stop taking the medicine, do I have to leave the trial?

If you become unwell, your doctor may ask you to stop taking the study medicine. This may be just for a short while until it is safe for you to start taking it again.

You can stop taking the study medicine altogether, but stay in the study and continue with the study visits and assessments. We will always ask you what you would prefer to do.

What happens if I lose the capacity to consent during the study?

During the study, if you lose the capacity to make your own decisions, we will stop your medicine. The doctor may decide that you need to be withdrawn from the study. We will keep your information that has been collected up to this point.

If your doctor thinks you have recovered and can make your own decisions again, we will ask you if you want to continue to take part in the study.

Who is funding the MET-PREVENT study?

For peer review only - http://bmjopen.bmj.com/site/about/guidelines.xhtml

Newcastle University

<local NHS Trust logo>

The National Institute for Health Research (NIHR) Newcastle Biomedical Research Centre funds the study. The UK government funds the NIHR to carry out research for the benefit of the NHS and its patients.

How have patients and the public been involved in the design of the study?

Volunteers from the local VOICE organisation (https://www.voice-global.org/) helped to design this study. They have also looked at the patient information sheet. We will ask a member of the public to join our study management group meetings.

Will my expenses be reimbursed?

Yes – we will pay for your travel expenses including providing a taxi if you need this to attend study visits at the hospital. Alternatively, transport may be arranged for you if your local hospital is able to offer this. Your local study team will manage any payments to reimburse costs to you and you may be asked to provide receipts for your travel.

Will I be paid for taking part?

We will not give you a payment for taking part, but we will pay for your transport and make sure that you have some food and drink at each trial visit.

HOW WILL WE USE INFORMATION ABOUT YOU?

Where 'we' is stated below, this means the study Sponsor – the Newcastle upon Tyne Hospitals NHS Foundation Trust.

We will need to use information from you, your medical records and your GP for this clinical research study.

This information will include your initials, date of birth, NHS number, name and contact details. People will use this information to do the research or to check your records to make sure that the research is being done properly.

For peer review only - http://bmjopen.bmj.com/site/about/guidelines.xhtml

Newcastle University

<local NHS Trust logo>

People who do not need to know who you are will not be able to see your name or contact details. Your data will have a code number, called your unique study identifier, instead.

We will keep all information about you safe and secure.

Once we have finished the study, we will keep some of the data so we can check the results. We will write our reports in a way that no-one can work out that you took part in the study.

The Sponsor will provide Royal Mail or a local courier with your name and the address to allow Royal Mail or the courier to deliver your study medicine to you. Royal Mail and the local courier have their own policies about keeping personal information that comply with UK law. This also covers how they destroy your information. The policy for the Royal Mail Group and any local courier is to only keep information for as long as it is required for the purpose for which they use it.

What are your choices about how your information is used?

- You can stop being part of the study at any time, without giving a reason, but we will keep information about you that we already have.
- We need to manage your records in specific ways for the research to be reliable. This means that we won't be able to let you see or change the data we hold about you.
- If your medical condition does not affect your existing insurance policies, then taking part in a clinical trial should not affect your insurance. For existing insurance, or if you make a new application for any kind of insurance, then you must answer the insurer's questions honestly and accurately.

Where can you find out more about how your information is used?

You can find out more about how we use your information:

- www.hra.nhs.uk/information-about-patients/

For peer review only - http://bmjopen.bmj.com/site/about/guidelines.xhtml

<local NHS Trust logo>

- our leaflet available from https://www.newcastle-hospitals.nhs.uk/help/privacy/privacy-notice-for-patients/ and https://www.qegateshead.nhs.uk/research
- by asking one of the study team
- by sending an email to the trial Sponsor Data Protection Officer at nuth.dpo@nhs.net

Further Information and contact details

If you have any further questions or would like further information about the study or rights of participants, please feel free to contact the people below.

They are also who you or a doctor should contact in the event of an emergency.

<insert local Research Nurse name and contact details>

<insert local site PI name and contact details>

Thank you for reading this information sheet

FUNDED BY NIHR | National Institute for Health Research

This research is funded by the National Institute for Health (NIHR) Newcastle Biomedical Research Centre (BRC). The views expressed are those of the author(s) and not necessarily those of the NIHR or the Department of Health and Social Care.

For peer review only - http://bmjopen.bmj.com/site/about/guidelines.xhtml

SPIRIT
STANDARD PROTOCOL ITEMS: RECOMMENDATIONS FOR INTERVENTIONAL TRIALS

SPIRIT 2013 Checklist: Recommended items to address in a clinical trial protocol and related documents*

Section/item	ItemNo	Description	Page
**Administrative information**			
Title	1	Descriptive title identifying the study design, population, interventions, and, if applicable, trial acronym	1
Trial registration	2a	Trial identifier and registry name. If not yet registered, name of intended registry	3
	2b	All items from the World Health Organization Trial Registration Data Set	3
Protocol version	3	Date and version identifier	20
Funding	4	Sources and types of financial, material, and other support	23
Roles and responsibilities	5a	Names, affiliations, and roles of protocol contributors	23
	5b	Name and contact information for the trial sponsor	19
	5c	Role of study sponsor and funders, if any, in study design; collection, management, analysis, and interpretation of data; writing of the report; and the decision to submit the report for publication, including whether they will have ultimate authority over any of these activities	19
	5d	Composition, roles, and responsibilities of the coordinating centre, steering committee, endpoint adjudication committee, data management team, and other individuals or groups overseeing the trial, if applicable (see Item 21a for data monitoring committee)	18,24
**Introduction**			

For peer review only

Background and rationale	6a	Description of research question and justification for undertaking the trial, including summary of relevant studies (published and unpublished) examining benefits and harms for each intervention	5-7
	6b	Explanation for choice of comparators	5-7
Objectives	7	Specific objectives or hypotheses	5-7
Trial design	8	Description of trial design including type of trial (eg, parallel group, crossover, factorial, single group), allocation ratio, and framework (eg, superiority, equivalence, noninferiority, exploratory)	5-7
**Methods: Participants, interventions, and outcomes**			
Study setting	9	Description of study settings (eg, community clinic, academic hospital) and list of countries where data will be collected. Reference to where list of study sites can be obtained	7
Eligibility criteria	10	Inclusion and exclusion criteria for participants. If applicable, eligibility criteria for study centres and individuals who will perform the interventions (eg, surgeons, psychotherapists)	7-9, Table 1
Interventions	11a	Interventions for each group with sufficient detail to allow replication, including how and when they will be administered	9
	11b	Criteria for discontinuing or modifying allocated interventions for a given trial participant (eg, drug dose change in response to harms, participant request, or improving/worsening disease)	10
	11c	Strategies to improve adherence to intervention protocols, and any procedures for monitoring adherence (eg, drug tablet return, laboratory tests)	Table 2
	11d	Relevant concomitant care and interventions that are permitted or prohibited during the trial	9-10

For peer review only - http://bmjopen.bmj.com/site/about/guidelines.xhtml

Outcomes	12	Primary, secondary, and other outcomes, including the specific measurement variable (eg, systolic blood pressure), analysis metric (eg, change from baseline, final value, time to event), method of aggregation (eg, median, proportion), and time point for each outcome. Explanation of the clinical relevance of chosen efficacy and harm outcomes is strongly recommended	10-11
Participant timeline	13	Time schedule of enrolment, interventions (including any run-ins and washouts), assessments, and visits for participants. A schematic diagram is highly recommended (see Figure)	Table 2
Sample size	14	Estimated number of participants needed to achieve study objectives and how it was determined, including clinical and statistical assumptions supporting any sample size calculations	15
Recruitment	15	Strategies for achieving adequate participant enrolment to reach target sample size	15-16
**Methods: Assignment of interventions (for controlled trials)**			
Allocation:			
Sequence generation	16a	Method of generating the allocation sequence (eg, computer-generated random numbers), and list of any factors for stratification. To reduce predictability of a random sequence, details of any planned restriction (eg, blocking) should be provided in a separate document that is unavailable to those who enrol participants or assign interventions	16
Allocation concealment mechanism	16b	Mechanism of implementing the allocation sequence (eg, central telephone; sequentially numbered, opaque, sealed envelopes), describing any steps to conceal the sequence until interventions are assigned	16
Implementation	16c	Who will generate the allocation sequence, who will enrol participants, and who will assign participants to interventions	16

Blinding (masking)	17a	Who will be blinded after assignment to interventions (eg, trial participants, care providers, outcome assessors, data analysts), and how	16
	17b	If blinded, circumstances under which unblinding is permissible, and procedure for revealing a participant's allocated intervention during the trial	16
**Methods: Data collection, management, and analysis**			
Data collection methods	18a	Plans for assessment and collection of outcome, baseline, and other trial data, including any related processes to promote data quality (eg, duplicate measurements, training of assessors) and a description of study instruments (eg, questionnaires, laboratory tests) along with their reliability and validity, if known. Reference to where data collection forms can be found, if not in the protocol	16-17
	18b	Plans to promote participant retention and complete follow-up, including list of any outcome data to be collected for participants who discontinue or deviate from intervention protocols	16-17
Data management	19	Plans for data entry, coding, security, and storage, including any related processes to promote data quality (eg, double data entry; range checks for data values). Reference to where details of data management procedures can be found, if not in the protocol	16-17
Statistical methods	20a	Statistical methods for analysing primary and secondary outcomes. Reference to where other details of the statistical analysis plan can be found, if not in the protocol	17
	20b	Methods for any additional analyses (eg, subgroup and adjusted analyses)	17-18
	20c	Definition of analysis population relating to protocol non-adherence (eg, as randomised analysis), and any statistical methods to handle missing data (eg, multiple imputation)	18
**Methods: Monitoring**			

Data monitoring	21a	Composition of data monitoring committee (DMC); summary of its role and reporting structure; statement of whether it is independent from the sponsor and competing interests; and reference to where further details about its charter can be found, if not in the protocol. Alternatively, an explanation of why a DMC is not needed	18,24
	21b	Description of any interim analyses and stopping guidelines, including who will have access to these interim results and make the final decision to terminate the trial	N/A
Harms	22	Plans for collecting, assessing, reporting, and managing solicited and spontaneously reported adverse events and other unintended effects of trial interventions or trial conduct	18-19
Auditing	23	Frequency and procedures for auditing trial conduct, if any, and whether the process will be independent from investigators and the sponsor	17
**Ethics and dissemination**			
Research ethics approval	24	Plans for seeking research ethics committee/institutional review board (REC/IRB) approval	19-20
Protocol amendments	25	Plans for communicating important protocol modifications (eg, changes to eligibility criteria, outcomes, analyses) to relevant parties (eg, investigators, REC/IRBs, trial participants, trial registries, journals, regulators)	19-20
Consent or assent	26a	Who will obtain informed consent or assent from potential trial participants or authorised surrogates, and how (see Item 32)	20
	26b	Additional consent provisions for collection and use of participant data and biological specimens in ancillary studies, if applicable	20
Confidentiality	27	How personal information about potential and enrolled participants will be collected, shared, and maintained in order to protect confidentiality before, during, and after the trial	20
Declaration of interests	28	Financial and other competing interests for principal investigators for the overall trial and each study site	23

For peer review only

Access to data	29	Statement of who will have access to the final trial dataset, and disclosure of contractual agreements that limit such access for investigators	20
Ancillary and post-trial care	30	Provisions, if any, for ancillary and post-trial care, and for compensation to those who suffer harm from trial participation	20
Dissemination policy	31a	Plans for investigators and sponsor to communicate trial results to participants, healthcare professionals, the public, and other relevant groups (eg, via publication, reporting in results databases, or other data sharing arrangements), including any publication restrictions	21
	31b	Authorship eligibility guidelines and any intended use of professional writers	21
	31c	Plans, if any, for granting public access to the full protocol, participant-level dataset, and statistical code	20-21
**Appendices**			
Informed consent materials	32	Model consent form and other related documentation given to participants and authorised surrogates	Not supplied
Biological specimens	33	Plans for collection, laboratory evaluation, and storage of biological specimens for genetic or molecular analysis in the current trial and for future use in ancillary studies, if applicable	Not supplied

*It is strongly recommended that this checklist be read in conjunction with the SPIRIT 2013 Explanation & Elaboration for important clarification on the items. Amendments to the protocol should be tracked and dated. The SPIRIT checklist is copyrighted by the SPIRIT Group under the Creative Commons "Attribution-NonCommercial-NoDerivs 3.0 Unported" license.

For peer review only
